# The rheumatoid arthritis gut microbial biobank reveals core microbial species that associate and effect on host inflammation and autoimmune responses

**DOI:** 10.1002/imt2.242

**Published:** 2024-10-03

**Authors:** Hao‐Jie Huang, Chang Liu, Xin‐Wei Sun, Rui‐Qi Wei, Ling‐Wei Liu, Hao‐Yu Chen, Rashidin Abdugheni, Chang‐Yu Wang, Xiao‐Meng Wang, He Jiang, Han‐Yu Niu, Li‐Juan Feng, Jia‐Hui He, Yu Jiang, Yan Zhao, Yu‐Lin Wang, Qiang Shu, Ming‐Xia Bi, Lei Zhang, Bin Liu, Shuang‐Jiang Liu

**Affiliations:** ^1^ State Key Laboratory of Microbial Technology Shandong University Qingdao China; ^2^ Department of Rheumatology The Affiliated Hospital of Qingdao University Qingdao China; ^3^ State Key Laboratory of Desert and Oasis Ecology, Key Laboratory of Ecological Safety and Sustainable Development in Arid Lands Xinjiang Institute of Ecology and Geography, Chinese Academy of Sciences Ürűmqi China; ^4^ School of Life Sciences, Division of Life Sciences and Medicine University of Science and Technology of China Hefei China; ^5^ College of Veterinary Medicine Shanxi Agricultural University Taigu China; ^6^ Biomedical Sciences College & Shandong Medicinal Biotechnology Centre Shandong First Medical University & Shandong Academy of Medical Sciences Jinan China; ^7^ Department of Rheumatology Qilu Hospital, Cheeloo College of Medicine, Shandong University Jinan China; ^8^ Microbiome‐X School of Public Health, Cheeloo College of Medicine, Shandong University Jinan China; ^9^ State Key Laboratory of Microbial Resources Institute of Microbiology, Chinese Academy of Sciences Beijing China

**Keywords:** core microbial species, *Eubacterium rectale*, inflammatory and immune responses, *Mediterraneibacter tenuis*, RA clinical indices, RA‐originated gut microbial biobank (RAGMB), rheumatoid arthritis (RA)

## Abstract

Gut microbiota dysbiosis has been implicated in rheumatoid arthritis (RA) and influences disease progression. Although molecular and culture‐independent studies revealed RA patients harbored a core microbiome and had characteristic bacterial species, the lack of cultured bacterial strains had limited investigations on their functions. This study aimed to establish an RA‐originated gut microbial biobank (RAGMB) that covers and further to correlates and validates core microbial species on clinically used and diagnostic inflammation and immune indices. We obtained 3200 bacterial isolates from fecal samples of 20 RA patients with seven improved and 11 traditional bacterial cultivation methods. These isolates were phylogenetically identified and selected for RAGMB. The RAGMB harbored 601 bacterial strains that represented 280 species (including 43 novel species) of seven bacterial phyla. The RAGMB covered 93.2% at species level of medium‐ and high‐abundant (relative abundances ≥0.2%) RA gut microbes, and included four rare species of the phylum *Synergistota*. The RA core gut microbiome was defined and composed of 20 bacterial species. Among these, *Mediterraneibacter tenuis* and *Eubacterium rectale* were two species that statistically and significantly correlated with clinically used diagnostic indices such as erythrocyte sedimentation rate (ESR) and IL‐10. Thus, *M. tenuis* and *E. rectale* were selected for experimental validation using DSS‐treated and not DSS‐treated mice model. Results demonstrated both *M. tenuis* and *E. rectale* exacerbated host inflammatory responses, including shortened colon length and increased spleen weight, decreased IL‐10 and increased IL‐17A levels in plasma. Overall, we established the RAGMB, defined the RA core microbiome, correlated and demonstrated core microbial species effected on host inflammatory and immune responses. This work provides diverse gut microbial resources for future studies on RA etiology and potential new targets for new biomedical practices.

## Highlights

- An rheumatoid arthritis (RA)‐originated gut microbial biobank (RAGMB) was established, comprising 601 bacterial strains representing 280 species (including 43 novel species) across seven bacterial phyla.
- RAGMB covers 93.2% of medium‐ and high‐abundant RA gut microbe species from isolated samples.
- The RA core microbiome consists of 20 bacterial species, with *Mediterraneibacter tenuis* and *Eubacterium rectale* showing significant correlations with clinical indices such as ESR and IL‐10.
- RA core species *Mediterraneibacter tenuis* and *Eubacterium rectale* exacerbate inflammatory responses, including shortened colon length, enlarged spleen and altered plasma cytokine levels.

## Graphical abstract

Intensive collection of 3200 bacterial isolates from fecal samples of newly diagnosed rheumatoid arthritis (RA) patients resulted in an RA‐originated gut microbial biobank (RAGMB). This RAGMB has 601 strains that represent 280 bacterial species (including 43 novel species), and covers 93.2% of medium‐ and high‐abundant gut microbial species of the RA fecal samples. By integrating additional RA cohort metagenomic data, an RA core microbiome composing of 20 core microbial species were defined and correlated to RA clinical indices. Two RA core microbial species, *Mediterraneibacter tenuis* and *Eubacterium rectale*, were selected for experimental validation with mouse models and the results showed that both *M. tenuis* and *E. rectale* exacerbated host inflammatory and immune responses.

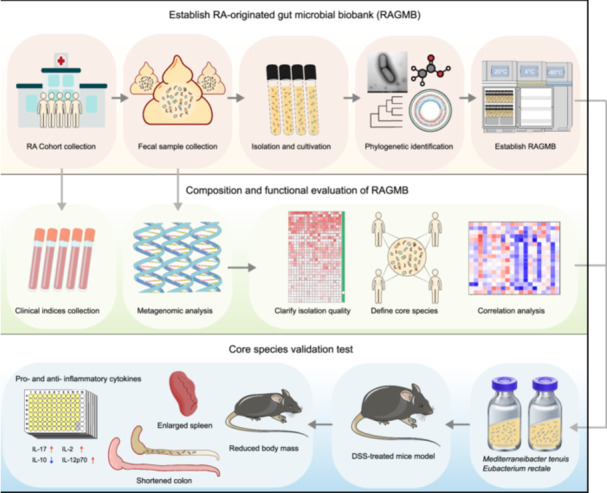

## INTRODUCTION

Rheumatoid arthritis (RA) is an inflammatory autoimmune disease associated with progressive disability, systemic complications, premature death, and socioeconomic costs [1, 2]. The etiology and the prognosis of RA remain unclear, but host genetics, gut microbiota, and environments contribute to RA pathogenesis [1, 3, 4]. More and more evidence support that gut microbiota plays an important role in the etiology and the progression of RA. Earlier studies showed alteration and dysbiosis of the gut microbiota in RA patients [5, 6, 7, 8]. It is widely accepted that dysbiosis of the gut microbiota induced arthritis by affecting the differentiation of immune cell subsets. With pro‐inflammatory cells development, a localized inflammatory cascade leads to tissue damage and systemic autoimmunity [3, 9]. Studies have disclosed that gut microbes and their metabolites modulate autoimmune responses in animal models of arthritis [5, 6, 10], host immune responses, and virulence proteins as well [11, 12, 13]. *Prevotella copri* (*Pvt. copri*) was enriched in RA patients and *Fusobacterium nucleatum* aggravated RA symptoms [8, 11, 12], and *Parabacteroides distasonis* decreased in RA patients [14]. More recently, gut microbiota has been considered as a new target for the medical practices of arthritis [15].

Culture‐independent, metagenomic studies have contributed extraordinarily to the understanding of gut microbiota and host RA associations [16, 17, 18, 19]. Still, the investigations on causality and mechanism of gut microbes‐host interactions require cultured microbial resources [20]. In recent years, efforts have been made on the cultivation and collection of gut microbial strains and on the establishment of gut microbial biobanks (GMBs), such as the BIO‐ML [21], CGR [22], CULTUROMICS [23], HBC [24], and the healthy human GMB (hGMB) [25]. The GMBs have contributed to causative and mechanistic investigations, for examples, strains from the hGMB were applied to studies of gut microbiota with obesity [26], cardiovascular disease [27], nonalcoholic steatohepatitis [28] and autism spectrum disorder [29]. With the exception of CULTUROMICS that collected microbial strains from patients (e.g., anorexia nervosa, obesity, malnutrition and HIV) [23], microbial strains deposited in the established GMBs were originated from healthy donors [21, 22, 24, 25]. Thus, microbial strains from patients are by far underrepresented in the repertoires of established GMBs. On the other hand, studies revealed that disease populations had gut microbiota at species and strain levels different from that of healthy populations [30, 31, 32, 33, 34]. For example, the *Pvt. copri* strain from the gut of RA patients had different genes when compared to the *Pvt. copri* strain from healthy individuals, and the difference might contribute to RA onset [6, 12].

In this study, we established an RA‐originated gut microbial biobank (RAGMB). For easy access to the public, we deposited 601 representative strains at the China General Microbiological Culture Collection Center (CGMCC) of the International Depositary Authority (IDA). The RAGMB covered extensively both high and low‐abundant RA gut microbes and included four rare species from the phylum *Synergistota*. We defined an RA core gut microbiome composed of 20 bacterial species, and correlated core species with RA clinically used indices and prognosis. Finally, we found that two core microbial species *Mediterraneibacter tenuis* and *Eubacterium rectale* exacerbated host inflammatory responses in mice.

## RESULTS

### Bacterial cultivation, construction of RAGMB, and 43 novel bacterial species from RA patients

Many gut microbes are resistant to be grown in lab, and targeted cultivation of microbial species is even more challenging. We made extensive efforts on cultivation of gut microbes from RA fecal samples by (1) mining genome data and extraction of relevant information merited for bacterial growth. A new medium called mX (Table S1) with xylan as sole carbon source was designed for the cultivation of *Prevotella* and *Bacteroides* species that were reportedly enriched in RA [35, 36]; (2) applying diverse culture media, multiple sample pretreatments, and different culture conditions to improve the diversities of cultured bacterial strains. Totally, we applied 18 methods/combinations used in this study, including seven improved and 11 traditional methods (Table S2). With those methods, we obtained 3200 microbial isolates from 20 fecal donors of RA patients (Table S3).

**Figure 1:**
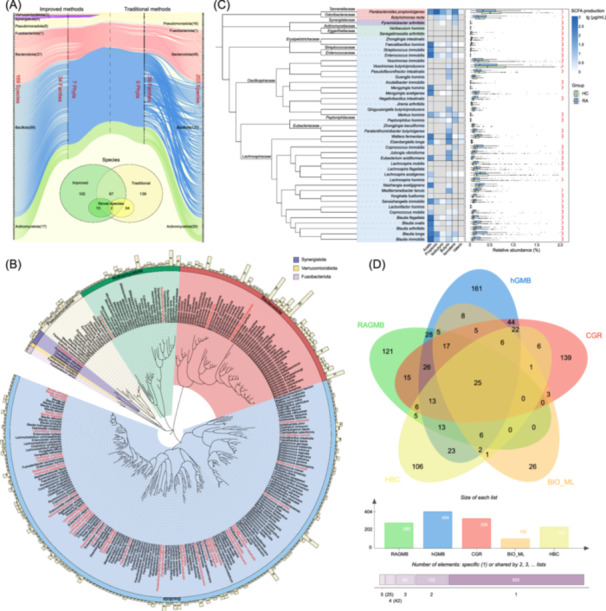
Construction of RAGMB with large‐scale bacterial isolation and cultivation. (A) the right part shows 203 species of 39 families in five phyla isolated with the 11 traditional methods, and the left part shows 169 species of 34 families in seven phyla with seven improved methods. Venn diagram shows the difference in the composition of species isolated by the two types of methods. (B) shows the full range of 601 strains preserved in the RAGMB. The background of the branching diagrams is coded according to the seven phyla. The outer ring bars indicate the number of preserved strains for each species. Species in red represent novel species nominated in this study. (C) shows the production of short‐chain fatty acids with 43 novel species in RAGMB, and the abundances of the 43 novel species in the RA cohort (*n* = 96) and healthy population (*n* = 1153). The differences of abundance between RA and healthy populations were assessed with the Wilcoxon non‐parametric test. The symbols ***, **, and * represent *p*‐values < 0.001, 0.01, and 0.05 respectively. (D) Wayne's diagram showing the five large gut microbial biobanks and overlapping numbers at species level.

All 3200 isolates were sequenced for 16S rRNA genes, and were classified into seven phyla, that is, *Bacillota*, *Bacteroidota*, *Actinomycetota*, *Pseudomonadota*, *Synergistota*, *Verrucomicrobiota*, and *Fusobacteriota*. Using our previously established minimal polyphasic taxonomy identification procedure [25], those bacterial isolates were taxonomically identified. Results showed that 169 species were cultivated with the seven improved methods and they covered all the seven phyla including species of the *Synergistota*, *Fusobacteriota*, and *Verrucomicrobiota* that occurred at low‐abundance in gut microbiomes. In contrast, 203 species of five phyla were obtained with the 11 traditional methods. There were 67 species from both improved and traditional methods, thus we obtained 305 different species (Figure 1A). Twenty‐five species were lost during subsequent cultivation. Finally, we successfully deposited 601 strains representing 280 species in CGMCC, and termed this collection of 601 strains as the RAGMB. The 601 strains (280 species) in RAGMB covered 135 genera of 36 families in seven different phyla (Figure 1B, Table S4), among which there were 43 novel species. More information (the taxonomy and 16S rRNA gene sequences; phenotypic and genomic descriptions for all new species) regarding the 280 species is accessible at the RAGMB website (https://www.nmdc.cn/ragmb/).

The 43 novel species were genome‐sequenced and their phenotypes were characterized and are described in Table 1 (see “Supplementary Taxon” for more results). They belonged to 33 genera (including 11 newly proposed genera) and 13 families. Their genome sizes ranged from 1.87 to 7.04 Mb, and GC contents ranged from 30.3% to 62.1% (Table S5). The cellular morphology of these novel species varied, including long and short rod, fusiform, oval‐shaped, spherical, or coccoid. The *Waltera fermentans* HA1509^T^ was featured by forming spherical structure with a diameter of approximately 1.5 μm in the middle of cells (Supplementary Taxon, Figure ST‐35D). The productions of short‐chain fatty acids (SCFAs) by these species were determined. The results revealed that the five novel *Blautia* species (*Blt. arthitidis*, *Blt. flagellata*, *Blt. immobilis, Blt. longa* and *Blt. ovalis*), *Eisenbergiella longa*, *Faecalibacillus hominis*, *Waltera fermentan* and many other novel species were productive for acetic acid, *Parabacteroides propionicigenes* and *Blt. longa* were productive for propionic acid, *Eisenbergiella hominis* and *Blt. longa* were productive for both acetic and butyric acids, and *Parabacteroides propionicigenes*, *Butyricimonas recta*, and *Pyramidobacter arthritidis* produced isovaleric acids (Figure 1C, more data of SCFAs production are shown in Table S6). Among them, *Blt. immobilis* strain HA0440^T^ exhibited the highest production of acetic acid (751.7 μg/ml). *Parabacteroides acetigenes* strain HA3406^T^ exhibited the highest production of propionic acid (1100.3 μg/ml), *Waltera fermentans* strain HA1509^T^ exhibited the highest production of butyric acid (99.3 μg/ml). These novel species were distributed not only in the RA cohort (*n* = 96) of this study, but were also widely in healthy populations (*n* = 1153). The prevalences of the 43 novel species in healthy populations ranged from 0.08% to 99.2%. Statistically, 35 novel species showed significantly different distribution between the RA cohort and the healthy populations (Figure 1C, Tables S7 and S8).

**Table 1:** The protologs of 43 novel taxa in rheumatoid arthritis‐originated gut microbial biobank (RAGMB).

Taxonomy	Etymology	Description
*Zhonglingia intestinalis* gen. nov. sp. nov.	Zhong.ling'i.a. N.L. fem. n. Zhonglingia, named in honor of the Chinese medical scientist Zhongling Cheng. in.tes.ti.na'lis. N.L. fem. adj. *intestinalis*, pertaining to the intestine, denoting the type strain was isolated from the intestine.	Cells are rod‐shaped with spiky ends, without flagella and nonmotile. Growth to stable phase occurs after 48 h incubation in mGAM medium at 37°C, pH = 7.2. No significant fermentation products detected. The genomic DNA G + C content of the type strain is 38.7 mol%. The type strain HA1519^T^ (=CGMCC 1.48484^T^ = KCTC 25719^T^) was isolated from the feces of rheumatoid arthritis patients.
*Paralentihominibacter butyricigenes* gen. nov. sp. nov.	Pa.ra.len.ti.ho.mi.ni.bac'ter. Gr. prep. para, next to; N.L. masc. n. *Lentihominibacter*, a bacterial genus; N.L. masc. n. *Paralentihominibacter*, a genus closely related to *Lentihominibacter*. bu.ty.ri.ci'ge.nes. N.L. masc. n. *acidum butyricum*, butyric acid; Gr. suff. ‐*genes*, forming; N.L. part. adj. *butyricigenes*, butyric‐acid producing, denoting the type strain produces butyric acid.	Cells are rod‐shaped rounded ends, without flagella and nonmotile. Growth to stable phase occurs after 48 h incubation in mGAM medium at 37°C, pH = 7.2. The main fermentation product is butyric acid. The genomic DNA G + C content of the type strain is 44.26 mol%. The type strain HA0442^T^ (=CGMCC 1.48215^T^ = KCTC 25743^T^) was isolated from the feces of rheumatoid arthritis patients.
*Zhongjingia baculiformis* gen. nov. sp. nov.	Zhong.jing'i.a. N.L. fem. n. *Zhongjingia*, named after Zhongjing Zhang, a medical scientist who has contributed to the treatment of rheumatic arthritis using traditional Chinese medicine. ba.cu.li.for'mis. L. fem. n. *baculus*, rod; L. fem. adj. suff. ‐*formis*, of the shape of; N.L. fem. adj. *baculiformis*, rod‐shaped, denoting the shape of the type strain of the species.	Cells are rod‐shaped with oval ends, without flagella and nonmotile. Growth to stable phase occurs after 72 h incubation in mGAM medium at 37°C, pH = 7.2. The main fermentation product is small amount of butyric acid. The genomic DNA G + C content of the type strain is 45.59 mol%. The type strain HA0628^T^ (=CGMCC 1.48322^T^) was isolated from the feces of rheumatoid arthritis patients.
*Naizhengia acetigignens* gen. nov. sp. nov.	Nai.zheng'i.a. N.L. fem. n. *Naizhengia*, named in honor of the Chinese medical scientist Naizheng Zhang. a.ce.ti.gi.gnens. L. fem. n. *acetum*, vinegar, refer to acetic acid; L. inf. v. *gignere*, to produce; N.L. part. adj. *acetigignens*, acetic acid‐producing, denoting the type strain produces acetic acid.	Cells are rod‐shaped with square ends, without flagella and nonmotile. Growth to stable phase occurs after 48 h incubation in mGAM medium at 37°C, pH = 7.2. The main fermentation product is acetic acid, and small amount of butyric acid can also be produced. The genomic DNA G + C content of the type strain is 45.11 mol%. The type strain HA0073^T^ (=CGMCC 1.48065^T^ = KCTC 25694^T^) was isolated from the feces of rheumatoid arthritis patients.
*Sanxizhangella immobilis* gen. nov. sp. nov.	San.xi.zhang.el'la. N.L. fem. dim. n. *Sanxizhangella*, named after San‐Xi Zhang, a Chinese medical scientist, in honor of Zhang's contributions regarding the studies and treatment of rheumatoid arthritis. im.mo'bi.lis. L. fem. adj. *immobilis*, immovable, nonmotile, indicating the type strain is nonmotile.	Cells are fusiform, without flagella and nonmotile. Growth to stable phase occurs after 48 h incubation in mGAM medium at 37°C, pH = 7.2. The main fermentation product is acetic acid, and small amount of propionic, isobutyric and butyric acid can also be produced. The genomic DNA G + C content of the type strain is 46.24 mol%. The type strain HA0013^T^ (=CGMCC 1.48011^T^ = KCTC 25673^T^) was isolated from the feces of rheumatoid arthritis patients.
*Yonghella fusiformis* gen. nov. sp. nov.	Yong.hel'la. N.L. fem. dim. n. *Yonghella*, named after Yonghe Yan, a medical scientist who has contributed to the treatment of rheumatic arthritis using traditional Chinese medicine and the author of the medical script “Ji Sheng Fang.” fu.si.for'mis. L. fem. n. *fusus*, spindle; L. fem. n. *forma*, form, shape; N.L. fem. adj. *fusiformis*, spindle‐shaped, denoting the shape of the type strain of the species.	Cells are fusiform, without flagella and nonmotile. Growth to stable phase occurs after 48 h incubation in mGAM medium at 37°C, pH = 7.2. The main fermentation product is small amount of acetic. The genomic DNA G + C content of the type strain is 42.91 mol%. The type strain HA1168^T^ (=CGMCC 1.48350^T^ = KCTC 25681^T^) was isolated from the feces of rheumatoid arthritis patients.
*Guangjia hominis* gen. nov. sp. nov.	Guang.ji.a. N.L. fem. n. *Guangjia*, in honor of Guangji Shi, in recognition of his contributions to the treatment of rheumatic arthritis using traditional Chinese medicine. ho'mi.nis. L. gen. fem. n. *hominis*, of a human being, referring to the human gut habitat of the type strain.	Cells are rod‐shaped with spiky ends, without flagella and nonmotile. Growth to stable phase occurs after 48 h incubation in mGAM medium at 37°C, pH = 7.2. The main fermentation product is butyric acid. The genomic DNA G + C content of the type strain is 61.81 mol%. The type strain HA1523^T^ (=CGMCC 1.17999^T^ = KCTC 25720^T^) was isolated from the feces of rheumatoid arthritis patients.
*Jirenia arthritidis* gen. nov. sp. nov.	Ji.ren'i.a. N.L. fem. n. *Jirenia*, named in honor of the Chinese medical scientist Jiren Li, who did great contribution in the treatment of rheumatoid arthritis. ar.thri'ti.dis. Gr. fem. n. *arthron*, joint; N.L. fem. n. suff. *‐itis*, inflammation; N.L. gen. fem. n. *arthritidis*, of arthritis, denoting the type strain was isolated from the feces of a rheumatoid arthritis patient.	Cells are rod‐shaped with pointed ends, without flagella and nonmotile. Growth to stable phase occurs after 48 h incubation in mGAM medium at 37°C, pH = 7.2. No significant fermentation products detected. The genomic DNA G + C content of the type strain is 53.32 mol%. The type strain HA0569^T^ (=CGMCC 1.48290^T^ = KCTC 25701^T^) was isolated from the feces of rheumatoid arthritis patients.
*Qingyuzengella butyriciproducens* gen. nov. sp. nov.	Qing.yu.zeng.el'la. N.L. fem. dim. n. *Qingyuzengella*, named in honor of the Chinese scientist Qingyu Zeng. bu.ty.ri.ci.pro.du'cens. N.L. fem. n. *acidum butyricum*, butyric acid; L. pres. part. *producens*, producing; N.L. part. adj. *butyriciproducens*, producing butyric acid, denoting the type strain is a butyrate‐producing bacterium.	Cells are rod‐shaped with square ends, without flagella and nonmotile. Growth to stable phase occurs after 48 h incubation in mGAM medium at 37°C, pH = 7.2. The main fermentation product is butyric acid. The genomic DNA G + C content of the type strain is 59.15 mol%. The type strain HA0434^T^ (=CGMCC 1.48207^T^ = KCTC 25712^T^) was isolated from the feces of rheumatoid arthritis patients.
*Mengyingia acetigenes* gen. nov. sp. nov.	Meng.ying'i.a. N.L. fem. n. *Mengyingia*, named in honor of the Chinese medical scientist Mengying Wang. a.ce.ti'ge.nes. L. fem. n. *acetum*, vinegar; Gr. ind. v. *gennaô*, to produce; N.L. gen. fem. adj. *acetigenes*, acetate producing, indicating the type strain produces acetate.	Cells are chained rods with tapered ends, without flagella and nonmotile. Growth to stable phase occurs after 48 h incubation in mGAM medium at 37°C, pH = 7.2. The main fermentation product is acetic acid, and small amount of isobutyric and butyric acid can also be produced. The genomic DNA G + C content of the type strain is 52.16 mol%. The type strain HA1243^T^ (=CGMCC 1.48425^T^ = KCTC 25683^T^) was isolated from the feces of rheumatoid arthritis patients.
*Mengyingia hominis* sp. nov.	ho'mi.nis. L. gen. masc. n. *hominis*, of a human being, indicating that the type strain was isolated from a human.	Cells are rod‐shaped in pairs with pointed ends, without flagella and nonmotile. Growth to stable phase occurs after 24 h incubation in mGAM medium at 37°C, pH = 7.2. The main fermentation product is acetic acid, and small amount of butyric acid can also be produced. The genomic DNA G + C content of the type strain is 58.83 mol%. The type strain HA0445^T^ (=CGMCC 1.48218^T^ = KCTC 25696^T^) was isolated from the feces of rheumatoid arthritis patients.
*Markus hominis* gen. nov. sp. nov.	Mar.k'us. L. masc. n. *Markus*, in honor of the famous microbiologist Markus Göker, whos is a member of the Leibniz Institute DSMZ. ho'mi.nis. L. gen. masc. n. *hominis*, of a human being, indicating that the type strain was isolated from a human.	Cells are rod‐shaped with square ends, without flagella and nonmotile. Growth to stable phase occurs after 48 h incubation in mGAM medium at 37°C, pH = 7.2. The main fermentation product is acetic acid, and small amount of propionic, isobutyric, butyric, isovaleric and valeric acid can also be produced. The genomic DNA G + C content of the type strain is 59.28 mol%. The type strain HA1496^T^ (=CGMCC 1.48461^T^ = KCTC 25785^T^) was isolated from the feces of rheumatoid arthritis patients.
*Acutalibacter immobilis* sp. nov.	im.mo'bi.lis. L. masc. adj. *immobilis*, immovable, motionless, indicating the type strain is nonmotile.	Cells are short rod‐shaped with oval ends, without flagella and nonmotile. Growth to stable phase occurs after 24 h incubation in mGAM medium at 37°C, pH = 7.2. No significant fermentation products detected. The genomic DNA G + C content of the type strain is 52.79 mol%. The type strain HA1516^T^ (=CGMCC 1.17975^T^ = KCTC 25684^T^) was isolated from the feces of rheumatoid arthritis patients.
*Varibaculum hominis* sp. nov.	ho'mi.nis. L. gen. neut. n. *hominis*, of a human being, indicating that the type strain was isolated from a human.	Cells are short rod‐shaped, without flagella and nonmotile. Growth to stable phase occurs after 24 h incubation in mGAM medium at 37°C, pH = 7.2. No significant fermentation products detected. The genomic DNA G + C content of the type strain is 52.56 mol%. The type strain HA1244^T^ (=CGMCC 1.48426^T^ = KCTC 25715^T^) was isolated from the feces of rheumatoid arthritis patients.
*Senegalimassilia arthritidis* sp. nov.	ar.thri'ti.dis. Gr. fem. n. *arthron*, joint; N.L. fem. n. suff. ‐*itis*, inflammation; N.L. gen. fem. n. *arthritidis*, of arthritis, denoting the type strain was isolated from the feces of a rheumatoid arthritis patient.	Cells are coccobacillary with rounded ends, without flagella and nonmotile. Growth to stable phase occurs after 48 h incubation in mGAM medium at 37°C, pH = 7.2. No significant fermentation products detected. The genomic DNA G + C content of the type strain is 62.05 mol%. The type strain HA0643^T^ (=CGMCC 1.48328^T^ = KCTC 25752^T^) was isolated from the feces of rheumatoid arthritis patients.
*Enterococcus immobilis* sp. nov.	im.mo'bi.lis. L. masc. adj. *immobilis*, nonmotile, indicating the non‐motility of the type strain of the species.	Cells are oval‐shaped, without flagella and nonmotile. Growth to stable phase occurs after 24 h incubation in mGAM medium at 37°C, pH = 7.2. The main fermentation product is acetic acid, and small amount of propionic, isobutyric, butyric, isovaleric and valeric acid can also be produced. The genomic DNA G + C content of the type strain is 43.75 mol%. The type strain HA0446^T^ (=CGMCC 1.48219^T^ = KCTC 25678^T^) was isolated from the feces of rheumatoid arthritis patients.
*Streptococcus immobilis* sp. nov.	im.mo'bi.lis. L. masc. adj. *immobilis*, nonmotile, indicating the non‐motility of the type strain of the species.	Cells are oval‐shaped with spiky ends, without flagella and nonmotile. Growth to stable phase occurs after 48 h incubation in mGAM medium at 37°C, pH = 7.2. The main fermentation product is acetic acid, and small amount of butyric acid can also be produced. The genomic DNA G + C content of the type strain is 41.85 mol%. The type strain HA0527^T^ (=CGMCC 1.48250^T^ = KCTC 25679^T^) was isolated from the feces of rheumatoid arthritis patients.
*Faecalibacillus hominis* sp. nov.	ho'mi.nis. L. gen. masc. n. *hominis*, of a human being, indicating that the type strain was isolated from a human.	Cells are short rod‐shaped with square ends, without flagella and nonmotile. Growth to stable phase occurs after 24 h incubation in mGAM medium at 37°C, pH = 7.2. The main fermentation product is acetic acid, and small amount of propionic, isobutyric, butyric, isovaleric and valeric acid can also be produced. The genomic DNA G + C content of the type strain is 30.34 mol%. The type strain HA0003^T^ (=CGMCC 1.48003^T^ = KCTC 25672^T^) was isolated from the feces of rheumatoid arthritis patients.
*Eubacterium acidiformans* sp. nov.	a.ci.di.for'mans. L. neut. adj. *acidum*, an acid; from L. masc. adj. *acidus*, sour; L. pres. part. *formans*, forming; N.L. part. adj. *acidiformans*, acid‐forming, indicating the strain produces acids.	Cells are coccobacillary with rounded ends, without flagella and nonmotile. Growth to stable phase occurs after 48 h incubation in mGAM medium at 37°C, pH = 7.2. The main fermentation products are acetic and butyric acid. The genomic DNA G + C content of the type strain is 32.99 mol%. The type strain HA0433^T^ (=CGMCC 1.48206^T^ = KCTC 25713^T^) was isolated from the feces of rheumatoid arthritis patients.
*Blautia arthritidis* sp. nov.	ar.thri'ti.dis. Gr. fem. n. *arthron*, joint; N.L. fem. n. suff. ‐itis, inflammation; N.L. gen. fem. n. arthritidis, of arthritis, denoting the type strain was isolated from the feces of rheumatoid arthritis patients.	Cells are rod‐shaped with rounded ends, without flagella and nonmotile. Growth to stable phase occurs after 48 h incubation in mGAM medium at 37°C, pH = 7.2. The main fermentation product is acetic. The genomic DNA G + C content of the type strain is 44.17 mol%. The type strain HA0067^T^ (=CGMCC 1.48059^T^ = KCTC 25675^T^) was isolated from the feces of rheumatoid arthritis patients.
*Blautia flagellata* sp. nov.	fla.gel.la'ta. L. fem. n. *flagellum*, a whip; L. fem. adj. suff. *‐ata*, suffix denoting provided with; L. fem. part. adj. *flagellata*, flagellated, denoting the type strain has flagellum.	Cells are rod‐shaped with rounded ends, with flagella, motile. Growth to stable phase occurs after 48 h incubation in mGAM medium at 37°C, pH = 7.2. The main fermentation product is acetic acid, and small amount of isobutyric and butyric acid can also be produced. The genomic DNA G + C content of the type strain is 43.71 mol%. The type strain HA1512^T^ (=CGMCC 1.48477^T^ = KCTC 25718^T^) was isolated from the feces of rheumatoid arthritis patients.
*Blautia immobilis* sp. nov.	im.mo'bi.lis. L. fem. adj. *immobilis*, nonmotile, denoting the non‐motility of the type strain of the species.	Cells are oval‐shaped, without flagella and nonmotile. Growth to stable phase occurs after 48 h incubation in mGAM medium at 37°C, pH = 7.2. The main fermentation products are acetic, propionic acid, and small amount of isobutyric, butyric, isovaleric and valeric acid can also be produced. The genomic DNA G + C content of the type strain is 43.75 mol%. The type strain HA0440^T^ (=CGMCC 1.48213^T^ = KCTC 25742^T^) was isolated from the feces of rheumatoid arthritis patients.
*Blautia longa* sp. nov.	lon'ga. L. fem. adj. *longa*, long in shape, referring to the cell shape of the type strain.	Cells are rod‐shaped with oval ends, without flagella and nonmotile. Growth to stable phase occurs after 48 h incubation in mGAM medium at 37°C, pH = 7.2. The main fermentation products are acetic and butyric acid. The genomic DNA G + C content of the type strain is 44.26 mol%. The type strain HA0030^T^ (=CGMCC 1.48026^T^ = KCTC 25674^T^) was isolated from the feces of rheumatoid arthritis patients.
*Blautia ovalis* sp. nov.	o.va'lis. L. fem. adj. *ovalis*, egg‐shaped, denoting shape of the type strain of the species.	Cells are oval‐shaped with square ends, without flagella and nonmotile. Growth to stable phase occurs after 48 h incubation in mGAM medium at 37°C, pH = 7.2. The main fermentation product is acetic acid, and small amount of propionic and butyric acid can also be produced. The genomic DNA G + C content of the type strain is 43.64 mol%. The type strain HA0435^T^ (=CGMCC 1.48208^T^ = KCTC 25713^T^) was isolated from the feces of rheumatoid arthritis patients.
*Coprococcus immobilis* sp. nov.	im.mo'bi.lis. L. masc. adj. *immobilis*, nonmotile, indicating the non‐motility of the type strain.	Cells are oval‐shaped, without flagella and nonmotile. Growth to stable phase occurs after 48 h incubation in mGAM medium at 37°C, pH = 7.2. The main fermentation product is isovaleric acid. The genomic DNA G + C content of the type strain is 42.94 mol%. The type strain HA0444^T^ (=CGMCC 1.48217^T^ = KCTC 25745^T^) was isolated from the feces of rheumatoid arthritis patients.
*Coprococcus mobilis* sp. nov.	mo'bi.lis. L. masc. adj. *mobilis*, mobile.	Cells are oval‐shaped with spiky ends, with flagella, motile. Growth to stable phase occurs after 48 h incubation in mGAM medium at 37°C, pH = 7.2. The main fermentation products are small amount of acetic, butyric and isovaleric acid. The genomic DNA G + C content of the type strain is 40.00 mol%. The type strain HA0524^T^ (=CGMCC 1.48247^T^) was isolated from the feces of rheumatoid arthritis patients.
*Eisenbergiella longa* sp. nov.	lon'ga. L. fem. adj. *longa*, long in shape, referring to the cell shape of the type strain.	Cells are long rod‐shaped, without flagella and nonmotile. Growth to stable phase occurs after 48 h incubation in mGAM medium at 37°C, pH = 7.2. The main fermentation product is acetic and butyric acid, and small amount of propionic and valeric acid can also be produced. The genomic DNA G + C content of the type strain is 48.23 mol%. The type strain HA0447^T^ (=CGMCC 1.48220^T^ = KCTC 25697^T^) was isolated from the feces of rheumatoid arthritis patients.
*Jutongia vibrioforma* sp. nov.	vi.bri.o.for'ma. L. fem. adj. suff. *‐forma*, of the shape of; N.L. fem. adj. *vibrioforma*, shaped like a curved cell, like a vibrio, indicating the shape of the type strain.	Cells are comma‐shaped with rounded ends, without flagella and nonmotile. Growth to stable phase occurs after 48 h incubation in mGAM medium at 37°C, pH = 7.2. The main fermentation product is butyric acid. The genomic DNA G + C content of the type strain is 41.89 mol%. The type strain HA0063^T^ (=CGMCC 1.48055^T^) was isolated from the feces of rheumatoid arthritis patients.
*Lachnospira flagellata* sp. nov.	fla.gel.la'ta. L. fem. n. *flagellum*, a whip; L. fem. adj. suff. *‐ata*, suffix denoting provided with; L. fem. part. adj. *flagellata*, flagellated, denoting the type strain has flagellum.	Cells are rod‐shaped with rounded ends, with flagella, motile. Growth to stable phase occurs after 24 h incubation in mGAM medium at 37°C, pH = 7.2. No significant fermentation products detected. The genomic DNA G + C content of the type strain is 36.07 mol%. The type strain HA1242^T^ (=CGMCC 1.48424^T^ = KCTC 25682^T^) was isolated from the feces of rheumatoid arthritis patients.
*Lachnospira mobilis* sp. nov.	mo'bi.lis. L. fem. adj. *mobilis*, mobile, denoting the type strain is motile.	Cells are rod‐shaped with square ends, with flagella, motile. Growth to stable phase occurs after 24 h incubation in mGAM medium at 37°C, pH = 7.2. No significant fermentation products detected. The genomic DNA G + C content of the type strain is 36.74 mol%. The type strain HA0633^T^ (=CGMCC 1.48327^T^ = KCTC 25680^T^) was isolated from the feces of rheumatoid arthritis patients.
*Lachnospira hominis* sp. nov.	ho'mi.nis. L. gen. fem. n. *hominis*, of a human being, referring to the human gut habitat.	Cells are rod‐shaped with rounded ends, with flagella, motile. Growth to stable phase occurs after 24 h incubation in mGAM medium at 37°C, pH = 7.2. The main fermentation products are small amount of acetic, butyric, isovaleric and valeric acid. The genomic DNA G + C content of the type strain is 41.53 mol%. The type strain HA1498^T^ (=CGMCC 1.48463^T^ = KCTC 25786^T^) was isolated from the feces of rheumatoid arthritis patients.
*Lachnospira acetigenes* sp. nov.	a.ce.ti'ge.nes. L. fem. n. *acetum*, vinegar; Gr. ind. v. *gennaô*, to produce; N.L. part. adj. *acetigenes*, acetate‐producing.	Cells are rod‐shaped with rounded ends, with flagella, motile. Growth to stable phase occurs after 24 h incubation in mGAM medium at 37°C, pH = 7.2. The main fermentation product is small amount of acetic acid. The genomic DNA G + C content of the type strain is 36.85 mol%. The type strain HA2201^T^ (=CGMCC 1.48531^T^) was isolated from the feces of rheumatoid arthritis patients.
*Lactonifactor hominis* sp. nov.	ho'mi.nis. L. gen. masc. n. *hominis*, of a human being, indicating that the type strain was isolated from a human.	Cells are oval‐shaped, without flagella and nonmotile. Growth to stable phase occurs after 48 h incubation in mGAM medium at 37°C, pH = 7.2. No significant fermentation products detected. The genomic DNA G + C content of the type strain is 45.6 mol%. The type strain HA0443^T^ (=CGMCC 1.48216^T^ = KCTC 25744^T^) was isolated from the feces of rheumatoid arthritis patients.
*Mediterraneibacter tenuis* sp. nov.	te'nu.is. L. masc. adj. *tenuis*, slender, indicating the shape of the type strain.	Cells are rod‐shaped with tapered ends, without flagella and nonmotile. Growth to stable phase occurs after 48 h incubation in mGAM medium at 37°C, pH = 7.2. The main fermentation product is butyric acid. The genomic DNA G + C content of the type strain is 48.66 mol%. The type strain HA0437^T^ (=CGMCC 1.48201^T^) was isolated from the feces of rheumatoid arthritis patients.
*Waltera fermentans* sp. nov.	fer.men'tans. L. fem. part. adj. *fermentans*, fermenting, indicating that the type strain is a fermentative bacterium.	Cells are rod‐shaped with rounded ends, with flagella, motile. Growth to stable phase occurs after 48 h incubation in mGAM medium at 37°C, pH = 7.2. The main fermentation products are acetic and butyric acid, and small amount of isovaleric and valeric acid can also be produced. The genomic DNA G + C content of the type strain is 44.03 mol%. The type strain HA1509^T^ (=CGMCC 1.17997^T^ = KCTC 25717^T^) was isolated from the feces of rheumatoid arthritis patients.
*Negativibacillus intestinalis* sp. nov.	in.tes.ti.na'lis. N.L. masc. adj. *intestinalis*, of the gut, indicating that the type strain was isolated from the gut.	Cells are rod‐shaped with rounded ends, without flagella and nonmotile. Growth to stable phase occurs after 48 h incubation in mGAM medium at 37°C, pH = 7.2. No significant fermentation products detected. The genomic DNA G + C content of the type strain is 51.72 mol%. The type strain HA0568^T^ (=CGMCC 1.48289^T^ = KCTC 25700^T^) was isolated from the feces of rheumatoid arthritis patients.
*Pseudoflavonifractor intestinalis* sp. nov.	in.tes.ti.na'lis. N.L. masc. adj. *intestinalis*, pertaining to the intestine, denoting the type strain was isolated from the intestine.	Cells are long rod‐shaped with spiky ends, without flagella and nonmotile. Growth to stable phase occurs after 48 h incubation in mGAM medium at 37°C, pH = 7.2. The main fermentation product is butyric acid. The genomic DNA G + C content of the type strain is 56.3 mol%. The type strain HA1510^T^ (=CGMCC 1.17998^T^ = KCTC 25754^T^) was isolated from the feces of rheumatoid arthritis patients.
*Vescimonas butyriciproducens* sp. nov.	bu.ty.ri.ci.pro.du'cens. N.L. fem. n. *acidum butyricum*, butyric acid; L. pres. part. *producens*, producing; N.L. part. adj. *butyriciproducens*, producing butyric acid, denoting the type strain produces butyric acid.	Cells are rod‐shaped with rounded ends, without flagella and nonmotile. Growth to stable phase occurs after 72 h incubation in mGAM medium at 37°C, pH = 7.2. The main fermentation product is small amount of butyric acid. The genomic DNA G + C content of the type strain is 56.45 mol%. The type strain HA1201^T^ (=CGMCC 1.48383^T^ = KCTC 25704^T^) was isolated from the feces of rheumatoid arthritis patients.
*Vescimonas immobilis* sp. nov.	im.mo'bi.lis. L. fem. adj. *immobilis*, motionless, denoting the type strain in nonmotile.	Cells are long rod‐shaped, without flagella and nonmotile. Growth to stable phase occurs after 48 h incubation in mGAM medium at 37°C, pH = 7.2. No significant fermentation products detected. The genomic DNA G + C content of the type strain is 58.27 mol%. The type strain HA0567^T^ (=CGMCC 1.48288^T^) was isolated from the feces of rheumatoid arthritis patients.
*Peptoniphilus hominis* sp. nov.	ho'mi. nis. L. gen. masc. n. *hominis*, of a human being, indicating that the type strain was isolated from a human.	Cells are oval‐shaped, without flagella and nonmotile. Growth to stable phase occurs after 48 h incubation in mGAM medium at 37°C, pH = 7.2. The main fermentation product is isovaleric acid, and small amount of isobutyric and butyric acid can also be produced. The genomic DNA G + C content of the type strain is 49.72 mol%. The type strain HA1503^T^ (=CGMCC 1.48468^T^ = KCTC 25716^T^) was isolated from the feces of rheumatoid arthritis patients.
*Butyricimonas recta* sp. nov.	rec'ta. L. fem. part. adj. *recta*, straight, indicating that the type strain is straight rod‐shaped.	Cells are rod‐shaped with square ends, without flagella and nonmotile. Growth to stable phase occurs after 48 h incubation in mGAM medium at 37°C, pH = 7.2. The main fermentation products are small amount of propionic, isobutyric, butyric and isovaleric acid. The genomic DNA G + C content of the type strain is 62.05 mol%. The type strain HA1200^T^ (=CGMCC 1.48382^T^ = KCTC 25703^T^) was isolated from the feces of rheumatoid arthritis patients.
*Parabacteroides propionicigenes* sp. nov.	pro.pi.o.ni.ci'ge.nes. N.L. neut. n. *acidum propionicum*, propionic acid; Gr. suff. ‐*genes*, producing; from Gr. ind. v. *gennaô*, to produce; N.L. part. adj. *propionicigenes*, propionic acid producing.	Cells are rod‐shaped with oval ends, without flagella and nonmotile. Growth to stable phase occurs after 24 h incubation in mGAM medium at 37°C, pH = 7.2. The main fermentation products are acetic, propionic, isobutyric, and isovaleric acid, and small amount of butyric, and valeric acid can also be produced. The genomic DNA G + C content of the type strain is 43.11 mol%. The type strain HA3406^T^ (=CGMCC 1.48595^T^ = KCTC 25789^T^) was isolated from the feces of rheumatoid arthritis patients.
*Pyramidobacter arthritidis* sp. nov.	ar.thri'ti.dis. Gr. fem. n. *arthron*, joint; N.L. fem. n. suff. ‐itis, inflammation; N.L. gen. fem. n. arthritidis, of arthritis, denoting the type strain was isolated from the feces of rheumatoid arthritis patients.	Cells are fusiform or short rod‐shaped with rounded ends, without flagella and nonmotile. Growth to stable phase occurs after 48 h incubation in mGAM medium at 37°C, pH = 7.2. The main fermentation products, are small amount of isobutyric and isovaleric acid. The genomic DNA G + C content of the type strain is 59.84 mol%. The type strain HA0566^T^ (=CGMCC 1.48287^T^ = KCTC 25698^T^) was isolated from the feces of rheumatoid arthritis patients.

We compared the newly established RAGMB with four previously reported healthy human gut microbial biobanks, that is, hGMB [25], CGR [22], BIO‐ML [21] and HBC [24]. These four biobanks collected a total of 833 nonredundant culturable bacterial species. The RAGMB provided 121 unique gut microbial species that were not included in the above four biobanks (Figure 1D). Particularly, RAGMB provided 8 strains (HA0508, HA0551, HA0566^T^, HA0738, HA1485, HA2243, HA2245, WZ17) of the phylum *Synergistota*. These eight strains represented four species (*Cloacibacillus porcorum*, *Cloacibacillus evryensis*, *Pyramidobacter piscolens* and *Pyramidobacter arthritidis*), which were the first time being included in the human gut microbial biobanks.

### RAGMB covers both high and low‐abundant bacterial taxa from RA patients

**Figure 2:**
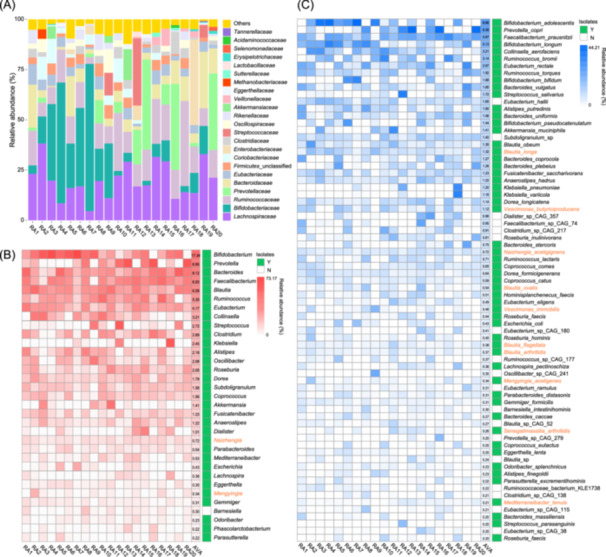
Metagenomic profiling of 20 rheumatoid arthritis (RA) fecal samples and coverages at genus and species levels by RAGMB of the fecal microbiomes. (A) shows the top 10 families of the 20 RA fecal samples. (B and C) show the relative abundances (depth of colors) and the coverages at genus (B) and species (C) levels of the RA gut microbiomes by RAGMB members. Only the members with an average relative abundance (R.A.) >0.2% in the 20 RA samples are shown in (B) and species (C), and green marked on the right side indicates species and genera preserved in RAGMB. Orange represents novel genera and species isolated in this work.

We extracted and sequenced the metagenomes of 20 RA fecal samples. Totally, we obtained 248.39 Gb raw data, with an average number of raw reads of 82,797,006 per sample (Table S9). DNA sequences were quality‐controlled and binned, and MAGs were assembled and annotated. Taxonomic annotation identified seven bacterial phyla and one archaeal phylum. The phylum *Bacillota* accounted for 52.3% of the total reads and was the highest, followed by phyla *Bacteroidota*, *Actinomycetota*, and *Pseudomonadota*. The above four phyla accounted for 98.1% of the total reads. *Verrucomicrobiota*, *Euryarchaeota*, *Synergistota*, and *Lentisphaerota* were also detected with very low abundances (<2% in total). Of note, we cultivated *Fusobacteriota* species but it was not detected in the metagenome sequences. At the family level, a total of 58 families were annotated, and the 10 top abundant families of each sample are shown in Figure 2A. Among the 10 top families, *Lachnospiraceae*, *Ruminococcaceae*, *Bacteroidaceae* and *Eubacteriaceae* occurred in all RA samples, although their abundances showed variations in different samples. The families *Bifidobacteriaceae* and *Prevotellaceae* occurred at high abundances in some samples but were depleted in other samples. At the genus and species levels, a total of 132 genera and 480 species were detected. Of those taxa, there were 81 genera (61.4%) and 135 species (28.1%) could be matched with the species preserved in RAGMB. Surprisingly, there were 145 species (51.8%) preserved in RAGMB but not detected with the metagenomic method.

To evaluate the representativeness of RAGMB, we firstly ranked the taxa (genus and species) according to their average relative abundances in RA samples, and defined that those taxa with average relative abundances ≥0.2% and ≥1% as medium‐above and highly abundant taxa, respectively. Then, we calculated the coverages of the RA fecal metagenomes by taxa in RAGMB. At genus level, there were 33 medium‐above and 21 highly abundant genera identified in the 20 RA fecal samples. The RAGMB covered 96.9% (32/33) of the medium‐above and 100% (21/21) of the high abundant genera (Figure 2B). At species level, there were 59 medium‐above and 25 highly abundant species identified in the 20 RA fecal microbiomes. The RAGMB covered 93.2% (55/59) of the medium‐above and 96.2% (25/26) of the highly abundant species (Figure 2C). In addition, the RAGMB covered also a large ratio of low abundant taxa (average relative abundances 0%–0.2%). There were 37.2% and 48.5% of RAGMB species and genera, respectively, that matched the low‐abundant taxa in the 20 RA fecal microbiomes. We observed that 145 species were exclusive with the RAGMB but not detected with metagenomes, suggesting that the cultivation and culture‐independent metagenomic methods are complementary to each other. Furthermore, the seven improved cultivation methods generated marginally but clearly higher proportion (67.4%, 58/86) of low abundant species that the 11 traditional cultivation methods (59.4%, 61/101), which indicated that there is still room for improvement and optimization of gut microbe cultivation in future. The above results suggest that the RA gut microbial isolation strategy not only resulted in high coverage of bacteria with medium‐above and high abundance but also recovered low‐abundant bacterial species.

### Core gut microbial species and their correlation with RA clinically used indices

**Figure 3:**
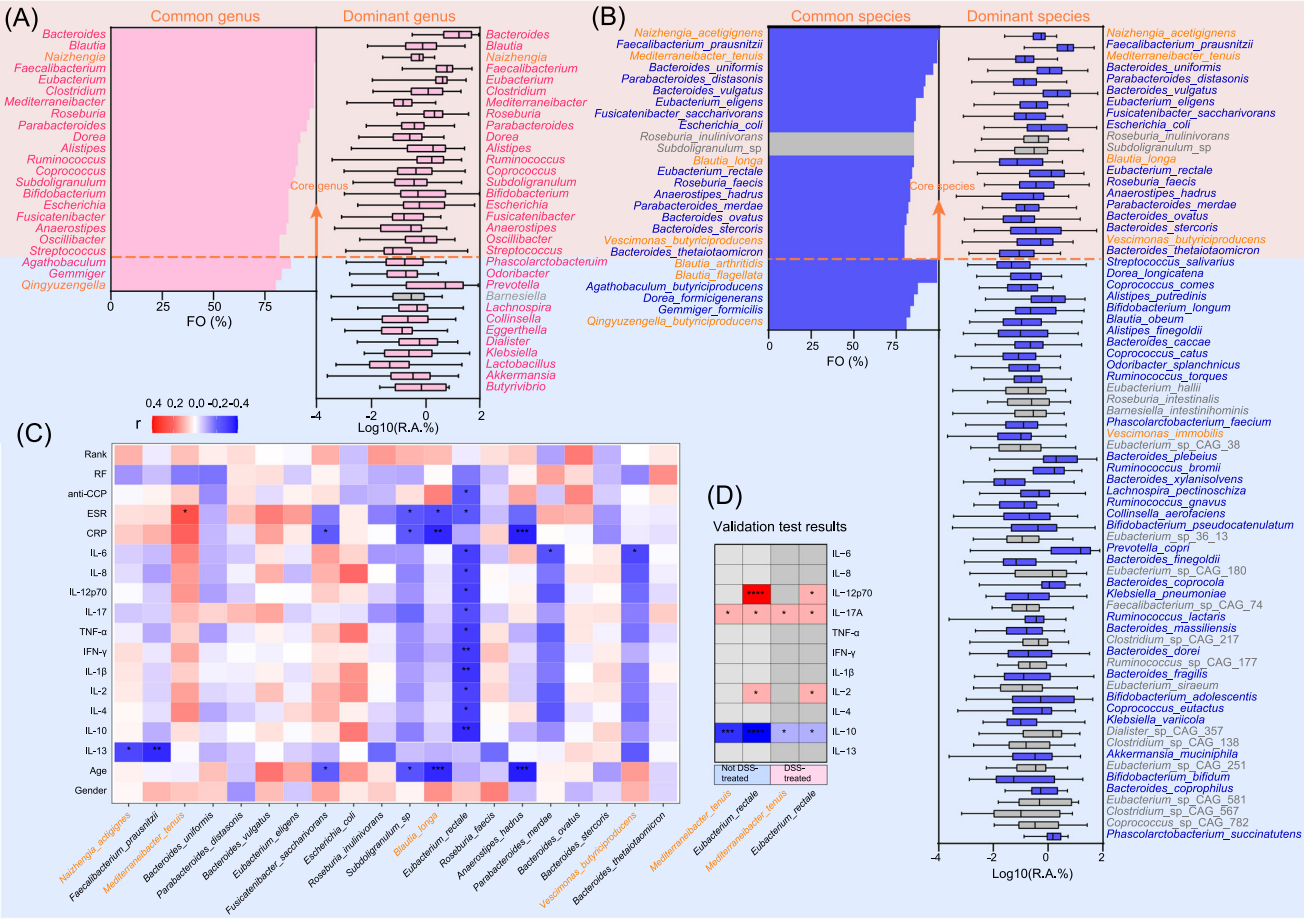
Rheumatoid arthritis (RA) core microbiomes at genus and species levels, correlations with RA clinical indices and experimental validation of the correlation between RA core microbial species and clinical diagnosis indices. (A and B), common species/genera are defined as their mean prevalence (FO) > 80%, and Dominant genera/species are defined as their mean relative abundances (R.A.) > 0.2% [Log_10_ (R.A.(%)) > −0.70]. The orange background in panels (A) and (B) highlights core genera/species that satisfy both dominant and common genera/species, and those taxa together are called RA core microbiomes. The bars in panels (A) and (B) show mean FO (%), while the box‐and‐whisker plots show Log_10_ of mean R.A. (%), centre line: median, box borders: quartiles, whiskers: min to max. (C) red represents positive correlation, blue represents negative correlation, and white represents no correlation. (D) red, blue and gray represent significant increase, significant decrease, and no significance, respectively, compared to the PBS‐gavage group. Orange represents novel genera and species isolated in this work; gray represents not isolated. The symbols ****, ***, **, and * represent *p*‐values < 0.0001, 0.001, 0.01, and 0.05 respectively.

A core microbiome is considered as stable and consistent components shared among two or more samples from hosts (such as RA patients) or environments, and is usually measured as microbial taxa [37, 38]. To explore and define the core gut microbiome of RA, we expanded our 20 RA metadata set by the addition of the metagenomic data from Jinan RA cohort (*n* = 76). The expanded metagenomic data [*n* = 96 (20 + 76)] were annotated and used for the extraction of core taxa of RA gut microbiomes. The results showed that 182 genera and 647 species were annotated in the RA metagenome datasets (Table S10), of which 88 genera (48.6%) and 157 species (24.2%) were covered by RAGMB. At the DNA sequence level, the RAGMB genomes accounted for 77.4% of the expanded RA cohort metagenomes. This result suggests that RAGMB from the 20 RA samples is highly representative of gut microbes even from larger RA cohorts. In this communication, we defined the taxa having mean relative abundance ≥0.2% and mean prevalence ≥80% as dominant and common taxa, respectively, and taxa that meet both requirements were defined as core taxa. There were 32 dominant, 23 common and 20 core genera identified from the expanded RA metagenome data. The five top core genera were *Bacteroides*, *Faecalibacterium*, *Bifidobacterium*, *Eubacterium* and *Escherichia* (Figure 3A). The RAGMB covered all 20 core genera (100%). At species level, there were 26 common, 71 dominant, and 20 core species identified from the expanded RA metagenome data. The five top core species were *Faecalibacterium prausnitzii*, *Phocaeicola vulgatus* (*Bacteroides vulgatus*), *Bacteroides stercoris*, *Escherichia coli* and *Bacteroides uniformis*. The RAGMB covered 18 of the 20 core species (90%), including four newly nominated species *Naizhengia acetigignens*, *Mediterraneibacter tenuis*, *Blautia longa*, and *Vescimonas butyriciproducens* (Figure 3B).

To discover the correlation of core microbial species to clinical indices, we applied Spearman correlation analysis for the potential relationship between the core species and the RA clinical and prognosis indices. The results showed that *Mediterraneibacter tenuis*, *Phocaeicola vulgatus*, *Bacteroides ovatus* and *Escherichia coli* were positively correlated with erythrocyte sedimentation rate (ESR), C‐reactive protein (CRP), IL‐8, and TNF‐α. But most of the core microbial species were negatively correlated to RA clinically used diagnosis indices (Figure 3C). It was very impressive that *Eubacterium rectale* showed statistically and significantly negative correlations with clinical indices including anti‐cyclic citrullinated peptide antibodies (anti‐CCP), ESR, and cytokines such as TNF‐α, IL‐1β, IL‐6, and the anti‐inflammatory IL‐10. The core species *Anaerostipes hadrus*, *Blautia longa*, *Faecalibacterium prausnitzii*, *Fusicatenibacter sacchanivorans*, *Parabacteroides merdae*, *Viscinimonas butyriproducens*, and uncultured *Subdoligranulum*_sp showed negative correlations with ESR, CRP, IL‐6 or IL‐13. It is noteworthy that several of the above core species were also correlated to RA patient's ages, for example, *Anaerostipes hadrus*, *Blautia longa*, *Fusicatenibacter sacchanivorans*, and uncultured *Subdoligranulum*_sp were negatively but *Phocaeicola vulgatus* and *Escherichia coli* were positively correlated to ages (Figure 3C).

### The *M. tenuis* and *E. rectale* exacerbated inflammatory responses in mice

**Figure 4:**
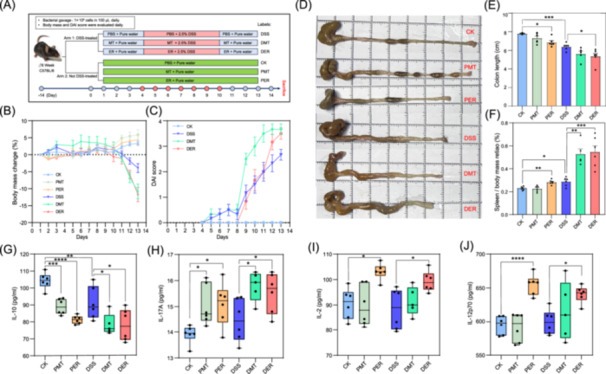
The rheumatoid arthritis (RA) core species *M. tenuis* and *E. rectale* promote host inflammatory responses in the mice model. (A) experimental process diagram. (B and C) show the body mass change and disease activity index (DAI) scores of the mice during the experiment, whiskers: SEM. (D) displays photographs of the colon from different groups at the endpoint. (E and F) present the colon length and spleen/body weight ratio at the endpoint, column top borders: mean, whiskers: SEM. (G–J) show plasma levels of IL‐10 (G), IL‐17A (H), IL‐2 (I), and IL‐12p70 (J) from mice at the endpoint, center line: median, box borders: quartiles, whiskers: min to max. CK group: Pure water + PBS; PMT: Pure water + *M. tenuis*; PER: Pure water + *E. rectale*; DSS: DSS water + PBS; DMT: DSS water + *M. tenuis*; DER: DSS water + *E. rectale*. *p < 0.05, **p < 0.01, ***p < 0.001, and ****p < 0.0001.

To validate the impact of core microbial species on RA, we selected *M. tenuis* and *E. rectale*, two species from the RA core microbiome defined in this study and showed extensive correlation to inflammatory and immune responses of RA patients, for intragastric administration in mice. Briefly, the experiments had two arms (for detail, see Figure 4A), one with DSS‐treatment (labeled as DSS, DMT, and DER), and the other without DSS‐treatment (labeled as CK, PMT, and PER). The gavages of *M. tenuis* and *E. rectale* significantly aggravated body mass losses in the groups treated with DSS (DSS, DMT, and DER) after day 12, but slightly increased body mass gains in the groups without DSS treatment (CK, PMT, and PER) (Figure 4B). The DAI (Disease Activity Index) scores of not DSS‐treated groups were close, but were very different of DSS‐treated groups. The gavages of *M. tenuis* and *E. rectale* significantly increased DAI scores (Figure 4C). At the endpoint, all groups (DSS treated or not treated) administrated with *E. rectale* showed significant reduction of colon length (Figure 4D,E) and significant increase of spleen weight (Figure 4F). The effects of *M. tenuis* gavages were a little mild on colon length but with observable reduction in all groups (DSS treated or not treated) (Figure 4D,E), but was significantly increased spleen weight in the DSS‐treated group (Figure 4F). These results suggested that both *M. tenuis* and *E. rectale* exacerbated the development of colitis and altered intestinal immune homeostasis in mice. To validate the associations of clinically used indices with core microbial species (Figure 3C), we determined the inflammatory and immune indices (listed in Figure 3C) in the plasma from mice that were administrated with *M. tenuis* and *E. rectale*. The results are presented in Figure 3D. We observed a significant reduction in IL‐10 levels and an increase in IL‐17A levels of plasma in the *M. tenuis* and *E. rectale* gavage groups, regardless of DSS‐treated or not treated (Figure 4G,H), which clearly indicated that pro‐inflammatory signals [39, 40] were affected. In addition, the administration of *E. rectale* promoted the increases of IL‐2 and IL‐12p70 (Figure 4I,J).

## DISCUSSION

So far as we know, the previously established gut microbial biobanks are mainly concern healthy populations [21, 22, 24, 25], and targeted cultivation and collection of gut microbial strains from patients are very limited. The goal of this study was to cultivate gut microbial strains from RA patients, thus making those strains available for fundamental and clinical research. Consequently, the RAGMB, the first representative biobank of the gut microbiota from RA patients, has been established. To achieve this goal, we modified culture media (using xylan as the sole carbon source) and sample pretreatment, and adapted cultivation protocol for the collection of RA‐enriched gut microbes such as *Pvt. copri* and *Fusobacterium nucleatum* [8, 41, 42]. Compared to the emerging new technology for the cultivation of these bacteria [43, 44], the newly developed cultivation protocol was easy to follow and was effective in growing not only *Pvt. copri* and *Fusobacteriota* but also other species from low abundance phyla such as *Synergistetes* and *Verrucomicrobiota*. Using this strategy, we isolated and preserved 601 bacterial strains that represent 280 species from 146 genera, including 43 novel species and 11 novel genera. The diverse strains and species of the RAGMB provide valuable bioresources for medical and fundamental research especially for discovering RA etiology and developing new diagnoses as well as treatment in the future. We observed that the 43 novel species were widely distributed among RA patients and healthy people, and many of them showed different abundances between RA patients and healthy peoples. These novel species certainly added value to RAGMB and enlarged the bacterial spectrum to find RA pathogens and/or develop new probiotics for RA medication. Currently, experiments are in progress on the evaluation of a couple of these novel species in collaboration with clinical research teams.

In this study, we proposed and defined an RA‐core gut microbiome (defined dominant as having mean relative abundance ≥0.2% and common as having mean prevalence ≥80%) for RA patients. Core gut microbial species are highly abundant and widely occurring taxa in host guts [25]. These microbes may create a pro‐inflammatory and pathogenic microenvironment that facilitates the presence and activity of other pathogenic species [45]. The core gut microbial species may offer an ecological context for more in‐depth exploration of pathogenic and therapeutic microbiome research, potentially serving as a crucial foundation for studies involving gnotobiotic animal models. The RA‐core gut microbiome from this current study is composed of 20 species, and covers the enriched and potentially pathogenic species of previously reported species such as *E. coli* [46, 47], as well the depleted and potentially probiotic species such as *F. prausnitzii* [48, 49]. The identified core microbial species are potential new targets for new biomedical practices. Nevertheless, the defined RA‐core microbial species might be expanded in the future studies. *Pvt. copri* ranked the dominant species from RA samples in our study, and this species was repeatedly reported as being RA pathogenic [5, 6, 11, 12, 13]. We isolated and cultivated 15 *Pvt. copri* strains from the 20 RA fecal samples, this high recovery frequency supports that this species played an important role in RA etiology and progression. However, this species was not covered by the RA‐core microbiome. It is noteworthy that *Pvt. copri* are diverse at strain level, and many strains were also detected and cultivated from healthy people [50, 51].

In this study, we observed that the core microbial species were correlated to clinically used diagnosis indices including anti‐CCP, CRP, ESR, and inflammatory cytokines, which suggested that these core microbial species might affect host inflammation and immunity [52]. Remarkably, *M. tenuis* and *E. rectale* showed correlations with statistical significance. Thus, the core microbial species *M. tenuis* and *E. rectale* were applied for validation of the correlations in our study with mouse model. Our results showed that both species significantly increased IL‐17A while decreased IL‐10 levels of plasma. IL‐17A is a major pro‐inflammatory cytokine produced by Th17 cells, and plays a key role in the development of several autoimmune diseases [53]. Th17 cells have been reported to preferentially accumulate in the gut and bridged the microbe‐host immune system interactions [53]. In addition, we found that *E. rectale* also significantly increased IL‐2 and IL‐12p70 levels of plasma, indicating its potential ability to enhance Th1 cell responses [54, 55]. Th1 cells play a key pro‐inflammatory role in RA progression [54, 56]. In summary, *M. tenuis* consistently showed strong pro‐inflammatory effect and elevated IL‐17A levels, but *E. rectale* showed discrepancy in statistic and experimentally validated correlations: The statistics suggested that *E. rectale* was negatively correlated to RA and was putatively anti‐inflammatory, while the experimental results demonstrated that *E. rectale* promoted inflammation and significantly reduced IL‐10, IL‐17, IL‐2 and IL‐12p70. We noticed that previous reports on the pro‐ and anti‐inflammatory potentials of *E. rectale* were controversial and the effects might be strain‐specific [57, 58]. Given the frequent emergence of microbial strain diversity in guts and strain‐specific effect on host health [34, 59], our study underlines the importance of gut microbial biobanks of cultured strains from healthy and diseased individuals.

## CONCLUSIONS

In this study, we obtained 3200 bacterial isolates from fecal samples of 20 RA patients, through the extensive bacterial cultivation methods (seven improved and 11 traditional). We selected 601 bacterial strains and they were deposited in the RAGMB, representing 280 species (including 43 novel species characterized and nominated) of seven bacterial phyla. The RAGMB that covered 93.2% at species level of medium‐ and high‐abundant gut microbes with RA patients. We also defined the core microbiome of RA patients and further correlated core microbial species with clinically used indices. The core microbial species *M. tenuis* and *E. rectale* were experimentally demonstrated being pro‐inflammatory with both DSS‐treated and not treated mice models.

## METHODS

The entire project was approved by the Medical Ethics Committee of the Affiliated Hospital of Qingdao University, with the ethical approval number QYFY WZLL 28052. Detailed descriptions of feces and plasma samplings, bacterial isolation and cultivation, 16S rRNA gene sequencing, bacterial identification and taxonomy, bacterial genome and fecal metagenome sequencing, bacterial strain preservation, treatments of DSS‐treated mice model and data statistics and analysis are available in the Supplementary Methods section. Information of RA patients is presented in Table S3.

## AUTHOR CONTRIBUTIONS

**Hao‐Jie Huang**: Conceptualization; methodology; validation; formal analysis; writing—original draft; resources; data curation; visualization; software; investigation. **Chang Liu**: Validation; writing—review and editing. **Xin‐Wei Sun**: Validation; methodology; writing—original draft. **Rui‐Qi Wei**: Validation. **Ling‐Wei Liu**: Resources. **Hao‐Yu Chen**: Validation. **Rashidin Abdugheni**: Resources; validation. **Chang‐Yu Wang**: Validation. **Xiao‐Meng Wang**: Validation. **He Jiang**: Resources. **Han‐Yu Niu**: Validation. **Li‐Juan Feng**: Validation. **Jia‐Hui He**: Validation. **Yu Jiang**: Methodology; validation. **Yan Zhao**: Resources. **Yu‐Lin Wang**: Software. **Qiang Shu**: Methodology; investigation. **Ming‐Xia Bi**: Resources. **Lei Zhang**: Resources. **Bin Liu**: Resources. **Shuang‐Jiang Liu**: Methodology; writing—review and editing; project administration; resources; funding acquisition; formal analysis; supervision.

## CONFLICT OF INTEREST STATEMENT

The authors declare no conflict of interest.

## ETHICS STATEMENT

The fecal sample collection was approved by the Medical Ethics Committee of the Affiliated Hospital of Qingdao University, with the ethical approval number QYFY WZLL 28052. And all fecal sample donors provided written informed consent before participation in the study. The animal studies were conducted in accordance with the Helsinki Declaration and were approved by the Ethics Committee for the Care and Use of Laboratory Animals of Shandong University (No. SYDWLL‐2022‐086).

## Supporting information

Supplementary Methods.
Supplementary Taxon.

**Table S1:** mX Agar Medium Recipe.
**Table S2:** Pretreatments Used for Isolation and Cultivation of Gut Microbes from RA Patients.
**Table S3:** Information on RA Patients.
**Table S4:** Strain Information in RAGMB.
**Table S5:** Genome Information on 43 Novel Species in RAGMB.
**Table S6:** Short‐Chain Fatty Acid Production of Novel Species in RAGMB.
**Table S7:** Annotation Results of Novel Species from RAGMB in HC and RA Metagenomic Datasets.
**Table S8:** Distribution Significance of Novel Species in RA and HC Metagenomic Datasets.
**Table S9:** QC Statistics of Metagenomic Sequencing Data from 20 RA Samples.
**Table S10:** Annotation Results of RA Expanded Metagenomic Datasets.
**Table S11:** Information on Healthy Human Fecal Metagenomic Samples.

## Data Availability

The datasets generated and analyzed in this study are available as the following: The descriptive information and data of 601 isolates of RAGMB, representing 280 different species from seven phyla, were available at the RAGMB homepage (https://www.nmdc.cn/ragmb/). The 16S rRNA gene sequences of the 601 were deposited in China National Microbiology Data Center (NMDC) with accession numbers NMDCN0001EL0 ‐ NMDCN0001F7O. 43 novel species assembled genomes in this study were available at NMDC under Project NMDC10018517 (https://nmdc.cn/resource/genomics/project/detail/NMDC10018517). Fecal metagenomics data of 20 RA patients in this study were available at NMDC under Project NMDC10018632 (https://nmdc.cn/resource/genomics/project/detail/NMDC10018632). The data and scripts used are saved in GitHub: https://github.com/hhj00123/2024_RAGMB. Supplementary materials (methods, tables, graphical abstract, slides, videos, Chinese translated version, and update materials) may be found in the online DOI or iMeta Science http://www.imeta.science/.
