# Supplementary material for: The rheumatoid arthritis gut microbial biobank reveals core microbial species that associate and effect on host inflammation and autoimmune responses
Source: Imeta. 2024 Oct 3;3(5):e242. doi: 10.1002/imt2.242 (PMC11487554; doi:10.1002/imt2.242)
Supplement: Supplementary file 1 — Supplementary Methods. Supplementary Taxon. [file IMT2-3-e242-s001.docx]

**Supporting information to**

**The rheumatoid arthritis gut microbial biobank reveals core microbial species that associate and effect on host inflammation and autoimmune responses**

**Running title:** The core gut microbiome/species from RAGMB effect on host inflammatory responses

Hao-Jie Huang^1^, Chang Liu^1^, Xin-Wei Sun^1^, Rui-Qi Wei^1^, Ling-Wei Liu^2^, Hao-Yu Chen^1^, Rashidin Abdugheni^3^, Chang-Yu Wang^4^, Xiao-Meng Wang^1^, He Jiang^1^, Han-Yu Niu^5^, Li-Juan Feng^1^, Jia-Hui He^5^, Yu Jiang^1^, Yan Zhao^6^, Yu-Lin Wang^1^, Qiang Shu^7^, Ming-Xia Bi^1^, Lei Zhang^1,8^*, Bin Liu^2^*, Shuang-Jiang Liu^1,9^*

^1^State Key Laboratory of Microbial Technology, Shandong University, Qingdao 266237, China.

^2^Department of Rheumatology, The Affiliated Hospital of Qingdao University, Qingdao 266000, China.

^3^State Key Laboratory of Desert and Oasis Ecology, Key Laboratory of Ecological Safety and Sustainable Development in Arid Lands, Xinjiang Institute of Ecology and Geography, Chinese Academy of Sciences, Ürűmqi 830011, China.

^4^University of Science and Technology of China, Hefei 230026, China

^5^College of Veterinary Medicine, Shanxi Agricultural University, Taigu 030801, China.

^6^Biomedical Sciences College & Shandong Medicinal Biotechnology Centre, Shandong First Medical University & Shandong Academy of Medical Sciences, Jinan 250012, China.

^7^Department of Rheumatology, Qilu Hospital, Cheeloo College of Medicine, Shandong University, Jinan 250012, China.

^8^Microbiome-X, School of Public Health, Cheeloo College of Medicine, Shandong University, Jinan 250012, China.

^9^State Key Laboratory of Microbial Resources, Institute of Microbiology, Chinese Academy of Sciences, Beijing 100101, China.

*Corresponding authors: [liusj@sdu.edu.cn](mailto:liusj@sdu.edu.cn) (Shuang-Jiang Liu), [binliu72314@163.com](mailto:binliu72314@163.com) (Bin Liu), [zhanglei7@sdu.edu.cn](mailto:zhanglei7@sdu.edu.cn) (Lei Zhang)

**Supplementary Methods**

**Sample collection and treatment**

The entire project was approved by the Medical Ethics Committee of the Affiliated Hospital of Qingdao University, with the ethical approval number QYFY WZLL 28052. Feces and plasma samples from RA patients were collected at the Affiliated Hospital of Qingdao University, in a total of 6 times collections with 3-4 samples each time. Before collection, the past two months' history of antibiotic treatment, dietary preferences, and BMI were obtained from each prospective donor candidate, along with a recording of RA activity score (DAS-28) and other information. A total of 20 qualified feces samples were collected, including 8 from newly diagnosed RA patients. Each feces sample was divided into two portions. One was immediately stored at -20°C for DNA extraction, another was stored in a round bottom vertical anaerobic culture bag (Hopebio, CHN) containing AneroPack-Anaero (MGC, JPN) at room temperature, which was transferred to an anaerobic workstation (COY, USA) within 4 hours for gut microbe isolation. The gas flow composition in the anaerobic workstation was 85% N_2_, 5% CO_2_, and 10% H_2_.

**Bacterial isolation, cultivation, and 16S rRNA gene sequencing**

Fresh feces samples were suspended in PBS or a certain concentration of ethanol. The suspension was filtered through a cell filter to remove large insoluble particles in the suspension, and then serially diluted from 10^-1^ to 10^-8^. Subsequently, 100 μL of each dilution was spread on different agar plates or added to enrichment media at a ratio of 1:100 for 10^-1^ suspension, followed by aerobic or anaerobic incubation at 37°C. Eighteen different treatments and culture conditions were used for bacterial isolation (Table S2). Colony isolation and identification were performed as described in our previous study with modifications [1]: single colonies were picked from agar plates cultured for 2-30 days and streaked on new agar plates with corresponding culture media. The streaked isolates were cultured at 37°C for the same number of days as the pick-up time. Then, single colonies were picked from the isolates, and 2 μL of NaOH/SDS lysis buffer (Amresco, USA) was used to lyse the bacterial pellet, followed by dilution with 98 μL of deionized water. The bacterial lysate was heated at 98°C for 30 minutes to ensure complete lysis. The fully lysed solution (2 μL) was used as a template for PCR amplification of the 16S rRNA gene using 2×Es Taq MasterMix (Dye) (CWBIO, CHN) and universal primers for the 16S rRNA gene (Primers: 27 F: 5′-AGAGTTT GATCCTGGCTCAG-3′; 1492 R: 5′-GGTTACCTTGTTACGACTT-3′). The PCR products were subjected to Sanger sequencing (Tsingke Biotechnology Co., Ltd., CHN). The sequencing results were analyzed by BLAST analysis based on the EZBioCloud and NCBI 16S ribosomal RNA sequence (last updated on 2023/08/18, sequence count: 26,876) databases to determine the classification of the strains. Based on the 16S rRNA gene sequence identity (with a threshold of 98.65% for different species), the corresponding species and potential novel species of different strains were determined. If the 16S rRNA gene sequence identity between the strain and all valid published species in the two databases was >98.65, it was considered an existing species. If the 16S rRNA gene sequence identity between the strain and all valid published species in the two databases was ≤ 98.65, it was considered a potential novel species. All strains corresponding to potential novel species would undergo polyphasic identification.

**The judgment basis of different strains and the preservation strategy**

We collated the origin, physiological and biochemical phenotypes and 16S rRNA gene sequencing results of all isolates. Isolates were considered different strains when they met any of the following standards: 1) the 16S rRNA gene similarity of two isolates was less than 99%; 2) two isolates belonged to the same species but were obtained from different volunteers' fecal samples; 3) there were noticeable physiological and biochemical distinctions between the two isolates, encompassing differences in colony morphology, divergences in maximum optical density (OD) for growth in the same liquid medium exceeding 0.5, or a period of greater than 24 hours.

All the different strains were preserved in three ways: 1) Cultures were grown on corresponding slant agar media until visible bacterial colonies appeared and then stored in a preservation room at 18°C; 2) Cultures were grown to late logarithmic phase under appropriate growth conditions. Then 0.1 g of bacterial biomass was suspended in 1 mL of 20% glycerol and stored in cryovials (Corning, USA) placed in an anaerobic workstation. The cryovials were placed in a round bottom vertical anaerobic culture bag (Hopebio, CHN) filled with AneroPack-Anaero (MGC, JPN) and stored at -80°C in a freezer. Four replicates of each strain were stored; 3) Cultures were grown under appropriate growth conditions to the late logarithmic phase. Then, 0.1 g of bacterial biomass was suspended in 200 μL of sterilized cryoprotectant solution (5% skim milk (DB, USA), 3% lactose, 2% glycerol) and freeze-dried under a nitrogen atmosphere within an anaerobic workstation. The freeze-dried bacterial biomass was stored in a round bottom vertical anaerobic culture bag (Hopebio, CHN) filled with AneroPack-Anaero (MGC, JPN) and kept at 4°C in a refrigerator. After 3 months of storage, all stored strains were resuspended and passaged. A total of 601 strains that could grow normally and stably passaged were re-preserved in RAGMB and CGMCC. Detailed information about the strains can be found at the RAGMB website (https://www.nmdc.cn/ragmb/). To comply with the rules of the International Code of Nomenclature of Prokaryotes (ICNP), 43 novel species were also preserved in a secondary IDA within RAGMB, as KCTC. Detailed information on the novel species can be found in Table 1 and the RAGMB website (https://www.nmdc.cn/ragmb/).

**Polyphasic characterization and nomenclature of novel taxa**

The description of the new taxa is based on five types of analyses, including phylogenetics, genomics, physiological characters, biochemical characters, and morphology, as detailed in the RAGMB website (https://www.nmdc.cn/ragmb/)**.** The methods followed previous studies with modifications [1, 2]: the 16S rRNA gene sequencing results were used as a basis for BLAST analysis, the gene sequences of closely related species were aligned using MEGA X [3], and constructing phylogenetic trees using the neighbor-joining method to show the phylogenetic distribution and classification relationships of each new taxon and its closely related taxa. Additionally, phylogenetic trees based on the genomes of each novel species were constructed using CVTree3 [4] with default parameters. The closely related taxa on phylogenetic and phylogenomic trees were further analysed for genomic-based analysis. Genomic-based analysis of the new taxa included calculation of average nucleotide identity (ANI), digital DNA:DNA hybridization (dDDH), and percentage of conserved proteins (POCP). ANI values and heatmaps were generated using the OrthoANI OTA software [5]. The dDDH values were calculated using the Genome-to-Genome Distance Calculator 2.1 (GGDC) [6]. ComputePOCP_v1.1 was used to calculate the POCP values [7, 8], which were used for genus-level taxonomic descriptions. The production of short-chain fatty acids by the strains was analysed using GC-MS according to previous methods [9]. The bacterial cell morphology was observed using a transmission electron microscope (TEM) FEI Tecnai G2 F20 (Thermo Scientific, USA). The bacterial colony morphology was observed using a stereomicroscope SMZ18 (Nikon, JPN) and an automated colony counter (Shineso, CHN). The bacterial motility was examined using an optical microscope Eclipse Ts2R (Nikon, JP). The criteria for the new taxa followed previous articles, with modifications [1]: 1) A taxon is defined as a novel species if it fulfills the following three criteria: (1) 16S rRNA sequence identity <98.65%; (2) dDDH value <70%; (3) ANI <95% or ANI between 95% and 96.2% but with distinct morphological and physiological characteristics from its closely related species. 2) If a novel species simultaneously exhibits (1) < 95.0% 16S rRNA gene sequence identity to any known species; (2) <50% POCP value to its closely related species; (3) significant differences in morphology and physiological characteristics from its closely related species; (4) placement in a separate branch on the phylogenetic tree, the taxon would be further defined as a new genus. The nomenclature of each new taxon follows the rules of the ICNP.

**Sequencing of gut metagenome and bacteria genomes**

Fecal samples were thawed on ice and genomic DNA was extracted using the DNeasy PowerSoil Pro Kit (Qiagen, GER) according to the manufacturer's instructions. Bacterial genomic DNA was extracted using the TIANamp Bacteria DNA Kit DP302 (TIANGEN, CHN). Log phase bacterial cultures were centrifuged to collect 0.5g /DW of bacterial cells, and DNA extraction was performed according to the manufacturer's instructions. All DNA extraction procedures were treated with RNase (YEASEN, CHN) to remove RNA contamination. DNA content was determined using a NanoDrop spectrophotometer, Qubit fluorometer (using the Quant-iTTM dsDNA BR Assay Kit), and agarose gel electrophoresis.

Metagenomic sequence and bacterial draft genomic data were generated on the Illumina HiSeq Novseq6000 platform at Guangdong Magigene Biotechnology Co., Ltd. Raw metagenomic sequencing data were processed using Trimmomatic V.0.36 (http://www.usadellab.org/cms/?page=trimmomatic) to obtain clean data. Bacterial genomic raw data were processed using the following steps, and high-quality sequences were used for downstream data analysis: (1) Removal of low-quality bases at both ends of reads (quality value <) and removal of low-length reads (default set at 50bp); (2) Deletion of reads containing a certain proportion of N bases (default set at 10bp); (3) Deletion of reads between a certain threshold (default set at 15bp) and adapters; (4) For small genomes, if the sample has host contamination, it is aligned against the host database set to filter out reads that may be due to host contamination. (5) Removal of duplicate contamination. After data filtering and quality control, the clean second-generation data were assembled de novo using the SPAdes v3.13.0 tool [10] to obtain high-quality contigs fragments.

**Human gut metagenome,** **plasma samples collection and analysis**

Apart from utilizing 20 RA patient’s gut microbiome metagenomic data from this study, RA and HC cohorts from previous studies were downloaded for analysis. Another RA cohort was derived from a published cohort from Shandong, China (RA, *n* = 76) [11]. The HC cohorts were consistent with our previous study (Table S11) [12] and included eligible raw data from Canada, the United Republic of Tanzania, Italy, China, and the United States (*n* = 1153). The isolated genomes of the novel species were incorporated into the MetaPhlAn 3 mpa_v31_CHOCOPhlAn database by customizing the database and adding the marker genes [13]. The updated database was used for species annotation, abundance and prevalence calculations in the metagenomic data. The data of plasma samples were also from previous studies [11], and used to analyse the correlation between core species and clinical indices, including anti-CCP antibodies, ESR, CRP, rheumatoid factor (RF), and inflammatory cytokines.

**Bacterial genomes analysis**

The assembled bacterial genome data was used to predict genes using Prodigal software [14]. Genomes were annotated using the eggNOG 5.0 database [15] and KEGG databases [16] to analyze gene composition, COG classification[17] and pathway mapping. All abundance and prevalence of novel species in the metagenomic samples were calculated using CoverM (version 0.6.1) (<https://github.com/wwood/CoverM>), --min-read-percent-identity 95 --min-read-aligned-percent 70.

**Bacterial diversities of different culture collections**

The method for extracting taxonomic information from large-scale culture collections of healthy human gut microorganisms were referred to our previously published work. Information extraction for strain 16S rRNA of CGR [18], BIO-ML [19], and HBC [20] were consistent with previous methods [1]. hGMB [1] strain 16S rRNA gene sequences were downloaded from the hGMB website (<http://www.cgmcc.net/english/hgmb>).

**Animals and** **treatments of mice**

All C57BL/6 J male mice (6 weeks old) were purchased from Beijing Vital River Laboratory Animal Technology Co., Ltd. (Beijing, China). The mice were housed in a specific-pathogen-free (SPF) environment under controlled conditions, a 12-h light/dark cycle, a temperature of 25 °C, and (45 ± 5) % humidity, with free access to food and water. All mice were acclimated for two weeks before the experiment.

Based on the design shown in Figure 4a, mouse body weights and disease activity index (DAI) scores were recorded daily from day 1, with DAI scores calculated as described in previous reports [21]. Each mouse was gavaged with 0.1 mL of the appropriate bacterial strain at 1 × 10^9 CFU/mL (*Mediterraneibacter tenuis* HA0437 and *Eubacterium rectale* HA1162 from this work) or PBS until day 13. On day 4, mice were given 2.5% dextran sulfate sodium salt (DSS) (molecular weight 36,000-50,000 Da, Yeasen Biotechnology Co. Ltd, Shanghai, CHN) dissolved in drinking water or pure water for 7 days at discretion, after which they were switched back to pure water until the endpoint. At the endpoint, mice were euthanised by CO_2_ anaesthesia and blood, spleen, colon and cecum were collected immediately. Spleen weight and colon length were measured. Plasma cytokines were quantified using ELISA kits (Boshen Biotechnology Co., Ltd, Jiangsu, CHN) according to the manufacturer's instructions.

The animal studies were conducted in accordance with the Helsinki Declaration and were approved by the Ethics Committee for the Care and Use of Laboratory Animals of Shandong University [SYDWLL-2022-086].

**Statistical analysis**

All statistical analyses were performed using GraphPad Prism 9.0 and R software (version 4.2.2). The gut microbiota of RA patients and healthy controls were categorised into RA and HC groups, respectively. Correlation between patient biochemical indicators and core species was assessed using Spearman's rank correlation test. A correlation was considered significant if the *r_s_* value was ≥ 0.2 or ≤ -0.2 [22], with a corresponding *p*-values < 0.05. The significance of the differences in abundance of the RAGMB novel species in the RA and HC groups was calculated using the ggpubr package and the Wilcoxon non-parametric test. The statistical significance of the normally distributed data of physiological and biochemical, were assessed by analysis of variance followed by Dunnett’s multiple comparisons test. Differences were considered significant when *p* < 0.05. **** indicates *p* < 0.0001; *** indicates *p* < 0.001; ** indicates *p* < 0.01; and * indicates *p* < 0.05. In addition to the above, GraphPad Prism version 9.0 was used for visualization of the results.

**REFERENCES**

1. Liu, Chang, Meng-Xuan Du, Rexiding Abuduaini, Hai-Ying Yu, Dan-Hua Li, Yu-Jing Wang, Nan Zhou, et al. 2021. “Enlightening the taxonomy darkness of human gut microbiomes with a cultured biobank.” *Microbiome* 9: 119. https://doi.org/10.1186/s40168-021-01064-3

2. Liu, Chang, Nan Zhou, Meng-Xuan Du, Yi-Tong Sun, Kai Wang, Yu-Jing Wang, Dan-Hua Li, et al. 2020. “The Mouse Gut Microbial Biobank expands the coverage of cultured bacteria.” *Nature Communications* 11: 79. <https://doi.org/10.1038/s41467-019-13836-5>

3. Kumar, Sudhir, Glen Stecher, Michael Li, Christina Knyaz, Koichiro Tamura. 2018. “MEGA X: Molecular Evolutionary Genetics Analysis across Computing Platforms.” *Molecular Biology and Evolution* 35: 1547-1549. <https://doi.org/10.1093/molbev/msy096>

4. Zuo, Guang-Hong, Bai-Lin Hao. 2015. “CVTree3 Web Server for Whole-genome-based and Alignment-free Prokaryotic Phylogeny and Taxonomy.” *Genomics Proteomics Bioinformatics* 13: 321-331. <https://doi.org/10.1016/j.gpb.2015.08.004>

5. Lee, Imchang, Yeong Ouk Kim, Sang-Cheol Park, Jongsik Chun. 2016. “OrthoANI: An improved algorithm and software for calculating average nucleotide identity.” *International Journal of Systematic and Evolutionary Microbiology* 66: 1100-1103. <https://doi.org/10.1099/ijsem.0.000760>

6. Meier-Kolthoff, Jan P., Alexander F Auch, Hans-Peter Klenk, Markus Göker. 2013. “Genome sequence-based species delimitation with confidence intervals and improved distance functions.” *BMC Bioinformatics* 14: 60. <https://doi.org/10.1186/1471-2105-14-60>

7. Lagkouvardos, Ilias, Rüdiger Pukall, Birte Abt, Bärbel U. Foesel, Jan P Meier-Kolthoff, Neeraj Kumar, Anne Bresciani, et al. 2016. “The Mouse Intestinal Bacterial Collection (miBC) provides host-specific insight into cultured diversity and functional potential of the gut microbiota.” *Nature Microbiology* 1: 16131. <https://doi.org/10.1038/nmicrobiol.2016.131>

8. Qin, Qi-Long, Bin-Bin Xie, Xi-Ying Zhang, Xiu-Lan Chen, Bai-Cheng Zhou, Jizhong Zhou, Aharon Oren, Yu-Zhong Zhang. 2014. “A proposed genus boundary for the prokaryotes based on genomic insights.” *Journal of Bacteriology* 196: 2210-2215. <https://doi.org/10.1128/jb.01688-14>

9. Sun, Xin-Wei, Rashidin Abdugheni, Hao-Jie Huang, Yu-Jing Wang, Min-Zhi Jiang, Chang Liu, Nan Zhou, He Jiang, Shuang-Jiang Liu. 2022. “*Bacteroides propionicigenes* sp. nov., isolated from human faeces.” *International Journal of Systematic and Evolutionary Microbiology* 72: <https://doi.org/10.1099/ijsem.0.005397>

10. Bankevich, Anton, Sergey Nurk, Dmitry Antipov, Alexey A Gurevich, Mikhail Dvorkin, Alexander S Kulikov, Valery M Lesin, et al. 2012. “SPAdes: a new genome assembly algorithm and its applications to single-cell sequencing.” *Journal of Computational Biology* 19: 455-477. <https://doi.org/10.1089/cmb.2012.0021>

11. Cheng, Mingyue, Yan Zhao, Yazhou Cui, Chaofang Zhong, Yuguo Zha, Shufeng Li, Guangxiang Cao, et al. 2022. “Stage-specific roles of microbial dysbiosis and metabolic disorders in rheumatoid arthritis.” *Annals of the Rheumatic Diseases* 81: 1669-1677. https://doi.org/10.1136/ard-2022-222871

12. Liu, Chang, Meng-Xuan Du, Li-Sheng Xie, Wen-Zhao Wang, Bao-Song Chen, Chu-Yu Yun, Xin-Wei Sun, et al. 2024. “Gut commensal *Christensenella minuta* modulates host metabolism via acylated secondary bile acids.” *Nature Microbiology* 9: 434-450. https://doi.org/10.1038/s41564-023-01570-0

13. Beghini, Francesco, Lauren J McIver, Aitor Blanco-Míguez, Leonard Dubois, Francesco Asnicar, Sagun Maharjan, et al. 2021. “Integrating taxonomic, functional, and strain-level profiling of diverse microbial communities with bioBakery 3.” *Elife* 10: e65088 <https://doi.org/10.7554/eLife.65088>

14. Hyatt, Doug, Gwo-Liang Chen, Philip F Locascio, Miriam L Land, Frank W Larimer, Loren J Hauser. 2010. “Prodigal: prokaryotic gene recognition and translation initiation site identification.” *BMC Bioinformatics* 11: 119. <https://doi.org/10.1186/1471-2105-11-119>

15. Huerta-Cepas, Jaime, Damian Szklarczyk, Davide Heller, Ana Hernández-Plaza, Sofia K Forslund, Helen Cook, Daniel R Mende, et al. 2019. “eggNOG 5.0: a hierarchical, functionally and phylogenetically annotated orthology resource based on 5090 organisms and 2502 viruses.” *Nucleic Acids Research* 47: 309-314. <https://doi.org/10.1093/nar/gky1085>

16. Kanehisa, Minoru, Miho Furumichi, Yoko Sato, Masayuki Kawashima, Mari Ishiguro-Watanabe. 2023. “KEGG for taxonomy-based analysis of pathways and genomes.” *Nucleic Acids Research* 51: 587-592. <https://doi.org/10.1093/nar/gkac963>

17. Galperin, Michael Y., Yuri I Wolf 1, Kira S. Makarova, Roberto Vera Alvarez, David Landsman, Eugene V. Koonin. 2021. “COG database update: focus on microbial diversity, model organisms, and widespread pathogens.” *Nucleic Acids Research* 49: 274-281. <https://doi.org/10.1093/nar/gkaa1018>

18. Zou, Yuanqiang, Wenbin Xue, Guangwen Luo, Ziqing Deng, Panpan Qin, Ruijin Guo, Haipeng Sun, et al. 2019. “1,520 reference genomes from cultivated human gut bacteria enable functional microbiome analyses.” *Nature Biotechnology* 37: 179-185. https://doi.org/10.1038/s41587-018-0008-8

19. Poyet, Mathilde, Mathieu Groussin, Sean M. Gibbons, Julian Avila-Pacheco, Xiaofang Jiang, Sean M. Kearney, Allison R. Perrotta, et al. 2019. “A library of human gut bacterial isolates paired with longitudinal multiomics data enables mechanistic microbiome research.” *Nature Medicine* 25: 1442-1452. https://doi.org/10.1038/s41591-019-0559-3

20. Forster, Samuel C., Nitin Kumar, Blessing O. Anonye, Alexandre Almeida, Elisa Viciani, Mark D. Stares, Matthew Dunn, et al. 2019. “A human gut bacterial genome and culture collection for improved metagenomic analyses.” *Nature Biotechnology* 37: 186-192. https://doi.org/10.1038/s41587-018-0009-7

21. Nishihara, Tamao, Morihiro Matsuda, Hiroshi Araki, Kazuya Oshima, Shinji Kihara, Tohru Funahashi, Iichiro Shimomura. 2006. “Effect of adiponectin on murine colitis induced by dextran sulfate sodium.” *Gastroenterology* 131: 853-861. <https://doi.org/10.1053/j.gastro.2006.06.015>

22. Mukaka Mavuto. 2012. Statistics corner: A guide to appropriate use of correlation coefficient in medical research. *Malawi Medical Journal* 24: 69-71. https://doi.org/10.4314/MMJ.V24I3

**Supplementary Taxon**

**Taxon 1: *Zhonglingia intestinalis* gen. nov. sp. nov**.

**Description of *Zhonglingia*** **gen. nov.** (Zhong.ling’i.a. N.L. fem. n. *Zhonglingia*, named in honor of the Chinese medical scientist Zhongling Cheng.)

The closest phylogenetic neighbor of Taxon 1 as represented by strain HA1519^T^ is *Dielma fastidiosa* JC13^T^ (Figure ST-1A). *D. fastidiosa* JC13^T^ and HA1519^T^ (16S rRNA sequence accession number in NMDC (National Microbiology Data Center) is NMDCN0001F43) has 16S rRNA gene identity of 93.23%, and their DNA G+C content is different by 0.8%. The genome of strain HA1519^T^ was sequenced and the NMDC accession number is NMDC60137304. Genome-based analysis on *D. fastidiosa* JC13^T^ and type strain HA1519^T^ revealed that the dDDH, ANI and POCP values between *D. fastidiosa* JC13^T^ (GCA_000313565) and strain HA1519^T^ are 21.90%, 67.86% and 41.70%, suggesting *D. fastidiosa* JC13^T^ and strain HA1519^T^ are from different genera. According to the phylogenomic tree (Figure ST-1C), the ANIs and dDDH values of genomes between strain HA1519^T^ and phylogenomically neighbored genomes were calculated, and the highest ANI and dDDH values are 67.86% and 21.90%, respectively, between the query genome of *Dielma fastidiosa* JC13^T^ (GCA_000313565); and difference in mol% G+C between genomes of strain HA1519^T^ and *D. fastidiosa* JC13^T^ is 0.8; OrthoANI heatmap (Figure ST-1D) shows the phylogenomic status of the corresponding neighbor species based on OrthoANI values. Cells of the strain HA1519^T^ are rod-shaped. The G+C content is around 38.7 mol%. The type species is *Zhonglingia intestinalis*.

**Description of *Zhonglingia intestinalis* sp. nov.** (in.tes.ti.na’lis. N.L. fem. adj. *intestinalis*, pertaining to the intestine, denoting the type strain was isolated from the intestine.)

Cells are rod-shaped with spiky ends (3 - 6 μm long × 0.5 μm wide, Figure ST-1B); cells appear singly without flagella and non-motile. Growth to stable phase occurs after 48 h incubation in mmGAM medium at 37°C, pH = 7.2. No significant fermentation products detected. The G+C content of the type strain HA1519^T^ is around 38.7 mol%. The type strain HA1519^T^ (= CGMCC 1.48484^T^ = KCTC 25719^T^) was isolated from the faeces of rheumatoid arthritis patients.


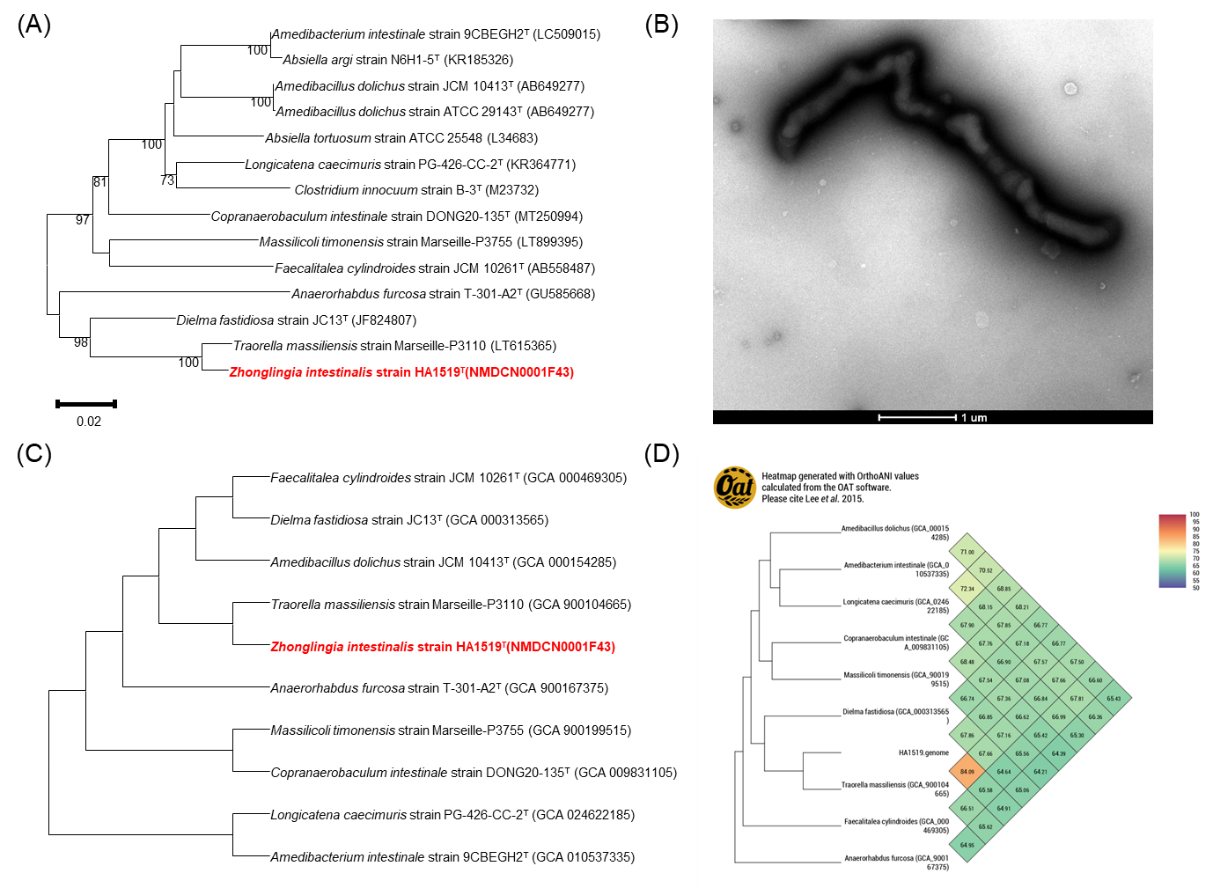


Figure ST-1. The Neighbor-joining phylogenetic tree based on 16S rRNA gene sequences (A) and the cell morphology (B) of strain HA1519^T^. GenBank accession numbers are given in parentheses. Percentages of bootstrap support are shown at branch nodes (values > 70 % are shown). Bootstrap value was 1000. Bar: 0.02 substitutions per nucleotide position. The phylogenomic tree based on genome-sequence-alignment (C) and OrthoANI heatmap (D) of strain HA1519^T^ and its neighbor species. Refseq ID of representative genomes are given in parentheses. **Taxon 2: *Paralentihominibacter* *butyricigenes* gen. nov. sp. nov.**

**Description of *Paralentihominibacter*** gen. nov. (Pa.ra.len.ti.ho.mi.ni.bac’ter. Gr. prep. para, next to; N.L. masc. n. *Lentihominibacter*, a bacterial genus; N.L. masc. n. *Paralentihominibacter*, a genus closely related to *Lentihominibacter*.)

The closest phylogenetic neighbor of Taxon 2 as represented by strain HA0442^T^ is *Lentihominibacter hominis* NSJ-24^T^ (Figure ST-2A). *L. hominis* NSJ-24^T^ and HA0442^T^ (16S rRNA sequence accession number in NMDC (National Microbiology Data Center) is NMDCN0001ERM) has 16S rRNA gene identity of 92.09%, and their DNA G+C content is different by 4.38%. The genome of strain HA0442^T^ was sequenced and the NMDC accession number is NMDC60137278. Genome-based analysis on *L. hominis* NSJ-24^T^ (GCA_014385065) and type strain HA0442^T^ revealed that the dDDH, ANI and POCP values between *L. hominis* NSJ-24^T^ and strain HA0442^T^ are 25.20%, 67.41% and 41.14%, suggesting *L. hominis* NSJ-24^T^ and strain HA0442^T^ are from different genera. According to the phylogenomic tree (Figure ST-2C), the ANIs and dDDH values of genomes between strain HA0442^T^ and phylogenomically neighbored genomes were calculated, and the highest ANI and dDDH values are 67.41% and 25.2%, respectively, between the query genome of *Lentihominibacter hominis* NSJ-24^T^ (GCA_014385065); and difference in mol% G+C between genomes of strain HA0442^T^ and *L. hominis* NSJ-24^T^ is 4.38; OrthoANI heatmap (Figure ST-2D) shows the phylogenomic status of the corresponding neighbor species based on OrthoANI values. Cells of the strain HA0442^T^ are rod-shaped with rounded ends. The G+C content is around 44.26 mol%. The type species is *Paralentihominibacter butyricigenes*.

**Description of *Paralentihominibacter* *butyricigenes* sp. nov. (**bu.ty.ri.ci’ge.nes. N.L. masc. n. *acidum butyricum*, butyric acid; Gr. suff. -genes, forming; N.L. part. adj. *butyricigenes*, butyric-acid producing, denoting the type strain produces butyric acid.**)**

Cells are rod-shaped with rounded ends (2.5 - 5.0 μm long × 0.4 μm wide, Figure ST-2B); cells appear singly without flagella and non-motile. Growth to stable phase occurs after 48 h incubation in mmGAM medium at 37°C, pH = 7.2. The main fermentation product is butyric acid. The G+C content of the type strain HA0442^T^ is around 44.26 mol%. The type strain HA0442^T^ (= CGMCC 1.48215^T^ = KCTC 25743^T^) was isolated from the faeces of rheumatoid arthritis patients.


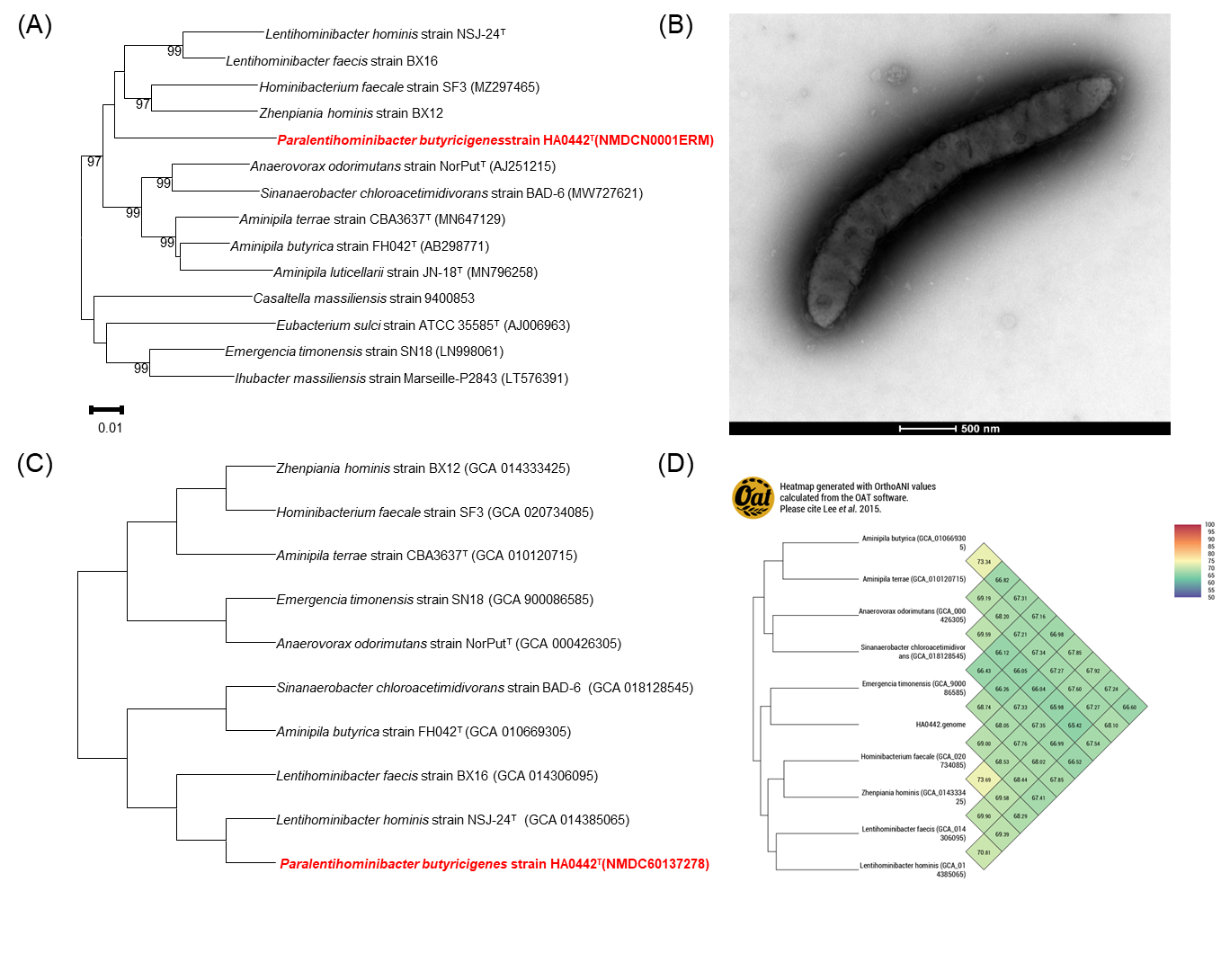


Figure ST-2. The Neighbor-joining phylogenetic tree based on 16S rRNA gene sequences (A) and the cell morphology (B) of strain HA0442^T^. GenBank accession numbers are given in parentheses. Percentages of bootstrap support are shown at branch nodes (values > 70 % are shown). Bootstrap value was 1000. Bar: 0.01 substitutions per nucleotide position. The phylogenomic tree based on genome-sequence-alignment (C) and OrthoANI heatmap (D) of strain HA0442^T^ and its neighbor species. Refseq ID of representative genomes are given in parentheses. **Taxon 3: *Zhongjingia baculiformis* gen. nov. sp. nov.**

**Description of *Zhongjingia* gen. nov.** **(**Zhong.jing’i.a. N.L. fem. n. *Zhongjingia*, named after Zhongjing Zhang, a medical scientist who has contributed to the treatment of rheumatic arthritis using traditional Chinese medicine.**)**

The closest phylogenetic neighbor of Taxon 3 as represented by strain HA0628^T^ is *Aminipila butyrica* FH042^T^ (Figure ST-3A). *A. butyrica* FH042^T^ and HA0628^T^ (16S rRNA sequence accession number in NMDC (National Microbiology Data Center) is NMDCN0001EV1) has 16S rRNA gene identity of 92.92%, and their DNA G+C content is different by 0.7%. The genome of strain HA0628^T^ was sequenced and the NMDC accession number is NMDC60137289. Genome-based analysis on *A. butyrica* FH042^T^ (GCA_010669305) and type strain HA0628^T^ revealed that the dDDH, ANI and POCP values between *A. butyrica* FH042^T^ and strain HA0628^T^ are 22.90%, 66.56% and 37.84%, suggesting *A. butyrica* FH042^T^ and strain HA0628^T^ are from different genera. According to the phylogenomic tree (Figure ST-3C), the ANIs and dDDH values of genomes between strain HA0628^T^ and phylogenomically neighbored genomes were calculated, and the highest ANI, dDDH values are 68.52% and 31.7%, respectively, between the query genome of *Lentihominibacter hominis* NSJ-24^T^ (GCA_014385065); and difference in mol% G+C between genomes of strain HA0628^T^ and *L. hominis* NSJ-24^T^ is 5.71; OrthoANI heatmap (Figure ST-3D) shows the phylogenomic status of the corresponding neighbor species based on OrthoANI values. Cells of the strain HA0628^T^ are rod-shaped. The G+C content is around 45.59 mol%. The type species is *Zhongjingia baculiformis*.

**Description of *Zhongjingia baculiformis* sp. nov. (**ba.cu.li.for’mis. L. fem. n. *baculus*, rod; L. fem. adj. suff. *-formis*, of the shape of; N.L. fem. adj. *baculiformis*, rod-shaped, denoting the shape of the type strain of the species.**)**

Cells are rod-shaped with oval ends (1.6 - 2.5 μm long × 0.5 μm wide, Figure ST-3B); cells appear singly without flagella and non-motile. Growth to stable phase occurs after 72 h incubation in mmGAM medium at 37°C, pH = 7.2. The main fermentation product is small amount of butyric acid. The G+C content of the type strain HA0628^T^ is around 45.59 mol%. The type strain HA0628^T^ (= CGMCC 1.48322^T^) was isolated from the faeces of rheumatoid arthritis patients.


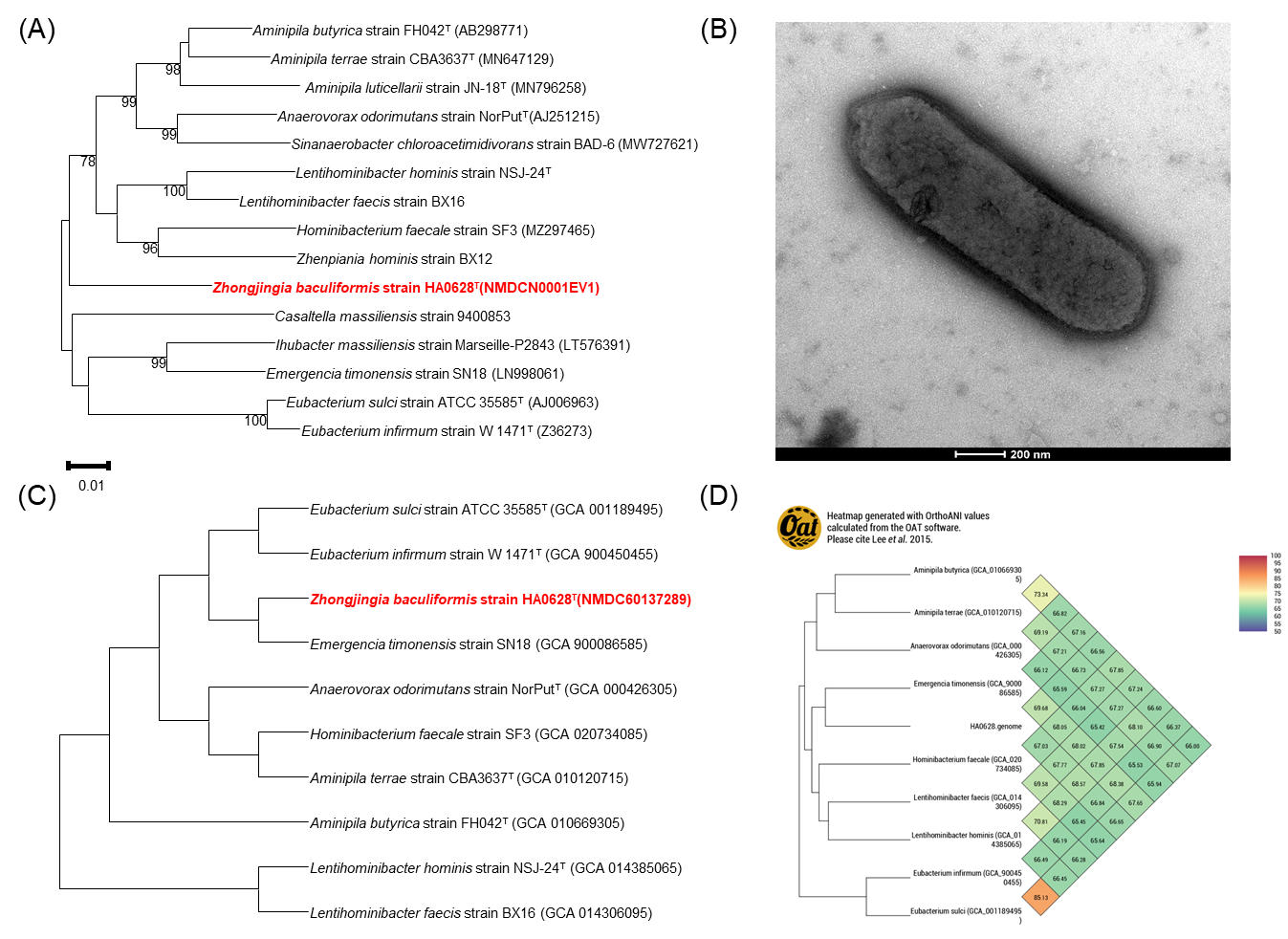


Figure ST-3. The Neighbor-joining phylogenetic tree based on 16S rRNA gene sequences (A) and the cell morphology (B) of strain HA0628^T^. GenBank accession numbers are given in parentheses. Percentages of bootstrap support are shown at branch nodes (values > 70 % are shown). Bootstrap value was 1000. Bar: 0.01 substitutions per nucleotide position. The phylogenomic tree based on genome-sequence-alignment (C) and OrthoANI heatmap (D) of strain HA0628^T^ and its neighbor species. Refseq ID of representative genomes are given in parentheses.**Taxon 4: *Naizhengia* *acetigignens* gen. nov. sp. nov.**

**Description of *Naizhengia* gen. nov.** (Nai.zheng’i.a. N.L. fem. n. *Naizhengia*, named in honor of the Chinese medical scientist Naizheng Zhang.)

The closest phylogenetic neighbor of Taxon 4 as represented by strain HA0073^T^ is *Faecalicatena* *fissicatena* DSM 3598^T^ (Figure ST-4A). *F. fissicatena* DSM 3598^T^ and HA0073^T^ (16S rRNA sequence accession number in NMDC (National Microbiology Data Center) is NMDCN0001EN0) has 16S rRNA gene identity of 92.90%, and their DNA G+C content is different by 8.3%. The genome of strain HA0073^T^ was sequenced and the NMDC accession number is NMDC60137272. Genome-based analysis on *F. fissicatena* DSM 3598^T^ and type strain HA0073^T^ revealed that the dDDH, ANI and POCP values between *F. fissicatena* DSM 3598^T^ (GCA_016900595) and strain HA0073^T^ are 28.20%, 68.43% and 40.37%, suggesting *F. fissicatena* DSM 3598^T^ and strain HA0073^T^ are from different genera. According to the phylogenomic tree (Figure ST-4C), the ANIs and dDDH values of genomes between strain HA0073^T^ and phylogenomically neighbored genomes were calculated, and the highest ANI and dDDH values are 72.05% and 32.9%, respectively, between the query genome of *Coprococcus comes* ATCC 27758^T^ (GCA_025149785); and difference in mol% G+C between genomes of strain HA0073^T^ and *C. comes* ATCC 27758^T^ is 2.59; OrthoANI heatmap (Figure ST-4D) shows the phylogenomic status of the corresponding neighbor species based on OrthoANI values. Cells of the strain HA0073^T^ are long rod-shaped. The G+C content is around 45.11 mol%. The type species is *Naizhengia* *acetigignens*.

**Description of *Naizhengia* *acetigignens* sp. nov.** (a.ce.ti.gi.gnens. L. fem. n. *acetum*, vinegar, refer to acetic acid; L. inf. v. *gignere*, to produce; N.L. part. adj. *acetigignens*, acetic acid-producing, denoting the type strain produces acetic acid.)

Cells are long rod-shaped with square ends (3.5 - 8.5 μm long × 0.5 μm wide, Figure ST-4B); cells appear singly without flagella and non-motile. Growth to stable phase occurs after 48 h incubation in mmGAM medium at 37°C, pH = 7.2. The main fermentation product is acetic acid, and small amounts of butyric acid can also be produced. The G+C content of the type strain HA0073^T^ is around 45.11 mol%. The type strain HA0073^T^ (= CGMCC 1.48065^T^ = KCTC 25694^T^) was isolated from the faeces of rheumatoid arthritis patients.


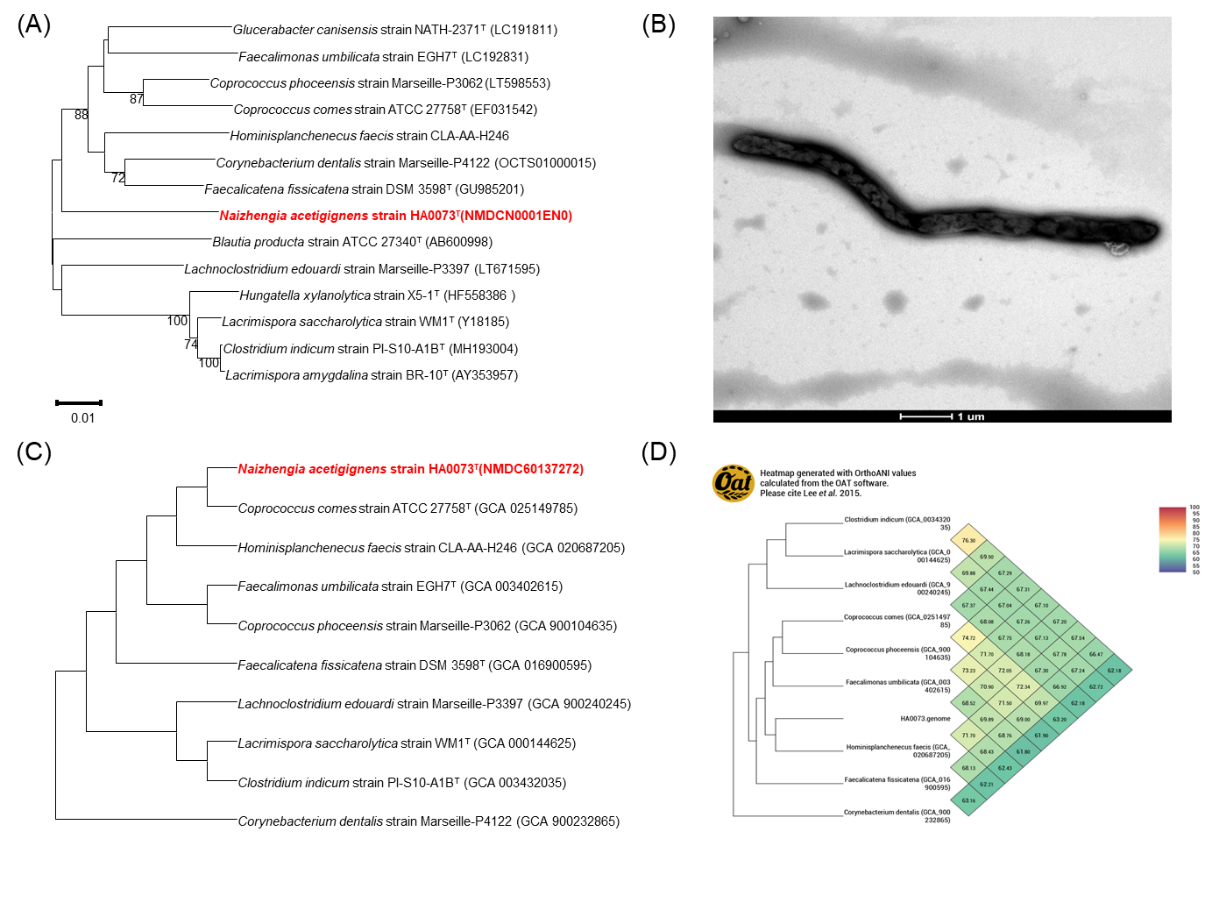


Figure ST-4. The Neighbor-joining phylogenetic tree based on 16S rRNA gene sequences (A) and the cell morphology (B) of strain HA0073^T^. GenBank accession numbers are given in parentheses. Percentages of bootstrap support are shown at branch nodes (values > 70 % are shown). Bootstrap value was 1000. Bar: 0.01 substitutions per nucleotide position. The phylogenomic tree based on genome-sequence-alignment (C) and OrthoANI heatmap (D) of strain HA0073^T^ and its neighbor species. Refseq ID of representative genomes are given in parentheses.**Taxon 5: *Sanxizhangella immobilis* gen. nov. sp. nov.**

**Description of *Sanxizhangella* gen. nov. (**San.xi.zhang.el’la. N.L. fem. dim. n. *Sanxizhangella*, named after San-Xi Zhang, a Chinese medical scientist, in honor of Zhang's contributions regarding the studies and treatment of rheumatoid arthritis.**)**

The closest phylogenetic neighbor of Taxon 5 as represented by strain HA0013^T^ is *Clostridium fessum* SNUG30386^T^ (Figure ST-5A). *C. fessum* SNUG30386^T^ and HA0013^T^ (16S rRNA sequence accession number in NMDC (National Microbiology Data Center) is NMDCN0001ELA) has 16S rRNA gene identity of 93.95%, and their DNA G+C content is different by 2.1%. The genome of strain HA0013^T^ was sequenced and the NMDC accession number is NMDC60137267. Genome-based analysis on *C. fessum* SNUG30386^T^ and type strain HA0013^T^ revealed that the dDDH, ANI and POCP values between *C. fessum* SNUG30386^T^ (GCA_003024715) and strain HA0013^T^ are 36.2%, 74.31% and 51.59%. According to phylogenetic tree (Figure ST-5A), strain HA0013^T^ and *C. fessum* SNUG30386^T^ were not clustered in a clade, suggesting they were from different genera. According to the phylogenomic tree (Figure ST-5C), the ANIs and dDDH values of genomes between strain HA0013^T^ and phylogenomically neighbored genomes were calculated, and the highest ANI and dDDH values are 74.31% and 36.2%, respectively, between the query genome of *C. fessum* SNUG30386^T^; and difference in mol% G+C between genomes of strain HA0013^T^ and *C. fessum* SNUG30386^T^ is 2.05; OrthoANI heatmap (Figure ST-5D) shows the phylogenomic status of the corresponding neighbor species based on OrthoANI values. Cells of the strain HA0013^T^ are fusiform with rounded ends. The G+C content is around 46.24 mol%. The type species is *Sanxizhangella immobilis.*

**Description of *Sanxizhangella immobilis*** **sp. nov.** (im.mo’bi.lis. L. fem. adj. *immobilis*, non-motile, nonmotile, indicating the type strain is non-motile.)

Cells are fusiform with rounded ends (2.5 - 4.6 μm long × 1.5 μm wide, Figure ST-5B); cells appear singly without flagella and non-motile. Growth to stable phase occurs after 48 h incubation in mmGAM medium at 37°C, pH = 7.2. The main fermentation product is acetic, and small amounts of propionic, isobutyric and butyric acid can also be produced. The G+C content of the type strain HA0013^T^ is around 46.24 mol%. The type strain HA0013^T^ (= CGMCC 1.48011^T^ =KCTC 25673^T^) was isolated from the faeces of rheumatoid arthritis patients.


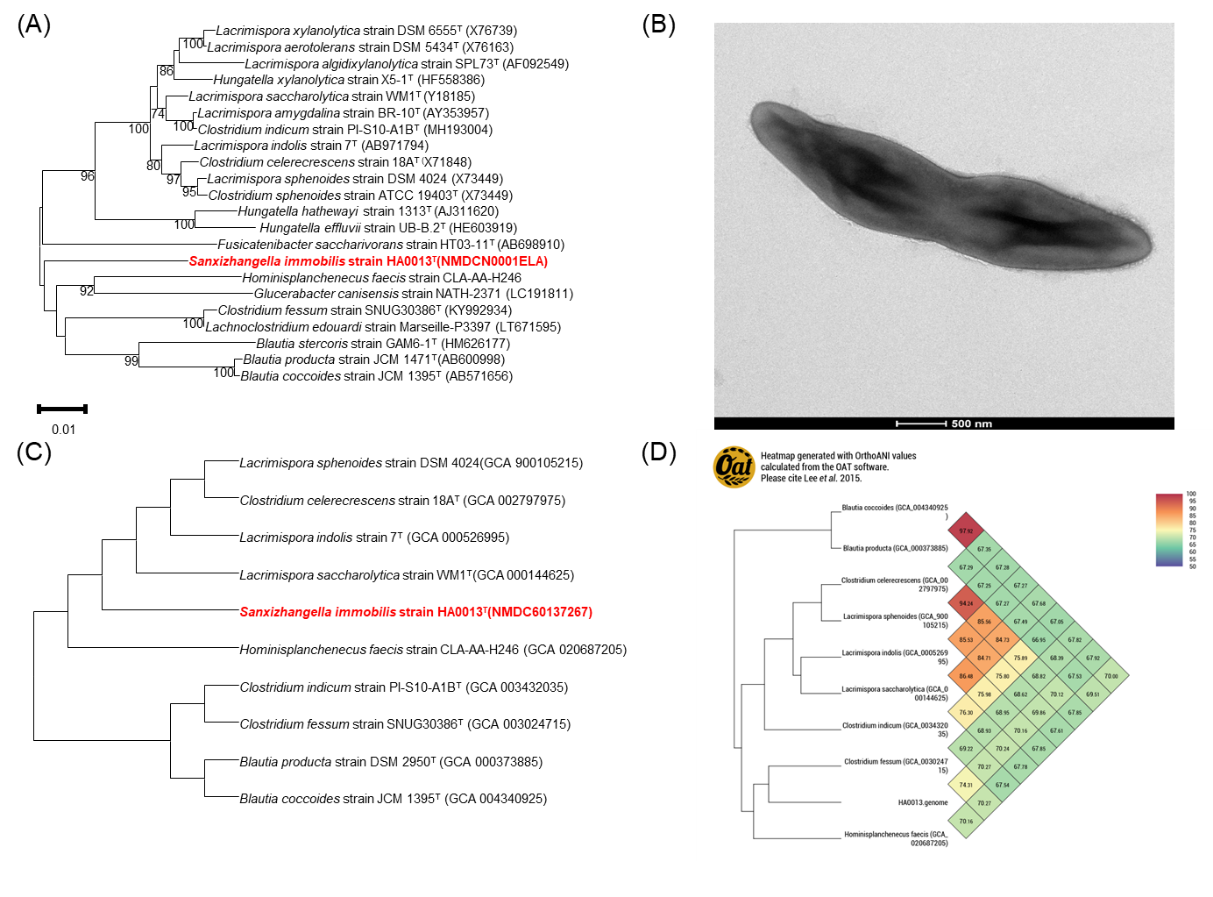


Figure ST-5. The Neighbor-joining phylogenetic tree based on 16S rRNA gene sequences (A) and the cell morphology (B) of strain HA0013^T^. GenBank accession numbers are given in parentheses. Percentages of bootstrap support are shown at branch nodes (values > 70 % are shown). Bootstrap value was 1000. Bar: 0.01 substitutions per nucleotide position. The phylogenomic tree based on genome-sequence-alignment (C) and OrthoANI heatmap (D) of strain HA0013^T^ and its neighbor species. Refseq ID of representative genomes are given in parentheses. **Taxon 6: *Yonghella fusiformis* gen. nov. sp. nov**.

**Description of *Yonghella* gen. nov.** (Yong.hel’la. N.L. fem. dim. n. *Yonghella*, named after Yonghe Yan, a medical scientist who has contributed to the treatment of rheumatic arthritis using traditional Chinese medicine and the author of the medical script “Ji Sheng Fang”.)

The closest phylogenetic neighbor of Taxon 6 as represented by strain HA1168^T^ is *Lacrimispora saccharolytica* WM1^T^ (Figure ST-6A). *L. saccharolytica* WM1^T^ and HA1168^T^ (16S rRNA sequence accession number in NMDC is NMDCN0001EVT) has 16S rRNA gene identity of 94.73%, and their DNA G+C content is different by 3.2%. The genome of strain HA1168^T^ was sequenced and the NMDC accession number is NMDC60137292. Genome-based analysis on *L. saccharolytica* WM1^T^ and type strain HA1168^T^ revealed that the dDDH, ANI and POCP values between *L. saccharolytica* WM1^T^ (GCA_000144625) and strain HA1168^T^ are 25.00%, 70.08% and 44.61%, suggesting *L. saccharolytica* WM1^T^ and strain HA1168^T^ are from different genera. According to the phylogenomic tree (Figure ST-6C), the ANIs and dDDH values of genomes between strain HA1168^T^ and phylogenomically neighbored genomes were calculated, and the highest ANI and dDDH values are 70.08% and 25.00%, respectively, between the query genome of *Lacrimispora saccharolytica* WM1^T^; and difference in mol% G+C between genomes of strain HA1168^T^ and *L. saccharolytica* WM1^T^ is 3.15; OrthoANI heatmap (Figure ST-6D) shows the phylogenomic status of the corresponding neighbor species based on OrthoANI values. Cells of the strain HA1168^T^ are spindle -shaped. The G+C content is around 42.91 mol%. The type species is *Yonghella fusiformis*.

***Yonghella fusiformis* sp. nov. (**fu.si.for’mis. L. fem. n. *fusus*, spindle; L. fem. n. *forma*, form, shape; N.L. fem. adj. *fusiformis*, spindle-shaped, denoting the shape of the type strain of the species）

Cells are fusiform with spiky ends (3.0 - 5.0 μm long × 0.8 - 1.2 μm wide, Figure ST-6B); cells appear in dividing pairs without flagella and non-motile. Growth to stable phase occurs after 48 h incubation in mmGAM medium at 37°C, pH = 7.2. The main fermentation product is small amount of acetic acid. The G+C content of the type strain HA1168^T^ is around 42.91 mol%. The type strain HA1168^T^ (= CGMCC 1.48350^T^= KCTC 25681^T^) was isolated from the faeces of rheumatoid arthritis patients.


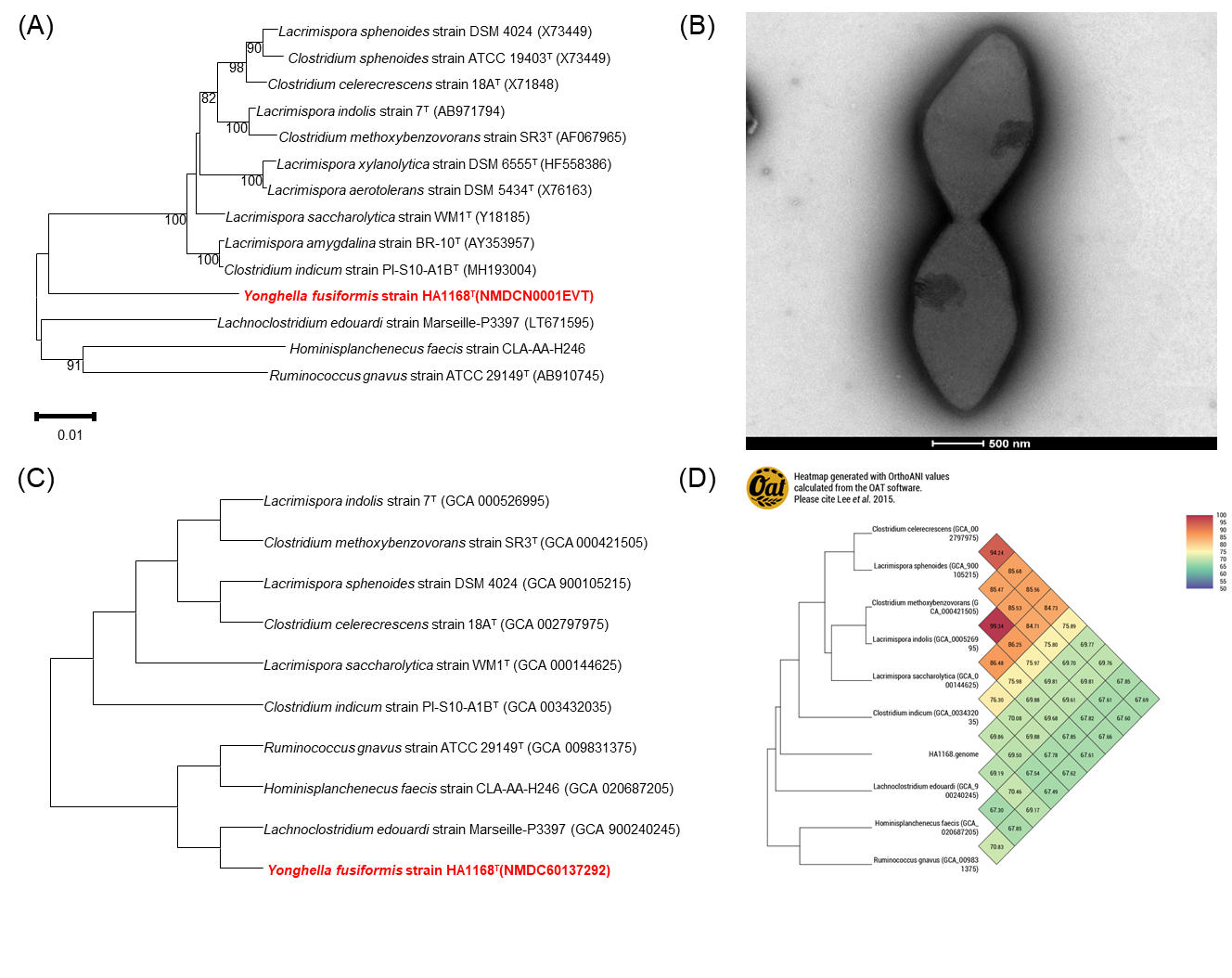


Figure ST-6. The Neighbor-joining phylogenetic tree based on 16S rRNA gene sequences (A) and the cell morphology (B) of strain HA1168^T^. GenBank accession numbers are given in parentheses. Percentages of bootstrap support are shown at branch nodes (values > 70 % are shown). Bootstrap value was 1000. Bar: 0.01 substitutions per nucleotide position. The phylogenomic tree based on genome-sequence-alignment (C) and OrthoANI heatmap (D) of strain HA1168^T^ and its neighbor species. Refseq ID of representative genomes are given in parentheses.

**Taxon 7: *Guangjia hominis* gen. nov. sp. nov.**

**Description of *Guangjia* gen. nov. (**Guang.ji.a. N.L. fem. n. *Guangjia*, in honor of Guangji Shi, in recognition of his contributions to the treatment of rheumatic arthritis using traditional Chinese medicine.**)**

The closest phylogenetic neighbor of Taxon 7 as represented by strain HA1523^T^ is *Clostridium viride* T2-7^T^ (Figure ST-7A). *C. viride* T2-7^T^ and strain HA1523^T^ (16S rRNA sequence accession number in NMDC (National Microbiology Data Center) is NMDCN0001F47) has 16S rRNA gene identity of 93.5%, and their DNA G+C content is different by 12.5%. The genome of strain HA1523^T^ was sequenced and the NMDC accession number is NMDC60137305. Genome-based analysis on *C. viride* T2-7^T^ and type strain HA1523^T^ revealed that the dDDH, ANI and POCP values between *C. viride* T2-7^T^ (GCA_000620945) and strain HA1523^T^ are 21.00%, 67.36% and 40.68%, suggesting *C. viride* T2-7^T^ and strain HA1523^T^ are from different genera. According to the phylogenominc tree (Figure ST-7C), the ANIs and dDDH values of genomes between strain HA1523^T^ and phylogenomically neighbored genomes were calculated, and the highest ANI and dDDH values are 79.82% and 23.5%, respectively, between the query genome of *Pseudoflavonifractor gallinarum* Cla-CZ-98^T^ (GCA_014982855); and difference in mol% G+C between genomes of strain HA1523^T^ and *P. gallinarum* Cla-CZ-98^T^ is 1.87; OrthoANI heatmap (Figure ST-7D) shows the phylogenomic status of the corresponding neighbor species based on OrthoANI values. Cells of the strain HA1523^T^ are rod shaped. The G+C content is around 61.81 mol%. The type species is *Guangjia hominis*.

**Description of** ***Guangjia hominis* sp. nov. (**ho’mi.nis. L. gen. fem. n. *hominis*, of a human being, referring to the human gut habitat of the type strain.**)**

Cells are rod-shaped with spiky ends (2.4 - 8.0 μm long × 0.5 μm wide, Figure ST-7B); cells appear singly with no flagella and non-motile. Growth to stable phase occurs after 48 h incubation in mmGAM medium at 37°C, pH = 7.2. The main fermentation product is butyric acid. The HA1523^T^ G+C content of the type strain is around 61.81 mol%. The type strain HA1523^T^ (= CGMCC 1.17999^T^ = KCTC 25720^T^) was isolated from the faeces of rheumatoid arthritis patients.


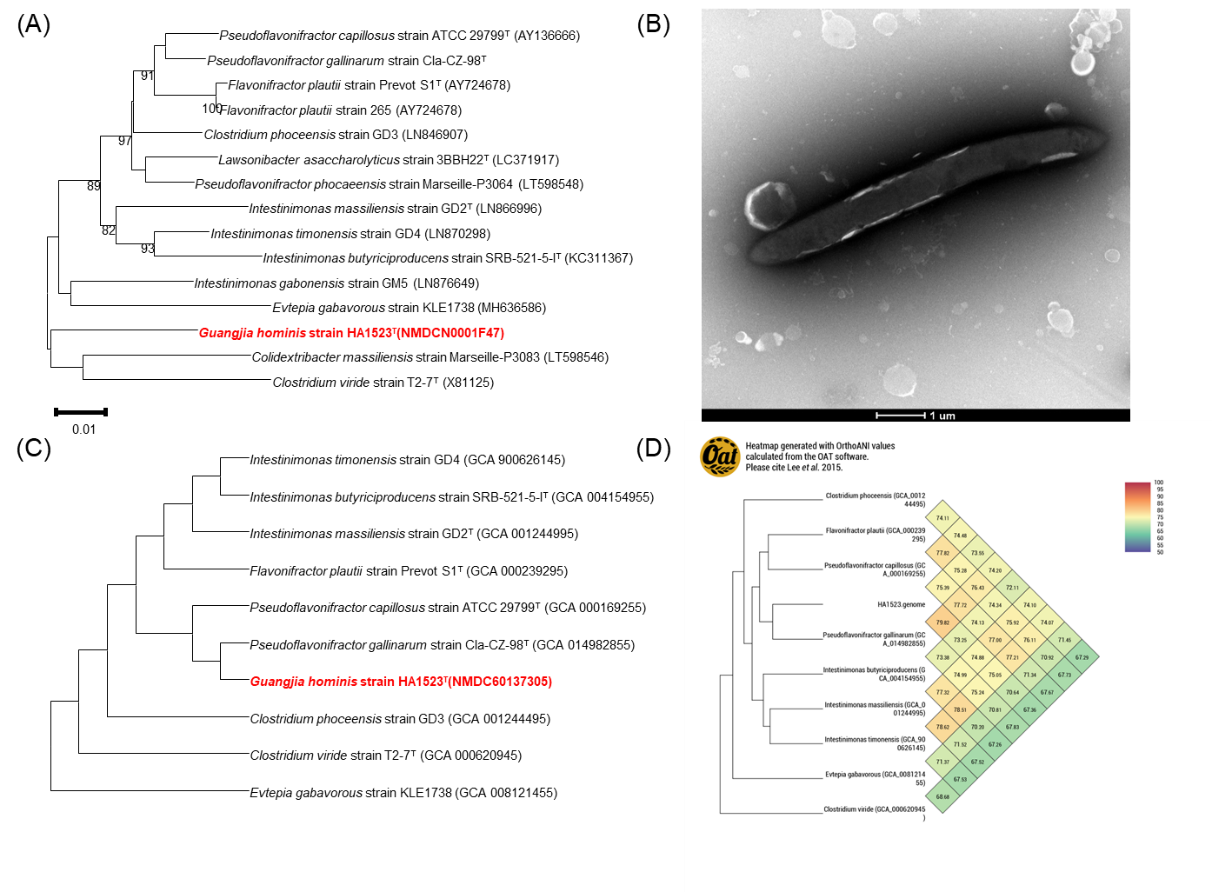


Figure ST-7. The Neighbor-joining phylogenetic tree based on 16S rRNA gene sequences (A) and the cell morphology (B) of strain HA1523^T^. GenBank accession numbers are given in parentheses. Percentages of bootstrap support are shown at branch nodes (values > 70 % are shown). Bootstrap value was 1000. Bar: 0.01 substitutions per nucleotide position. The phylogenomic tree based on genome-sequence-alignment (C) and OrthoANI heatmap (D) of strain HA1523^T^ and its neighbor species. Refseq ID of representative genomes are given in parentheses. **Taxon 8: *Jirenia arthritidis* gen. nov. sp. nov.**

**Description of *Jirenia* gen. nov.** (Ji.ren’i.a. N.L. fem. n. *Jirenia*, named in honor of the Chinese medical scientist Jiren Li, who did great contribution in the treatment of rheumatoid arthritis.)

The closest phylogenetic neighbor of Taxon 8 as represented by strain HA0569^T^ is *Hydrogenoanaerobacterium saccharovorans* SW512^T^ (Figure ST-8A). *H. saccharovorans* SW512^T^ and HA0569^T^ (16S rRNA sequence accession number in NMDC is NMDCN0001EU1) have 16S rRNA gene identity of 91.28%, and their DNA G+C content is different by 10.6%. The genome of strain of strain HA0569^T^ was sequenced and the NMDC accession number is NMDC60137288. Genomic analysis of *H. saccharovorans* SW512^T^ (GCA_003814745) and type strain HA0569^T^ revealed that the dDDH, ANI and POCP values between *H. saccharovorans* SW512^T^ and strain HA0569^T^ are 19.50%, 65.0% and 34.80%, suggesting *H. saccharovorans* SW512^T^ and strain HA0569^T^ are from different genera. According to the phylogenomic tree (Figure ST-8C), the ANIs and dDDH values of genomes between strain HA0569^T^ and phylogenomically neighbored genomes were calculated, and the highest ANI and dDDH values are 68.81% and 22.8%, respectively, between the query genome of *Ligaoa zhengdingensis* NSJ-31^T^ (GCA_014384885); and difference in mol% G+C between genomes of strain HA0569^T^ and *L. zhengdingensis* NSJ-31^T^ is 3.7; OrthoANI heatmap (Figure ST-8D) shows the phylogenomic status of the corresponding neighbor species based on OrthoANI values. Cells of the strain HA0569^T^ are rod-shaped with pointed ends. The G+C content is around 53.32 mol%. The type species is *Jirenia arthritidis*.

**Description of *Jirenia arthritidis* sp. nov.** (ar.thri’ti.dis. Gr. fem. n. *arthron*, joint; N.L. fem. n. suff. -*itis*, inflammation; N.L. gen. fem. n. *arthritidis*, of arthritis, denoting the type strain was isolated from the faeces of a rheumatoid arthritis patient.)

Cells are rod-shaped with pointed ends (5.0 - 9.0 μm long × 0.6 μm wide, Figure ST-8B); cells appear singly without flagella and non-motile. Growth to stable phase occurs after 48h incubation in mmGAM medium at 37°C, pH = 7.2. No significant fermentation products detected. The G+C content of the type strain HA0569^T^ is around 53.32 mol%. The type strain HA0569^T^ (= CGMCC 1.48290^T^ = KCTC 25701^T^) was isolated from the faeces of rheumatoid arthritis patients.


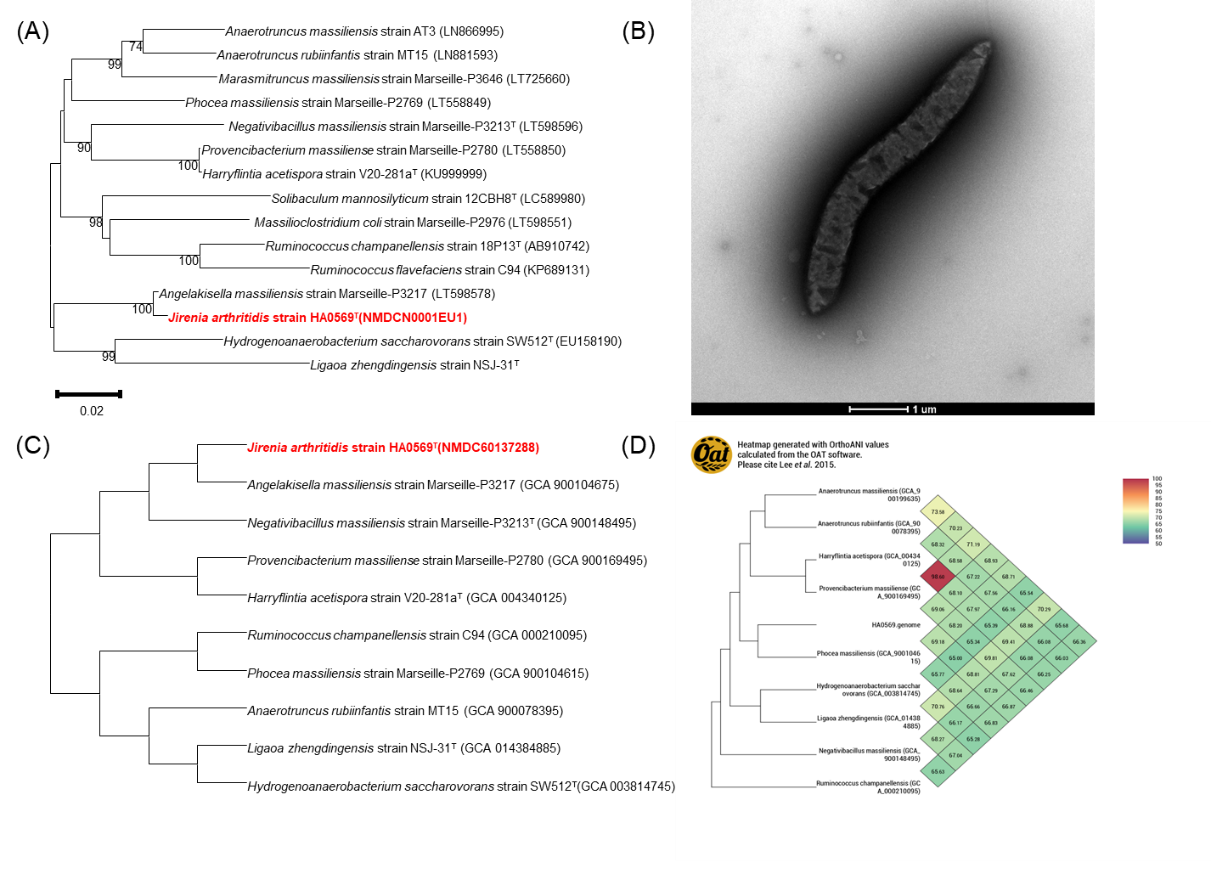


Figure ST-8. The Neighbor-joining phylogenetic tree based on 16S rRNA gene sequences (A) and the cell morphology (B) of strain HA0569^T^. GenBank accession numbers are given in parentheses. Percentages of bootstrap support are shown at branch nodes (values > 70 % are shown). Bootstrap value was 1000. Bar: 0.02 substitutions per nucleotide position. The phylogenomic tree based on genome-sequence-alignment (C) and OrthoANI heatmap (D) of strain HA0569^T^ and its neighbor species. Refseq ID of representative genomes are given in parentheses.**Taxon 9: *Qingyuzengella butyriciproducens* gen. nov. sp. nov.**

**Description of *Qingyuzengella* gen. nov. (**Qing.yu.zeng.el’la. N.L. fem. dim. n. *Qingyuzengella*, named in honor of the Chinese scientist Qingyu Zeng)

The closest phylogenetic neighbor of Taxon 9 as represented by strain HA0434^T^ is *Subdoligranulum variabile* Bl 114^T^ (Figure ST-9A). *S. variabile* Bl 114^T^ and strain HA0434^T^ (16S rRNA sequence accession number in NMDC is NMDCN0001ERE) has 16S rRNA gene identity of 93.71%, and their DNA G+C content is different by 1.2%. The genome of strain HA0434^T^ was sequenced and the NMDC accession number is NMDC60137274. Genome-based analysis on *S. variabile* Bl 114^T^ and type strain HA0434^T^ revealed that the dDDH, ANI and POCP values between *S. variabile* Bl 114^T^ (GCA_025152575) and strain HA0434^T^ are 20.20%, 70.99% and 46.58%, suggesting *S. variabile* Bl 114^T^ and strain HA0434^T^ are from different genera. According to the phylogenomic tree (Figure ST-9C), the ANIs and dDDH values of genomes between strain HA0434^T^ and phylogenomically neighbored genomes were calculated, and the highest ANI and dDDH values are 72.63% and 20.3%, respectively, between the query genome of *Faecalibacterium gallinarum* ic1379^T^ (GCA_022180365); and difference in mol% G+C between genomes of strain HA0434^T^ and *F. gallinarum* ic1379^T^ is 0.13; OrthoANI heatmap (Figure ST-9D) shows the phylogenomic status of the corresponding neighbor species based on OrthoANI values. Cells of the strain HA0434^T^ are rod-shaped. The G+C content is around 59.15 mol%. The type species is *Qingyuzengella butyriciproducens*.

**Description of *Qingyuzengella butyriciproducens* sp. nov. (**bu.ty.ri.ci.pro.du’cens. N.L. fem. n. *acidum butyricum*, butyric acid; L. pres. part. *producens*, producing; N.L. part. adj. *butyriciproducens*, producing butyric acid, denoting the type strain is a butyrate-producing bacterium.**)**

Cells are rod-shaped with square ends (2.2 - 10.0 μm long × 0.7 μm wide, Figure ST-9B); cells appear in dividing pairs without flagella and non-motile. Growth to stable phase occurs after 48 h incubation in mmGAM medium at 37°C, pH = 7.2. The main fermentation product is butyric acid. The G+C content of the type strain HA0434^T^ is around 59.15 mol%. The type strain HA0434^T^ (= CGMCC 1.48207^T^ = KCTC 25712^T^) was isolated from the faeces of rheumatoid arthritis patients.


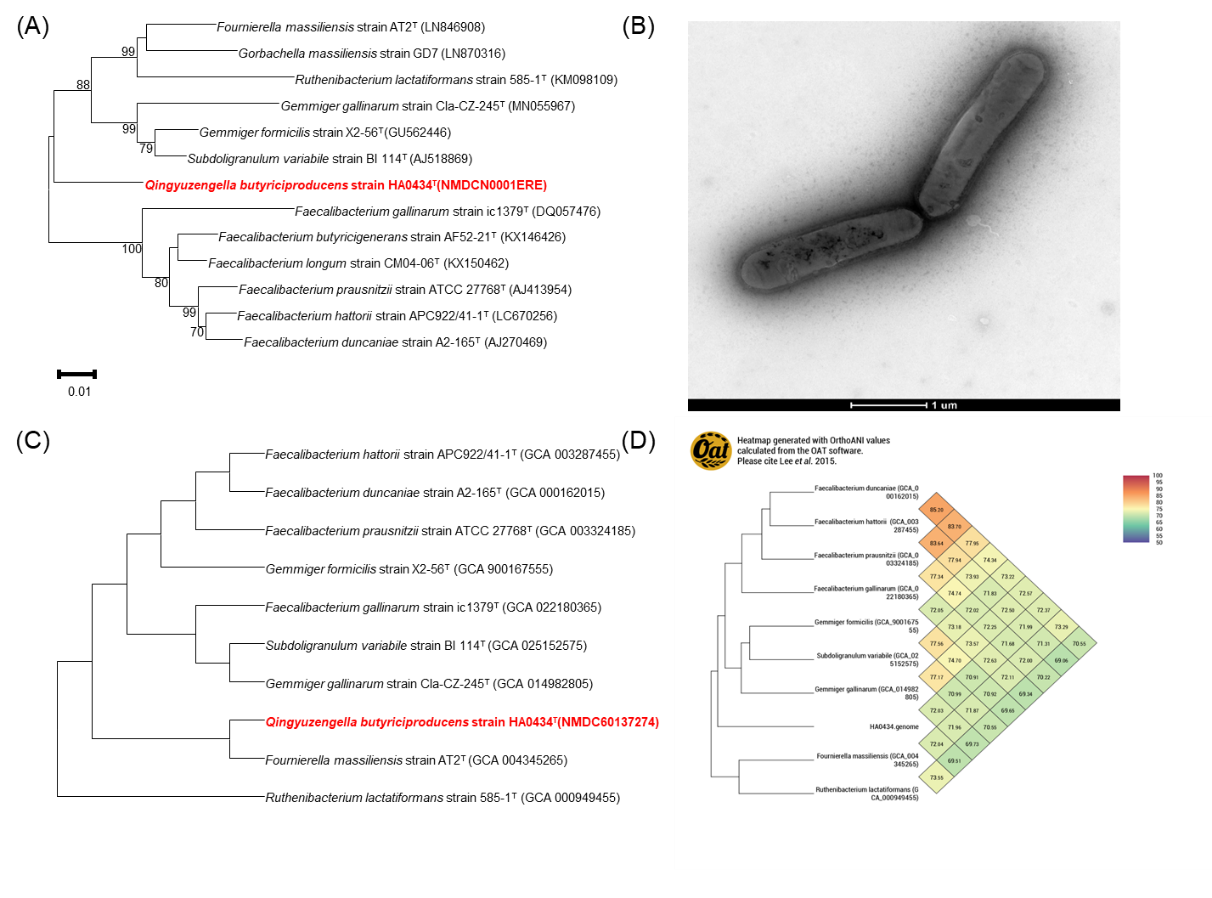


Figure ST-9. The Neighbor-joining phylogenetic tree based on 16S rRNA gene sequences (A) and the cell morphology (B) of strain HA0434^T^. GenBank accession numbers are given in parentheses. Percentages of bootstrap support are shown at branch nodes (values > 70 % are shown). Bootstrap value was 1000. Bar: 0.01 substitutions per nucleotide position. The phylogenomic tree based on genome-sequence-alignment (C) and OrthoANI heatmap (D) of strain HA0434^T^ and its neighbor species. Refseq ID of representative genomes are given in parentheses. **Taxon 10: *Mengyingia acetigenes* gen. nov. sp. nov.**

**Description of *Mengyingia* gen. nov.** (Meng.ying’i.a. N.L. fem. n. *Mengyingia*, named in honor of the Chinese medical scientist Mengying Wang.)

The closest phylogenetic neighbor of Taxon 10 as represented by strain HA1243^T^ is *Acutalibacter muris* KB18^T^ (Figure ST-10A). *A. muris* KB18^T^ and HA1243^T^ (16S rRNA sequence accession number in NMDC (National Microbiology Data Center) is NMDCN0001F28) has 16S rRNA gene identity of 93.56%, and their DNA G+C content is different by 2.4%. The genome of strain HA1243^T^ was sequenced and the NMDC accession number is NMDC60137296. Genome-based analysis on *A. muris* KB18^T^ and type strain HA1243^T^ revealed that the dDDH, ANI and POCP values between *A. muris* KB18^T^ (GCA_002201475) and strain HA1243^T^ are 26.70%, 68.25% and 44.06%, suggesting *A. muris* KB18^T^ and strain HA1243^T^are from different genera. According to the phylogenomic tree (Figure ST-10C), the ANIs and dDDH values of genomes between strain HA1243^T^ and phylogenomically neighbored genomes were calculated, and the highest ANI and dDDH values are 69.33% and 24.10%, respectively, between the query genome of *Caproiciproducens galactitolivorans* BS-1^T^ (GCA_004768785); and difference in mol% G+C between genomes of strain HA1243^T^ and *C. galactitolivorans* BS-1^T^ is 4.0; OrthoANI heatmap (Figure ST-10D) shows the phylogenomic status of the corresponding neighbor species based on OrthoANI values. Cells of the strain HA1243^T^ are chained rods with tapered ends. The G+C content is around 52.16 mol%. The type species is *Mengyingia* *acetigenes*.

***Mengyingia acetigenes* sp. nov.** (a.ce.ti’ge.nes. L. fem. n. *acetum*, vinegar; Gr. ind. v. *gennaô*, to produce; N.L. gen. fem. adj. *acetigenes*, acetate producing, indicating the type strain produces acetate.)

Cells are chained rods with tapered ends (2.2 - 8.0 μm long × 1.2 μm wide, Figure ST-10B); cells appear singly without flagella and non-motile. Growth to stable phase occurs after 48 h incubation in mmGAM medium at 37°C, pH = 7.2. The main fermentation product is acetic acid and small amounts of isobutyric and butyric acid can also be produced. The G+C content of the type strain HA1243^T^ is around 52.16 mol%. The type strain HA1243^T^ (= CGMCC 1.48425^T^ = KCTC 25683^T^) was isolated from the faeces of rheumatoid arthritis patients.


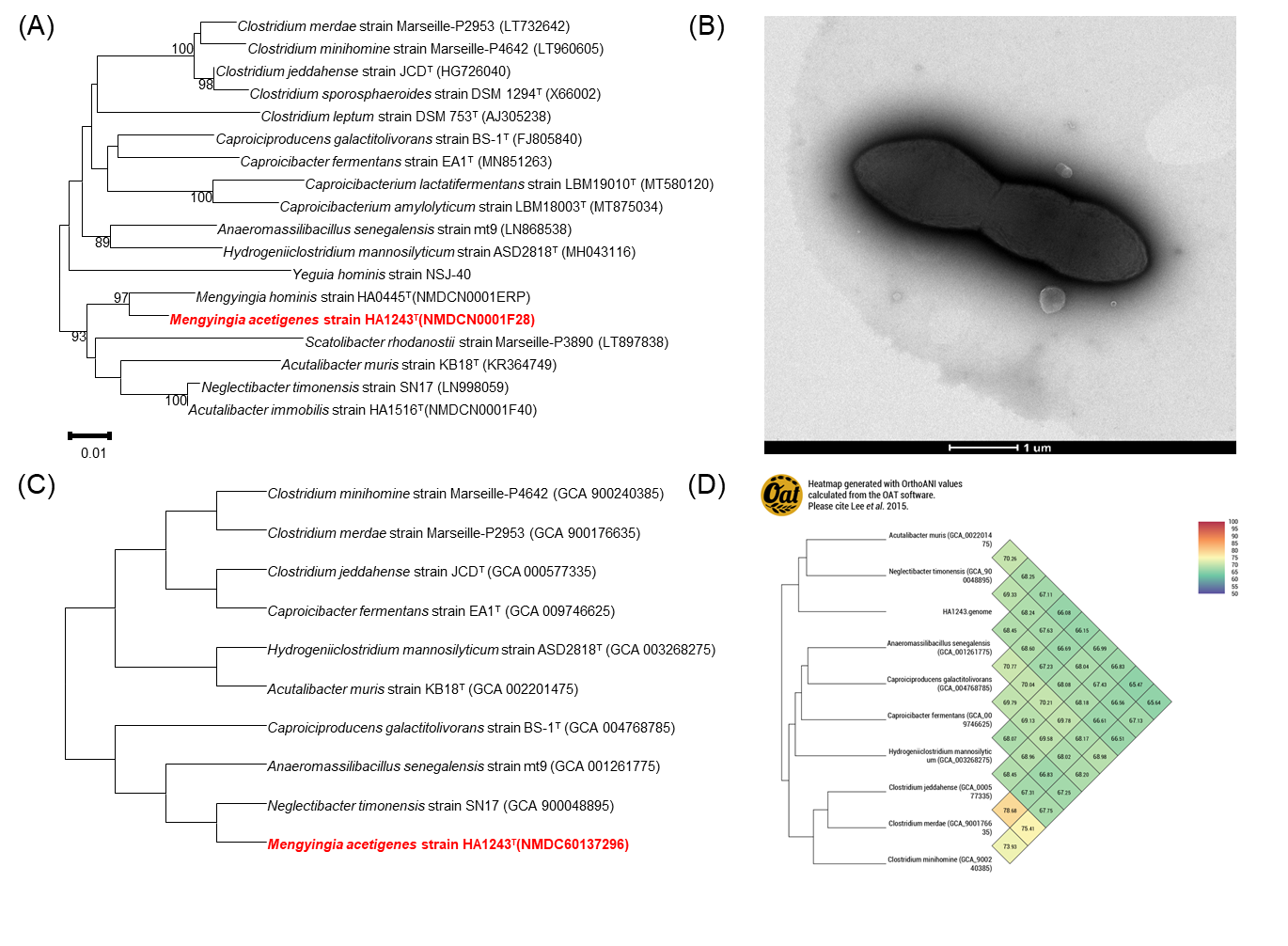


Figure ST-10. The Neighbor-joining phylogenetic tree based on 16S rRNA gene sequences (A) and the cell morphology (B) of strain HA1243^T^. GenBank accession numbers are given in parentheses. Percentages of bootstrap support are shown at branch nodes (values > 70 % are shown). Bootstrap value was 1000. Bar: 0.01 substitutions per nucleotide position. The phylogenomic tree based on genome-sequence-alignment (C) and OrthoANI heatmap (D) of strain HA1243^T^ and its neighbor species. Refseq ID of representative genomes are given in parentheses.

**Taxon 11: *Mengyingia hominis* sp. nov.**

**Description of *Mengyingia hominis* sp. nov.** (ho’mi.nis. L. gen. masc. n. *hominis*, of a human being, indicating that the type strain was isolated from a human.)

The taxon 11, represented by strain HA0445^T^ (16S rRNA sequence accession number in NMDC is NMDCN0001ERP), is phylogenetically closest to the validly published strain *Acutalibacter muris* KB18^T^, with 16S rRNA gene identity of 93.80%. Phylogenetic tree shows that strain HA0445^T^ clusters with *Mengyingia* *acetigenes* HA1243^T^ described in this study, (Figure ST-11A), with 16S rRNA gene identity of 97.4%, suggesting strain HA0445^T^ is a member of the genus *Mengyingia*. The genome of strain HA0445^T^ was sequenced and the NMDC (National Microbiology Data Center) accession number is NMDC60137281. Genome-based analysis showed that the ANI value of genomes of strain HA0445^T^ and *M.* *acetigenes* HA1243^T^ (NMDCN0001F28) is 76.35% and the dDDH estimation is 21.60%. According to the phylogenomic tree (Figure ST-11C), the ANI and dDDH values of genomes between strain HA0445^T^ and phylogenomically neighbored genomes were calculated, and the highest ANI and dDDH values are 76.35% and 21.60%, respectively, between the query genome of *M.* *acetigenes*; and difference in mol% G+C between genomes of strain HA0445^T^ and *M.* *acetigenes* is 6.67; OrthoANI heatmap (Figure ST-11D) shows the phylogenomic status of the corresponding neighbor species based on OrthoANI values. Based on these results, we concluded that the strain HA0445^T^ represents of new species of the genus *Mengyingia*, and the name *Mengyingia* *hominis* sp. nov. is proposed.

Cells are rod-shaped in pairs with pointed ends (2.6 - 6.0 μm long × 0.5 -1.4 μm wide, Figure ST-11B); cells appear in pairs without flagella and non-motile. Growth to stable phase occurs after 24 h incubation in mmGAM medium at 37°C, pH = 7.2. The main fermentation product is acetic acid, and small amounts of butyric acid can also be produced. The G+C content of the type strain HA0445^T^ is around 58.83 mol%. The type strain HA0445^T^ (= CGMCC 1.48218^T^ = KCTC 25696^T^) was isolated from the faeces of rheumatoid arthritis patients.


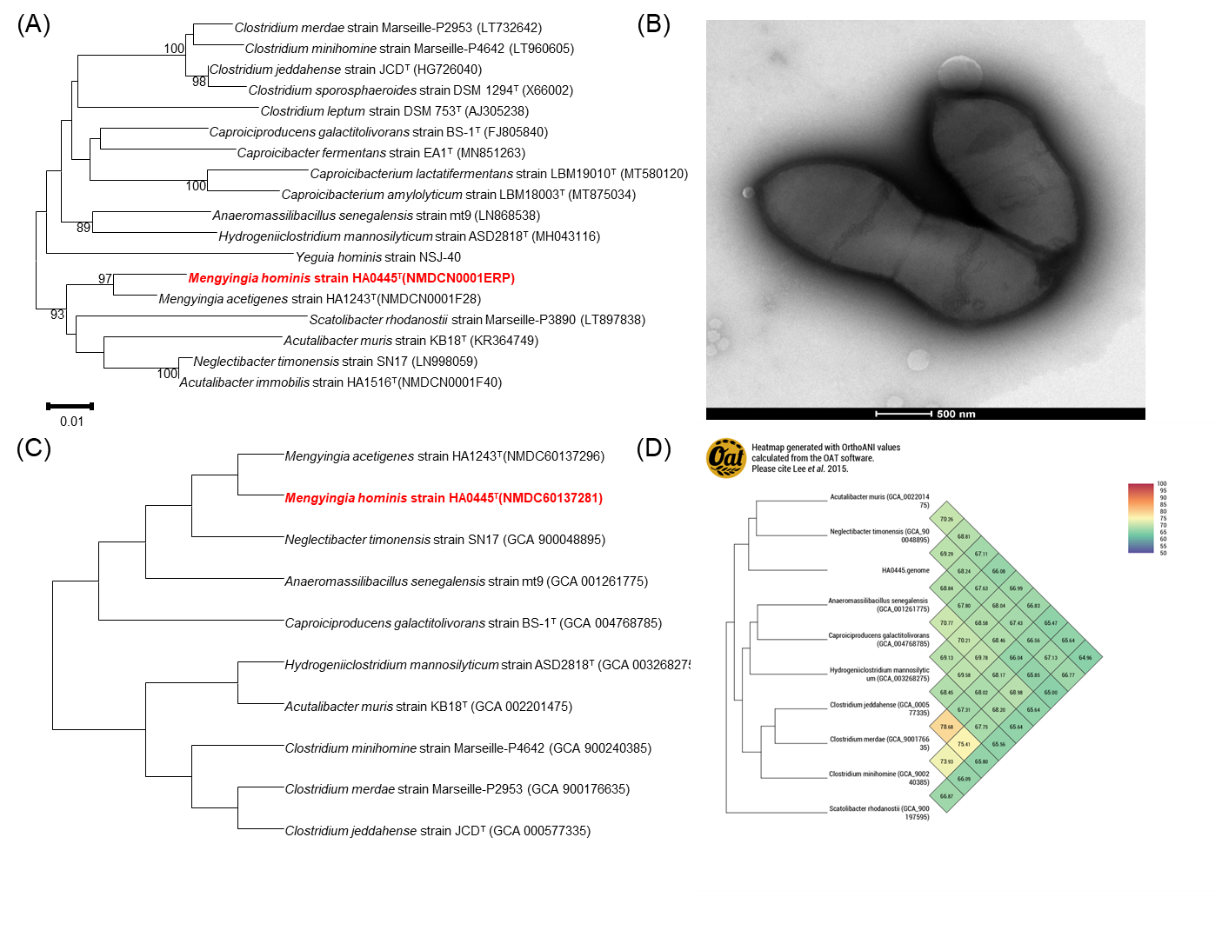


Figure ST-11. The Neighbor-joining phylogenetic tree based on 16S rRNA gene sequences (A) and the cell morphology (B) of strain HA0445^T^. GenBank accession numbers are given in parentheses. Percentages of bootstrap support are shown at branch nodes (values > 70 % are shown). Bootstrap value was 1000. Bar: 0.01 substitutions per nucleotide position. The phylogenomic tree based on genome-sequence-alignment (C) and OrthoANI heatmap (D) of strain HA0445^T^ and its neighbor species. Refseq ID of representative genomes are given in parentheses.

**Taxon 12: *Markus* *hominis* gen. nov. sp. nov.**

**Description of *Markus* gen. nov.** (Mar.k’us. L. masc. n. *Markus*, in honor of the famous microbiologist Markus Göker, whos is a member of the Leibniz Institute DSMZ.)

The closest phylogenetic neighbor of Taxon 12 as represented by strain HA1496^T^ is *Fournierella massiliensis* AT2^T^ (Figure ST-12A). *F. massiliensis* AT2^T^ and HA1496^T^ (16S rRNA sequence accession number in NMDC (National Microbiology Data Center) is NMDCN0001F3C) has 16S rRNA gene identity of 90.77%, and their DNA G+C content is different by 2.28%. The genome of strain HA1496^T^ was sequenced and the NMDC accession number is NMDC60144120. Genome-based analysis on *F. massiliensis* AT2^T^ and type strain HA1496^T^ revealed that the dDDH, ANI and POCP values between *Fournierella massiliensis* AT2^T^ (GCA_004345265) and strain HA1496^T^ are 23.40%, 67.3% and 28.63%, suggesting *F. massiliensis* AT2^T^ and strain HA1496^T^ are from different genera. According to the phylogenomic tree (Figure ST-12C), the ANIs and dDDH values of genomes between strain HA1496^T^ and phylogenomically neighbored genomes were calculated, and the highest ANI and dDDH values are 68.37% and 22.0%, respectively, between the query genome of *Provencibacterium massiliense* Marseille-P2780^T^ (GCA_900169495); and difference in mol% G+C between genomes of strain HA1496^T^ and *P. massiliense* Marseille-P2780^T^ is 0.49; OrthoANI heatmap (Figure ST-12D) shows the phylogenomic status of the corresponding neighbor species based on OrthoANI values. Cells of the strain HA1496^T^ are rod-shaped. The G+C content is around 59.28 mol%. The type species is *Markus* *hominis*.

**Description of *Markus* *hominis* sp. nov.** (ho’mi.nis. L. gen. masc. n. *hominis*, of a human being, referring to the human gut habitat.)

Cells are rod-shaped with square ends (2.0-3.6 μm long × 0.6 μm wide, Figure ST-12B); cells appear singly without flagella and non-motile. Growth to stable phase occurs after 48 h incubation in mGAM medium at 37°C, pH=7.2. The main fermentation product is acetic acid, and small amounts of propionic, isobutyric, butyric, isovaleric and valeric acid can also be produced. The G+C content of the type strain HA1496^T^ is around 59.28 mol%. The type strain HA1496^T^ (= CGMCC 1.48461^T^ = KCTC 25785^T^) was isolated from the faeces of rheumatoid arthritis patients.


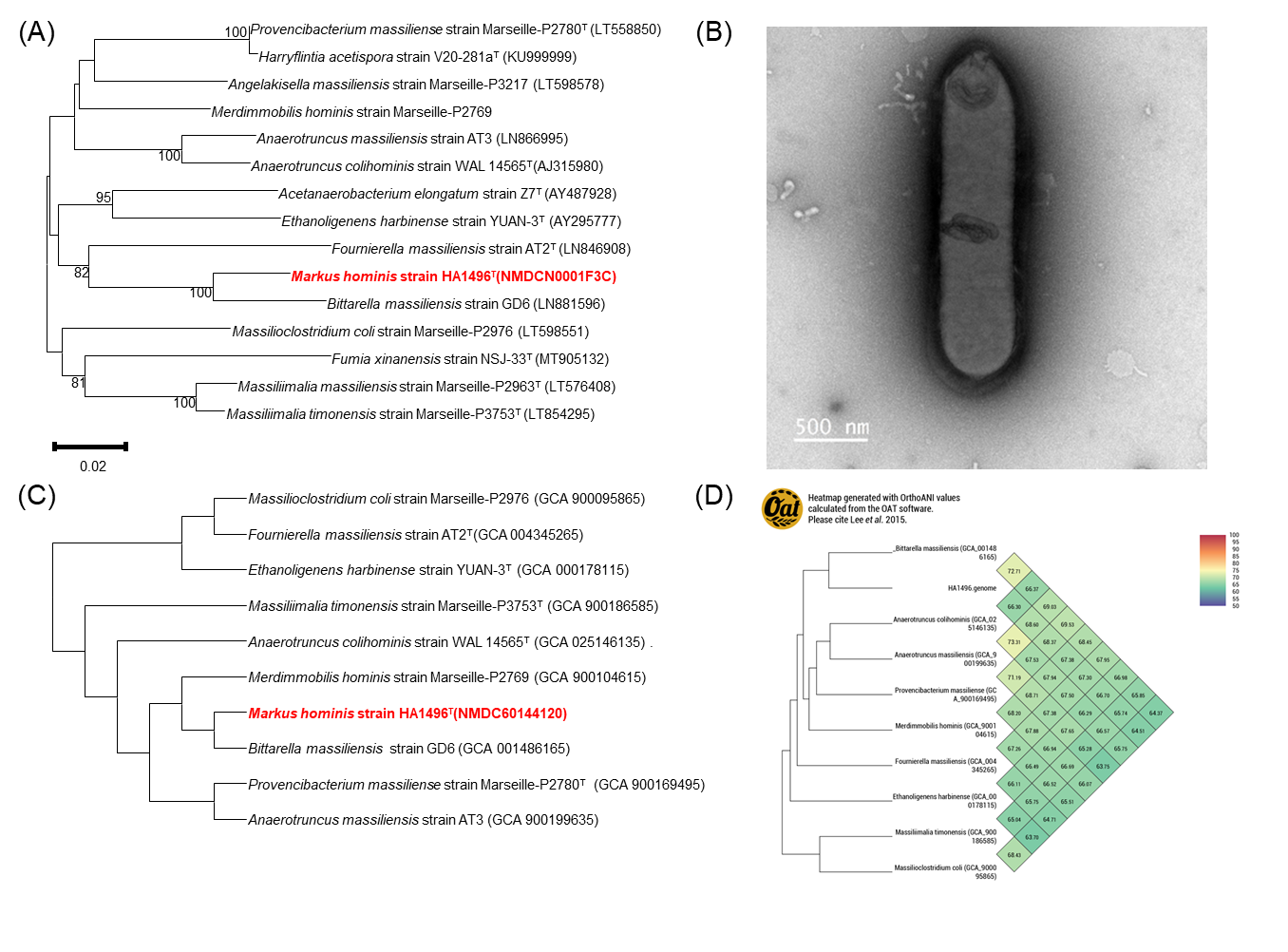


Figure ST-12 The Neighbor-joining phylogenetic tree based on 16S rRNA gene sequences (A) and the cell morphology (B) of strain HA1496^T^. GenBank accession numbers are given in parentheses. Percentages of bootstrap support are shown at branch nodes (values > 70 % are shown). Bootstrap value was 1000. Bar: 0.02 substitutions per nucleotide position. The phylogenomic tree based on genome-sequence-alignment (C) and OrthoANI heatmap (D) of strain HA1496^T^ and its neighbor species. Refseq ID of representative genomes are given in parentheses.

**Taxon 13: *Acutalibacter immobilis* sp. nov.**

**Description of** ***Acutalibacter immobilis* sp. nov.** (im.mo’bi.lis. L. masc. adj. *immobilis*, immovable, motionless, indicating the type strain is non-motile.)

The taxon 13, represented by strain HA1516^T^ (16S rRNA sequence accession number in NMDC is NMDCN0001F40), is phylogenetically closest to *Acutalibacter muris* KB18^T^, with 16S rRNA gene identity of 95.26%. Phylogenetic tree shows that strain HA1516^T^ clusters with other members of the genus *Acutalibacter*, (Figure ST-13A), suggesting strain HA1516^T^ is a member of the genus *Acutalibacter*. The genome of strain HA1516^T^ was sequenced and the NMDC accession number is NMDC60137303. Genome-based analysis showed that the ANI value of genomes of strain HA1516^T^ and *A. muris* KB18^T^ (GCA_002201475) is 70.13% and the dDDH estimation is 20.9%. According to the phylogenomic tree (Figure ST-13C), the ANI and dDDH values of genomes between strain HA1516^T^ and phylogenomically neighbored genomes were calculated, and the highest ANI and dDDH values are 70.13% and 20.9% , respectively, between the query genome of *Acutalibacter muris* KB18^T^ (GCA_002201475); and difference in mol% G+C between genomes of strain HA1516^T^ and *A. muris* KB18^T^ is 1.85; OrthoANI heatmap (Figure ST-13D) shows the phylogenomic status of the corresponding neighbor species based on OrthoANI values. Based on these results, we concluded that the strain HA1516^T^ represents of new species of the genus *Acutalibacter*, and the name *Acutalibacter immobilis* sp. nov. is proposed.

Cells are short rod-shaped with oval ends (0.8 - 3.2 μm long × 0.8 - 1.0 μm wide, Figure ST-13B); cells appear in dividing pairs without flagella and non-motile. Growth to stable phase occurs after 24 h incubation in mmGAM medium at 37 °C, pH = 7.2. No significant fermentation products detected. The G+C content of the type strain HA1516^T^ is around 52.79 mol%. The type strain HA1516^T^ (= CGMCC 1.17975^T^ = KCTC 25684^T^) was isolated from the faeces of rheumatoid arthritis patients.


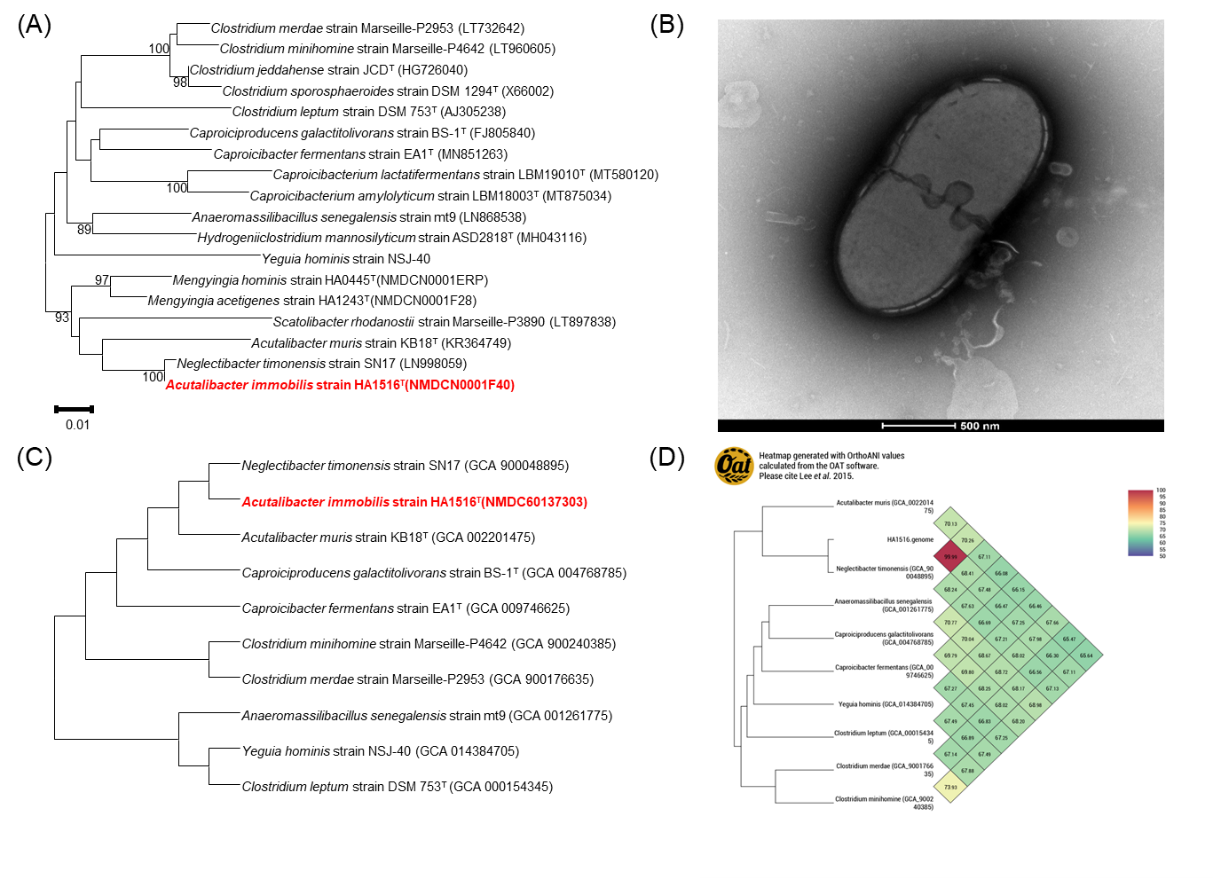


Figure ST-13. The Neighbor-joining phylogenetic tree based on 16S rRNA gene sequences (A) and the cell morphology (B) of strain HA1516^T^. GenBank accession numbers are given in parentheses. Percentages of bootstrap support are shown at branch nodes (values > 70 % are shown). Bootstrap value was 1000. Bar: 0.01 substitutions per nucleotide position. The phylogenomic tree based on genome-sequence-alignment (C) and OrthoANI heatmap (D) of strain HA1516^T^ and its neighbor species. Refseq ID of representative genomes are given in parentheses. **Taxon 14: *Varibaculum hominis* sp. nov**.

**Description of *Varibaculum hominis* sp. nov.** (ho’mi.nis. L. gen. neut. n. *hominis*, of a human being, indicating that the type strain was isolated from a human.)

The Taxon 14, represented by strain HA1244^T^ (16S rRNA sequence accession number in NMDC is NMDCN0001F29), is phylogenetically closest to *Varibaculum cambriense* CCUG 44998^T^, with 16S rRNA gene identity of 98.62%. Phylogenetic tree shows that strain HA1244^T^ clusters with other members of the genus *Varibaculum,* (Figure ST-14A), suggesting strain HA1244^T^ is a member of the genus *Varibaculum.* The genome of strain HA1244^T^ was sequenced and the NMDC (National Microbiology Data Center) accession number is NMDC60137297. Genome-based analysis showed that the ANI value of genomes of strain HA1244^T^ and *V. cambriense* CCUG 44998^T^ (GCA_000420065) is 86.83% and the dDDH estimation is 31.8%. According to the phylogenomic tree (Figure ST-14C), the ANIs and dDDH values of genomes between strain HA1244^T^ and phylogenomically neighbored genomes were calculated, and the highest ANI and dDDH values are 86.83% and 31.8%, respectively, between the query genome of *V. cambriense* CCUG 44998^T^; and difference in mol% G+C between genomes of strain HA1244^T^ and *V. cambriense* CCUG 44998^T^ is 0.87; OrthoANI heatmap (Figure ST-14D) shows the phylogenomic status of the corresponding neighbor species based on OrthoANI values. Based on these results, we concluded that the strain HA1244^T^ represents of new species of the genus *Varibaculum*, and the name *Varibaculum* *hominis* sp. nov. is proposed.

Cells are short rod-shaped (1.5- 3.8 μm long × 0.6 μm wide, Figure ST-14B) and have no flagella and non-motile. Growth to stable phase occurs after 24 h incubation in mmGAM medium at 37°C, pH = 7.2. No significant fermentation products detected. The G+C content of the type strain HA1244^T^ is around 52.56 mol%. The type strain HA1244^T^ (= CGMCC 1.48426^T^ = KCTC 25715^T^) was isolated from the faeces of rheumatoid arthritis patients.


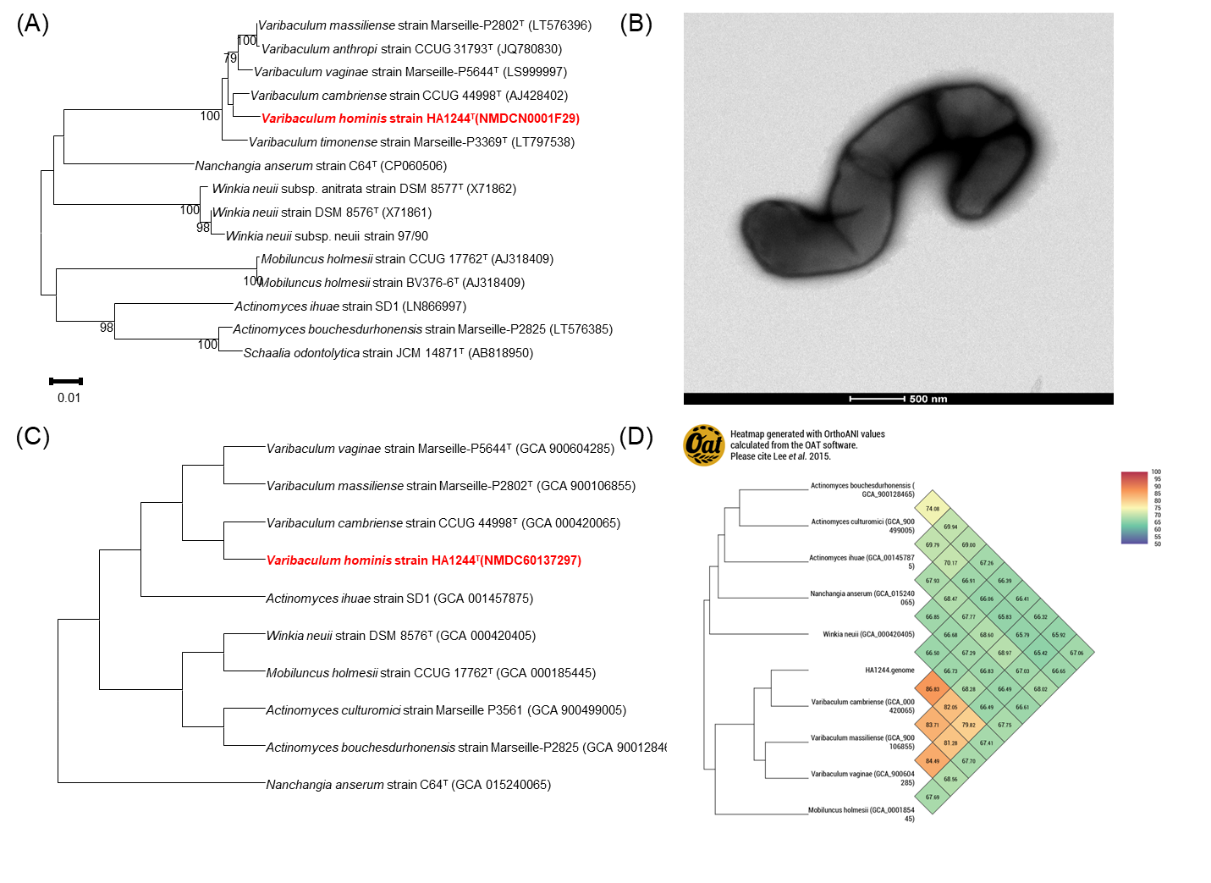


Figure ST-14. The Neighbor-joining phylogenetic tree based on 16S rRNA gene sequences (A) and the cell morphology (B) of strain HA1244^T^. GenBank accession numbers are given in parentheses. Percentages of bootstrap support are shown at branch nodes (values > 70 % are shown). Bootstrap value was 1000. Bar: 0.01 substitutions per nucleotide position. The phylogenomic tree based on genome-sequence-alignment (C) and OrthoANI heatmap (D) of strain HA1244^T^ and its neighbor species. Refseq ID of representative genomes are given in parentheses.

**Taxon 15: *Senegalimassilia arthritidis* sp. nov**.

**Description of *Senegalimassilia arthritidis* sp. nov.** (ar.thri’ti.dis. Gr. fem. n. *arthron*, joint; N.L. fem. n. suff. *-itis*, inflammation; N.L. gen. fem. n. *arthritidis*, of arthritis, denoting the type strain was isolated from the faeces of a rheumatoid arthritis patient.)

The Taxon 15, represented by strain HA0643^T^ (16S rRNA sequence accession number in NMDC is NMDCN0001EVG), is phylogenetically closest to *Senegalimassilia anaerobia* JC110^T^, with 16S rRNA gene identity of 97.32%. Phylogenetic tree shows that strain HA0643^T^ clusters with other members of the genus *Senegalimassilia,* (Figure ST-15A), suggesting strain HA0643^T^ is a member of the genus *Senegalimassilia.* The genome of strain HA0643^T^ was sequenced and the NMDC (National Microbiology Data Center) accession number is NMDC60137291. Genome-based analysis showed that the ANI value of genomes of strain HA0643^T^ and *S. anaerobia* JC110^T^ (GCA_000236865) is 95.13% and the dDDH estimation is 62.9%. According to the phylogenomic tree (Figure ST-15C), the ANIs and dDDH values of genomes between strain HA0643^T^ and phylogenomically neighbored genomes were calculated, and the highest ANI and dDDH values are 95.13% and 62.9%, respectively, between the query genome of *S. anaerobia* JC110^T^; and difference in mol% G+C between genomes of strain HA0643^T^ and *S. anaerobia* JC110^T^ is 0.01; OrthoANI heatmap (Figure ST-15D) shows the phylogenomic status of the corresponding neighbor species based on OrthoANI values. Based on these results, we concluded that the strain HA0643^T^ represents of new species of the genus *Senegalimassilia*, and the name *Senegalimassilia arthritidis* sp. nov. is proposed.

Cells are coccobacillary with rounded ends (1.5 -2.2 μm long × 0.6 μm wide, Figure ST-15B); cells appear singly without flagella and non-motile. Growth to stable phase occurs after 48 h incubation in mmGAM medium at 37°C, pH = 7.2. No significant fermentation products detected. The G+C content of the type strain HA0643^T^ is around 62.05 mol%. The type strain HA0643^T^ (= CGMCC 1.48328^T^ = KCTC 25752^T^) was isolated from the faeces of rheumatoid arthritis patients.


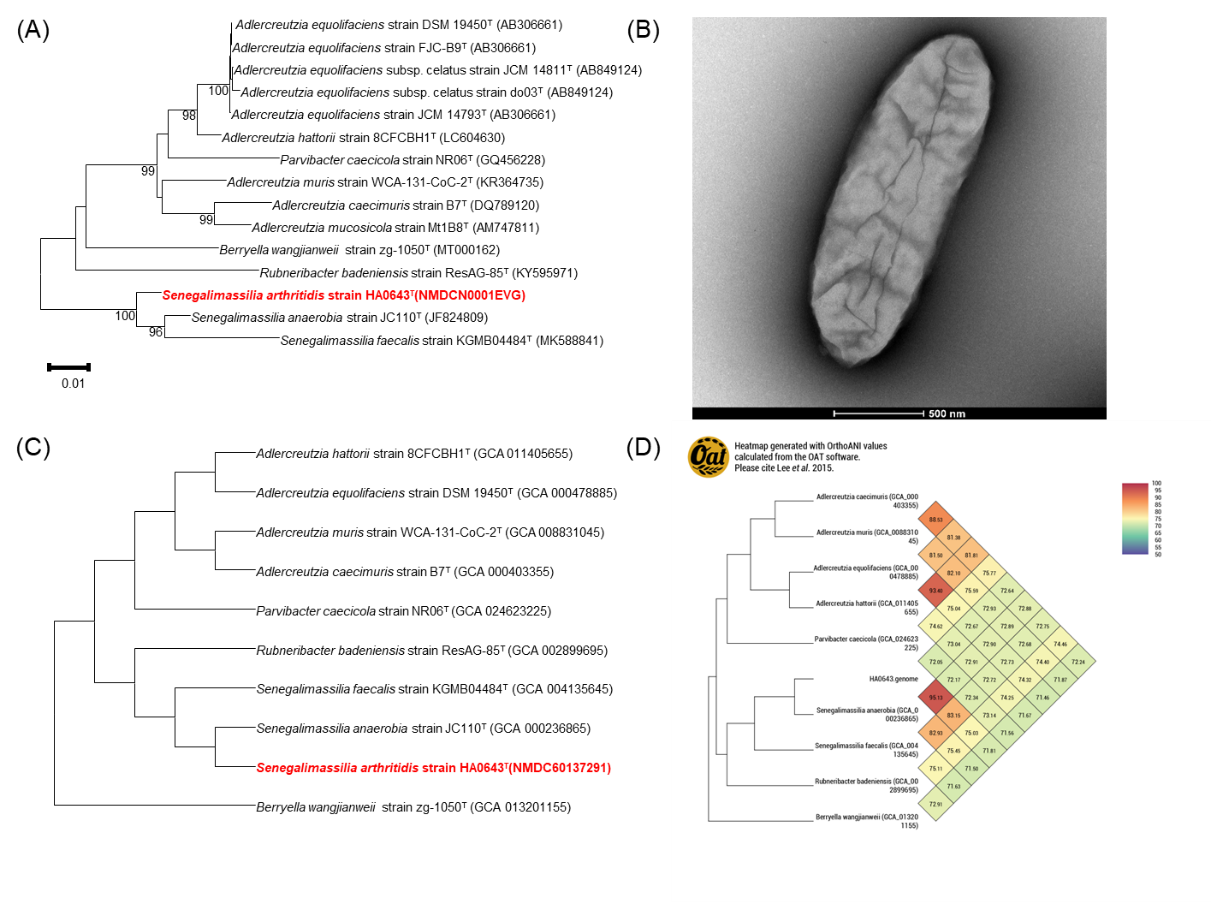


Figure ST-15. The Neighbor-joining phylogenetic tree based on 16S rRNA gene sequences (A) and the cell morphology (B) of strain HA0643^T^. GenBank accession numbers are given in parentheses. Percentages of bootstrap support are shown at branch nodes (values > 70 % are shown). Bootstrap value was 1000. Bar: 0.01 substitutions per nucleotide position. The phylogenomic tree based on genome-sequence-alignment (C) and OrthoANI heatmap (D) of strain HA0643^T^ and its neighbor species. Refseq ID of representative genomes are given in parentheses. **Taxon 16: *Enterococcus immobilis* sp. nov**.

**Description of *Enterococcus immobilis* sp. nov.** (im.mo’bi.lis. L. masc. adj. *immobilis*, non-motile, indicating the non-motility of the type strain of the species.)

The taxon 16 represented by strain HA0446^T^ (16S rRNA sequence accession number in NMDC is NMDCN0001ERQ), is phylogenetically closest to *Enterococcus diestrammenae* ORL-24^T^, with 16S rRNA gene identity of 98.64%. Phylogenetic tree shows that strain HA0446^T^ clusters with other members of the genus *Enterococcus*, (Figure ST-16A), suggesting strain HA0446^T^ is a member of the genus *Enterococcus*. The genome of strain HA0446^T^ was sequenced and the NMDC (National Microbiology Data Center) accession number is NMDC60137282. Genome-based analysis showed that the ANI value of genomes of strain HA0446^T^ and *E. diestrammenae* ORL-24^T^ (GCA_009933255) is 75.73% and the dDDH estimation is 22.60%. According to the phylogenomic tree (Figure ST-16C), the ANI and dDDH values of genomes between strain HA0446^T^ and phylogenomically neighbored genomes were calculated, and the highest ANI and dDDH values are 75.73% and 22.60%, respectively, between the query genome of *E. diestrammenae* ORL-24^T^; and difference in mol% G+C between genomes of strain HA0446^T^ and *E. diestrammenae* ORL-24^T^ is 0.74; OrthoANI heatmap (Figure ST-16D) shows the phylogenomic status of the corresponding neighbor species based on OrthoANI values. Based on these results, we concluded that the strain HA0446^T^ represents of new species of the genus *Enterococcus*, and the name *Enterococcus immobilis* sp. nov. is proposed

Cells are oval-shaped (0.7 - 1.2 μm long × 0.6 μm wide, Figure ST-16B); cells appear in (dividing) pairs without flagella and non-motile. Growth to stable phase occurs after 24 h incubation in mmGAM medium at 37°C, pH = 7.2. The main fermentation product is acetic acid, and small amounts of propionic, isobutyric, butyric, isovaleric and valeric acid can also be produced. The G+C content of the type strain HA0446^T^ is around 43.75 mol%. The type strain HA0446^T^ (= CGMCC 1.48219^T^ = KCTC 25678^T^) was isolated from the faeces of rheumatoid arthritis patients.


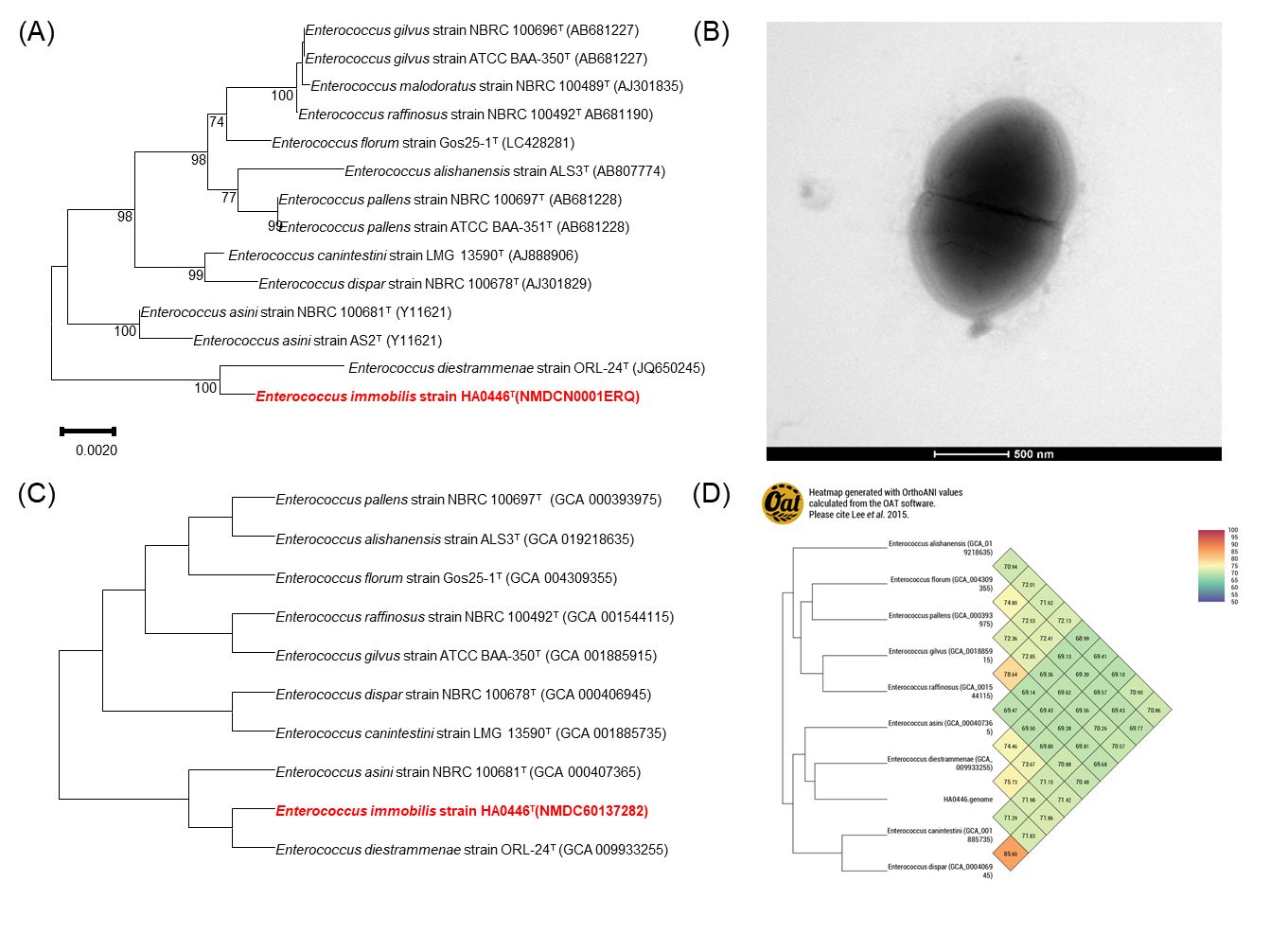


Figure ST-16. The Neighbor-joining phylogenetic tree based on 16S rRNA gene sequences (A) and the cell morphology (B) of strain HA0446^T^. GenBank accession numbers are given in parentheses. Percentages of bootstrap support are shown at branch nodes (values > 70 % are shown). Bootstrap value was 1000. Bar: 0.0020 substitutions per nucleotide position. The phylogenomic tree based on genome-sequence-alignment (C) and OrthoANI heatmap (D) of strain HA0446^T^ and its neighbor species. Refseq ID of representative genomes are given in parentheses. **Taxon 17: *Streptococcus immobilis* sp. nov**.

**Description of *Streptococcus immobilis* sp. nov.** (im.mo’bi.lis. L. masc. adj. *immobilis*, non-motile, indicating the non-motility of the type strain of the species.)

The taxon 17 represented by strain HA0527^T^ (16S rRNA sequence accession number in NMDC is NMDCN0001ESP), is phylogenetically closest to *Streptococcus parasanguinis* ATCC 15912^T^, with 16S rRNA gene identity of 98.38%. Phylogenetic tree shows that strain HA0527^T^ clusters with other members of the genus *Streptococcus*, (Figure ST-17A), suggesting strain HA0527^T^ is a member of the genus *Streptococcus*. The genome of strain HA0527^T^ was sequenced and the NMDC (National Microbiology Data Center) accession number is NMDC60137284. Genome-based analysis showed that the ANI value of genomes of strain HA0527^T^ and *S. parasanguinis* ATCC 15912^T^ (GCA_000164675) is 93.79% and the dDDH estimation is 54.80%. According to the phylogenomic tree (Figure ST-17C), the ANI and dDDH values of genomes between strain HA0527^T^ and phylogenomically neighbored genomes were calculated, and the highest ANI and dDDH values are 93.79% and 54.80%, respectively, between the query genome of *S. parasanguinis* ATCC 15912^T^; and difference in mol% G+C between genomes of strain HA0527^T^ and *S. parasanguinis* ATCC 15912^T^ is 0.12; OrthoANI heatmap (Figure ST-17D) shows the phylogenomic status of the corresponding neighbor species based on OrthoANI values. Based on these results, we concluded that the strain HA0527^T^ represents of new species of the genus *Streptococcus*, and the name *Streptococcus immobilis* sp. nov. is proposed.

Cells are oval-shaped with spiky ends (0.65 - 0.8 μm long × 0.5 μm wide, Figure ST-17B); cells appear in dividing pairs without flagella and non-motile. Growth to stable phase occurs after 48 h incubation in mmGAM medium at 37°C, pH = 7.2. The main fermentation product is acetic acid, and small amounts of butyric acid can also be produced. The G+C content of the type strain HA0527^T^ is around 41.85 mol%. The type strain HA0527^T^ (= CGMCC 1.48250^T^ = KCTC 25679^T^) was isolated from the faeces of rheumatoid arthritis patients.


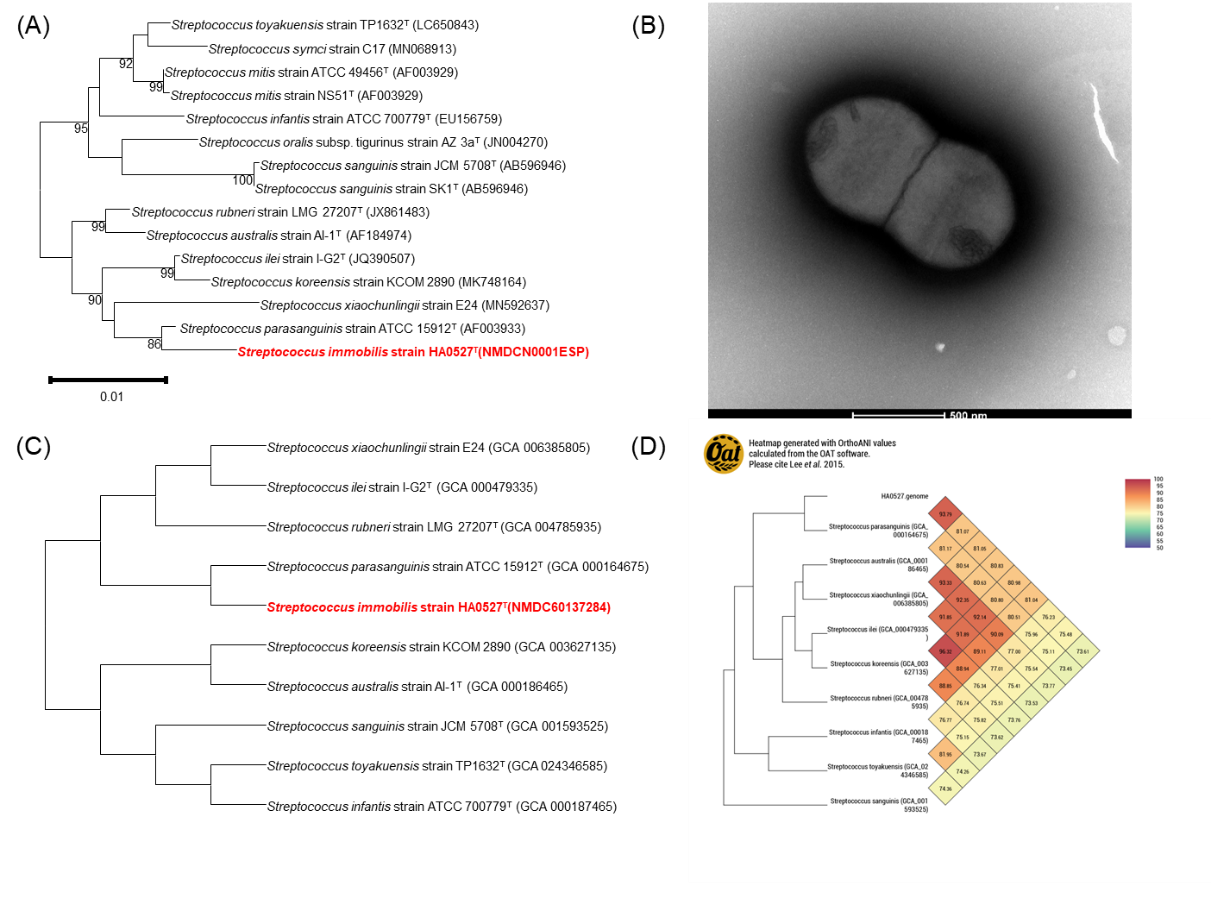


Figure ST-17. The Neighbor-joining phylogenetic tree based on 16S rRNA gene sequences (A) and the cell morphology (B) of strain HA0527^T^. GenBank accession numbers are given in parentheses. Percentages of bootstrap support are shown at branch nodes (values > 70 % are shown). Bootstrap value was 1000. Bar: 0.01 substitutions per nucleotide position. The phylogenomic tree based on genome-sequence-alignment (C) and OrthoANI heatmap (D) of strain HA0527^T^ and its neighbor species. Refseq ID of representative genomes are given in parentheses. **Taxon 18: *Faecalibacillus hominis* sp. nov**.

**Description of *Faecalibacillus hominis* sp. nov.** (ho’mi.nis. L. gen. masc. n. *hominis*, of a human being, indicating that the type strain was isolated from a human)

The taxon 18, represented by strain HA0003^T^ (16S rRNA sequence accession number in NMDC is NMDCN0001EL2), is phylogenetically closest to *Faecalibacillus* *intestinalis* SNUG30099^T^, with 16S rRNA gene identity of 95.42%. Phylogenetic tree shows that strain HA0003^T^ clusters with other members of the genus *Faecalibacillus*, (Figure ST-18A), suggesting strain HA0003^T^ is a member of the genus *Faecalibacillus*. The genome of strain HA0003^T^ was sequenced and the NMDC (National Microbiology Data Center) accession number is NMDC60137266. Genome-based analysis showed that the ANI value of genomes of strain HA0003^T^ and *F. intestinalis* SNUG30099^T^ (GCA_003024685) is 76.76% and the dDDH estimation is 21.40%. According to the phylogenomic tree (Figure ST-18C), the ANIs and dDDH values of genomes between strain HA0003^T^ and phylogenomically neighbored genomes were calculated, and the highest ANI and dDDH values are 76.76% and 21.4%, respectively, between the query genome of *F. intestinalis* SNUG30099^T^; and difference in mol% G+C between genomes of strain HA0003^T^ and *F. intestinalis* SNUG30099^T^ is 1.1; OrthoANI heatmap (Figure ST-18D) shows the phylogenomic status of the corresponding neighbor species based on OrthoANI values. Based on these results, we concluded that the strain HA0003^T^ represents of new species of the genus *Faecalibacillus*, and the name *Faecalibacillus hominis* sp. nov. is proposed.

Cells are short rod-shaped with square ends (1.2 - 3.0 μm long × 0.6 μm wide, Figure ST-18B); cells appear in dividing pairs without flagella and non-motile. Growth to stable phase occurs after 24 h incubation in mmGAM medium at 37°C, pH = 7.2. The main fermentation product is acetic acid, and small amounts of propionic, isobutyric, butyric, isovaleric and valeric acid can also be produced. The G+C content of the type strain HA0003^T^ is around 30.34 mol%. The type strain HA0003^T^ (= CGMCC 1.48003^T^ = KCTC 25672^T^) was isolated from the faeces of rheumatoid arthritis patients.


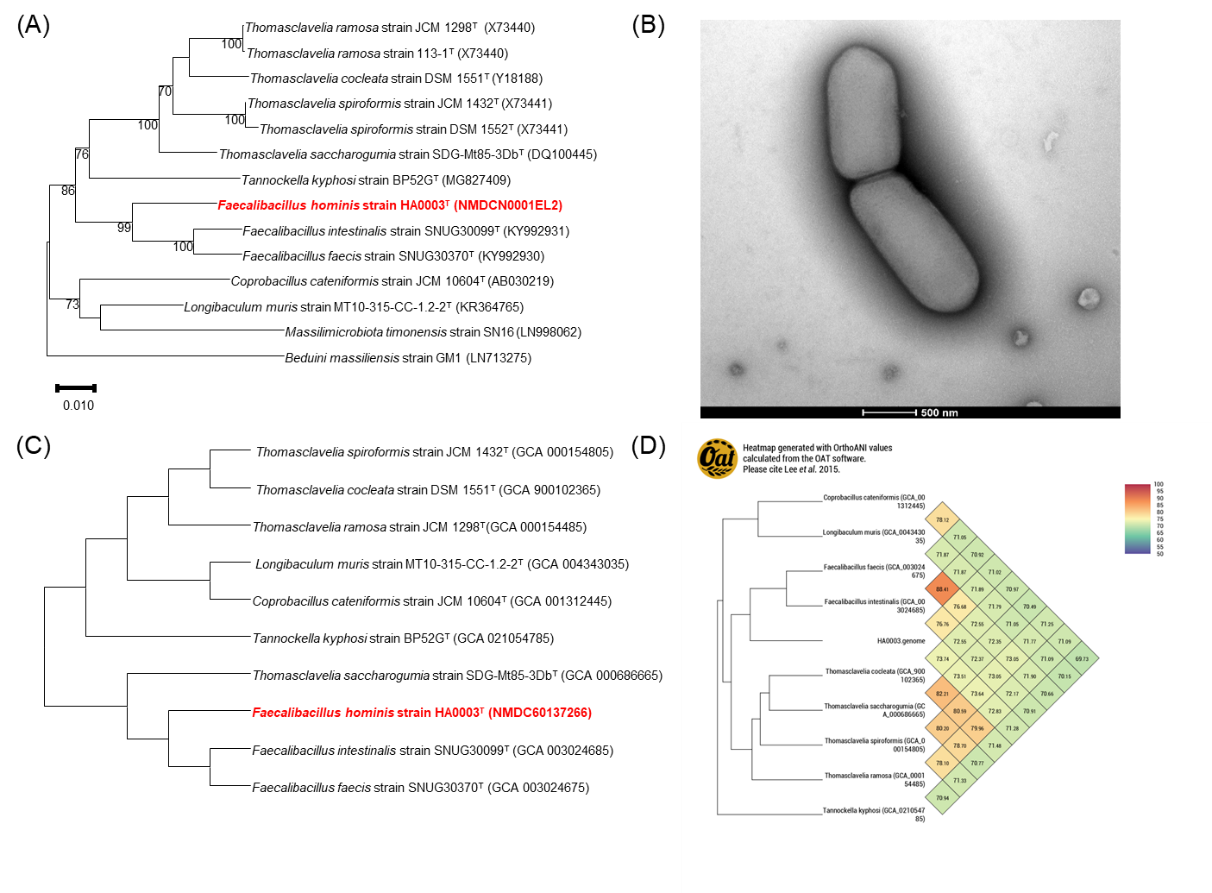


Figure ST-18. The Neighbor-joining phylogenetic tree based on 16S rRNA gene sequences (A) and the cell morphology (B) of strain HA0003^T^. GenBank accession numbers are given in parentheses. Percentages of bootstrap support are shown at branch nodes (values > 70 % are shown). Bootstrap value was 1000. Bar: 0.01 substitutions per nucleotide position. The phylogenomic tree based on genome-sequence-alignment (C) and OrthoANI heatmap (D) of strain HA0003^T^ and its neighbor species. Refseq ID of representative genomes are given in parentheses. **Taxon 19: *Eubacterium acidiformans* sp. nov**.

**Description of *Eubacterium acidiformans* sp. nov.** (a.ci.di.for’mans. L. neut. adj. *acidum*, an acid; from L. masc. adj. *acidus*, sour; L. pres. part. *formans*, forming; N.L. part. adj. *acidiformans*, acid-forming, indicating the strain produces acids.)

The taxon 19, represented by strain HA0433^T^ (16S rRNA sequence accession number in NMDC is NMDCN0001ERD), is phylogenetically closest to *Eubacterium ventriosum* ATCC 27560^T^, with 16S rRNA gene identity of 94.70%. Phylogenetic tree shows that strain HA0433^T^ clusters with other members of the genus *Eubacterium*, (Figure ST-19A), suggesting strain HA0433^T^ is a member of the genus *Eubacterium*. The genome of strain HA0433^T^ was sequenced and the NMDC accession number is NMDC60137273. Genome-based analysis showed that the ANI value of genomes of strain HA0433^T^ and *E. ventriosum* ATCC 27560^T^ (GCA_025150745) is 75.2% and the dDDH estimation is 26.0%. According to the phylogenomic tree (Figure ST-19C), the ANIs and dDDH values of genomes between strain HA0433^T^ and phylogenomically neighbored genomes were calculated, and the highest ANI and dDDH values are 75.2% and 26.0%, respectively, between the query genome of *E. ventriosum* ATCC 27560^T^; and difference in mol% G+C between genomes of strain HA0433^T^ and *E. ventriosum* ATCC 27560^T^ is 1.93; OrthoANI heatmap (Figure ST-19D) shows the phylogenomic status of the corresponding neighbor species based on OrthoANI values. Based on these results, we concluded that the strain HA0433^T^ represents of new species of the genus *Eubacterium*, and the name *Eubacterium acidiformans* sp. nov. is proposed.

Cells are coccobacillary-shaped with rounded ends (1.2 - 1.6 μm long × 0.6 μm wide, Figure ST-19B); cells appear in dividing pairs without flagella and non-motile. Growth to stable phase occurs after 48 h incubation in mmGAM medium at 37°C, pH = 7.2. The main fermentation products are acetic and butyric acid. The G+C content of the type strain HA0433^T^ is around 32.99 mol%. The type strain HA0433^T^ (= CGMCC 1.48206^T^ = KCTC 25713^T^) was isolated from the faeces of rheumatoid arthritis patients.


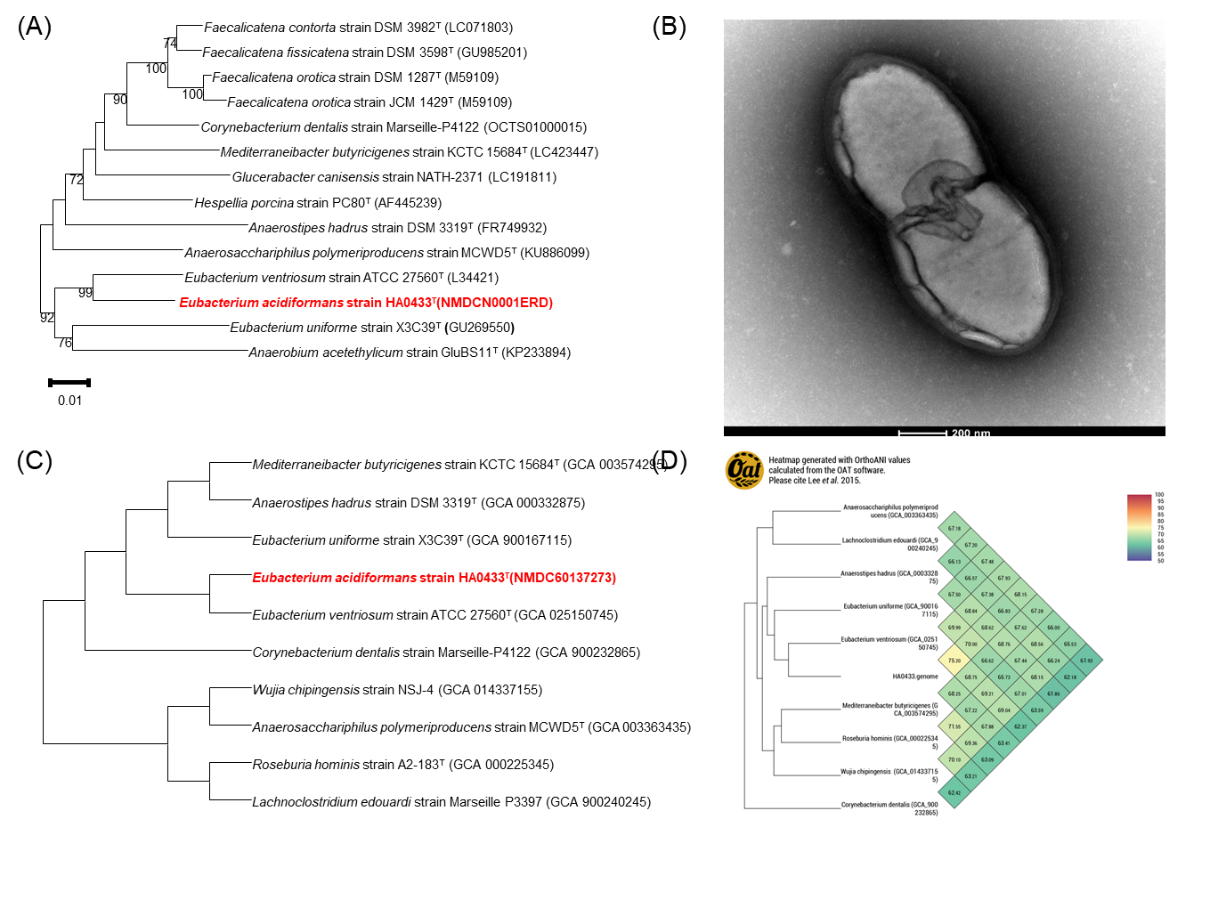


Figure ST-19. The Neighbor-joining phylogenetic tree based on 16S rRNA gene sequences (A) and the cell morphology (B) of strain HA0433^T^. GenBank accession numbers are given in parentheses. Percentages of bootstrap support are shown at branch nodes (values > 70% are shown). Bootstrap value was 1000. Bar: 0.01 substitutions per nucleotide position. The phylogenomic tree based on genome-sequence-alignment (C) and OrthoANI heatmap (D) of strain HA0433^T^ and its neighbor species. Refseq ID of representative genomes are given in parentheses.

**Taxon 20:** ***Blautia* *arthritidis* sp. nov**.

**Description of** ***Blautia arthritidis* sp. nov.** (ar.thri’ti.dis. Gr. fem. n. *arthron*, joint; N.L. fem. n. suff. *-itis*, inflammation; N.L. gen. fem. n. *arthritidis*, of arthritis, denoting the type strain was isolated from the faeces of rheumatoid arthritis patients.)

The taxon 20, represented by strain HA0067^T^ (16S rRNA sequence accession number in NMDC is NMDCN0001EMQ), is phylogenetically closest to *Blautia luti* BlnlX^T^, with 16S rRNA gene identity of 97.40%. Phylogenetic tree shows that strain HA0067^T^ clusters with other members of the genus *Blautia*, (Figure ST-20A), suggesting strain HA0067^T^ is a member of the genus *Blautia*. The genome of strain HA0067^T^ was sequenced and the NMDC accession number is NMDC60137271. Genome-based analysis showed that the ANI value of genomes of strain HA0067^T^ and *B*. *luti* BlnlX^T^ (GCA_009707925) is 78.06% and the dDDH estimation is 30.5%. According to the phylogenomic tree (Figure ST-20C), the ANIs and dDDH values of genomes between strain HA0067^T^ and phylogenomically neighbored genomes were calculated, and the highest ANI and dDDH values are 78.06% and 30.5%, respectively, between the query genome of *B*. *luti* BlnlX^T^; and difference in mol% G+C between genomes of strain HA0067^T^ and *B. luti* BlnlX^T^ is 1.31; OrthoANI heatmap (Figure ST-20D) shows the phylogenomic status of the corresponding neighbor species based on OrthoANI values. Based on these results, we concluded that the strain HA0067^T^ represents of new species of the genus *Blautia*, and the name *Blautia arthritidis* sp. nov. is proposed.

Cells are rod-shaped with rounded ends (1.4 - 3.0 μm long × 0.5 - 0.8 μm wide, Figure ST-20B); cells appear in (dividing) pairs without flagella and non-motile. Growth to stable phase occurs after 48 h incubation in mmGAM medium at 37°C, pH = 7.2. The main fermentation product is acetic acid. The G+C content of the type strain HA0067^T^ is 44.17 mol%. The type strain HA0067^T^ (= CGMCC 1.48059^T^ = KCTC 25675^T^) was isolated from the faeces of rheumatoid arthritis patients.


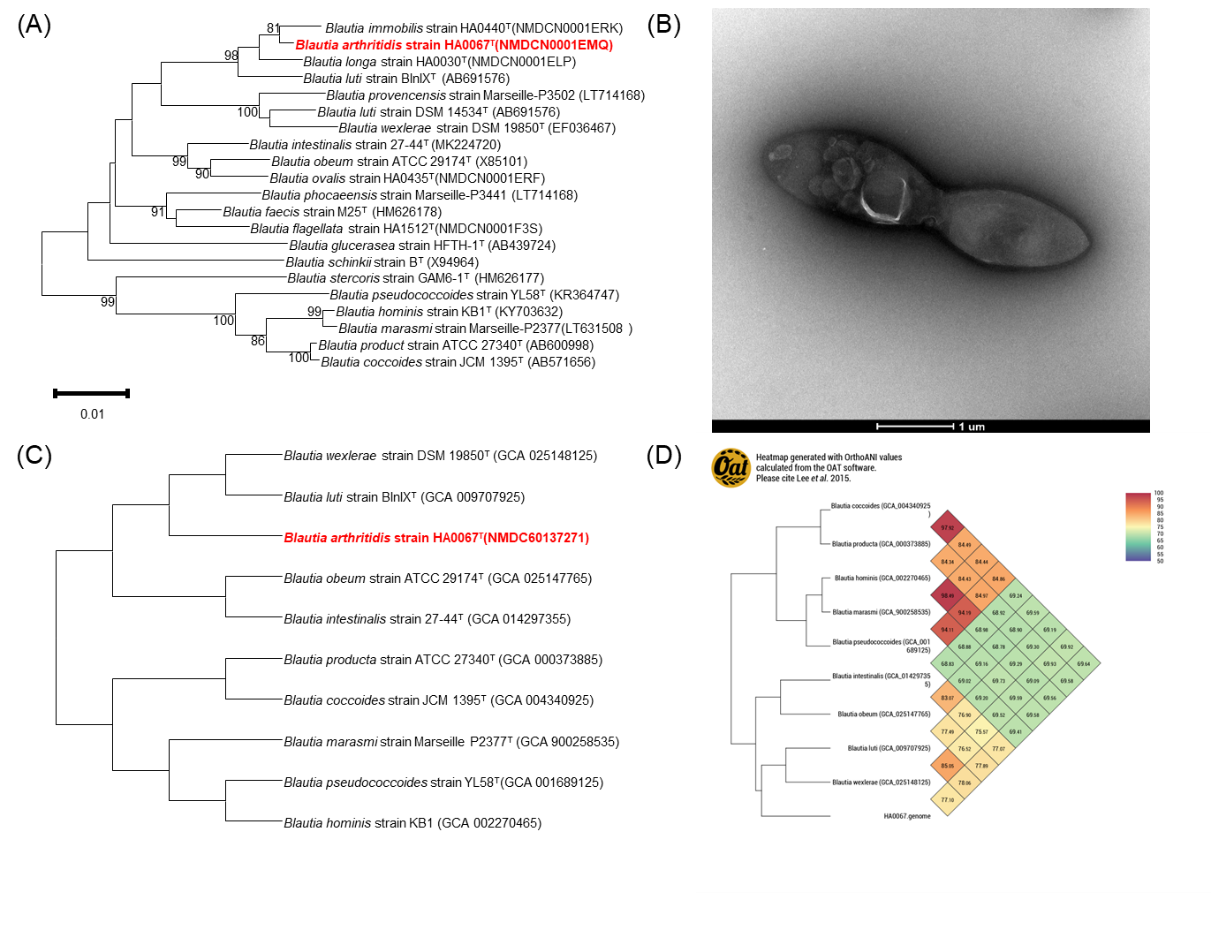


Figure ST-20. The Neighbor-joining phylogenetic tree based on 16S rRNA gene sequences (A) and the cell morphology (B) of strain HA0067^T^. GenBank accession numbers are given in parentheses. Percentages of bootstrap support are shown at branch nodes (values > 70 % are shown). Bootstrap value was 1000. Bar: 0.01 substitutions per nucleotide position. The phylogenomic tree based on genome-sequence-alignment (C) and OrthoANI heatmap (D) of strain HA0067^T^and its neighbor species. Refseq ID of representative genomes are given in parentheses. **Taxon 21: *Blautia flagellata* sp. nov**.

**Description of *Blautia flagellata* sp. nov.** (fla.gel.la’ta. L. fem. n. *flagellum*, a whip; L. fem. adj. suff. *-ata*, suffix denoting provided with; L. fem. part. adj. *flagellata*, flagellated, denoting the type strain has flagellum.)

The taxon 21, represented by strain HA1512^T^ (16S rRNA sequence accession number in NMDC is NMDCN0001F3S), is phylogenetically closest to *Blautia faecis* M25^T^, with 16S rRNA gene identity of 98.42%. Phylogenetic tree shows that strain HA1512^T^ clusters with other members of the genus *Blautia,* (Figure ST-21A), suggesting strain HA1512^T^ is a member of the genus *Blautia.* The genome of strain HA1512^T^ was sequenced and the NMDC (National Microbiology Data Center) accession number is NMDC60137302. Genome-based analysis showed that the ANI value of genomes of strain HA1512^T^ and *B. faecis* M25^T^ (GCA_020554845) is 88.13% and the dDDH estimation is 37.8%. According to the phylogenomic tree (Figure ST-21C), the ANIs and dDDH values of genomes between strain HA1512^T^ and phylogenomically neighbored genomes were calculated, and the highest ANI and dDDH values are 88.13% and 37.8%, respectively, between the query genome of *B. faecis* M25^T^; and difference in mol% G+C between genomes of strain HA1512^T^ and *B. faecis* M25^T^ is 0.76; OrthoANI heatmap (Figure ST-21D) shows the phylogenomic status of the corresponding neighbor species based on OrthoANI values. Based on these results, we concluded that the strain HA1512^T^ represents of new species of the genus *Blautia*, and the name *Blautia flagellata* sp. nov. is proposed.

Cells are rod-shaped with rounded ends (3.0 - 7.0 μm long × 0.5 μm wide, Figure ST-21B); cells appear singly with flagella and motile. Growth to stable phase occurs after 48 h incubation in mmGAM medium at 37°C, pH = 7.2. The main fermentation product is acetic and small amounts of isobutyric acid and butyric acid can also be produced. The G+C content of the type strain HA1512^T^ is around 43.71 mol%. The type strain HA1512^T^ (= CGMCC 1.48477^T^ = KCTC 25718^T^) was isolated from the faeces of rheumatoid arthritis patients.


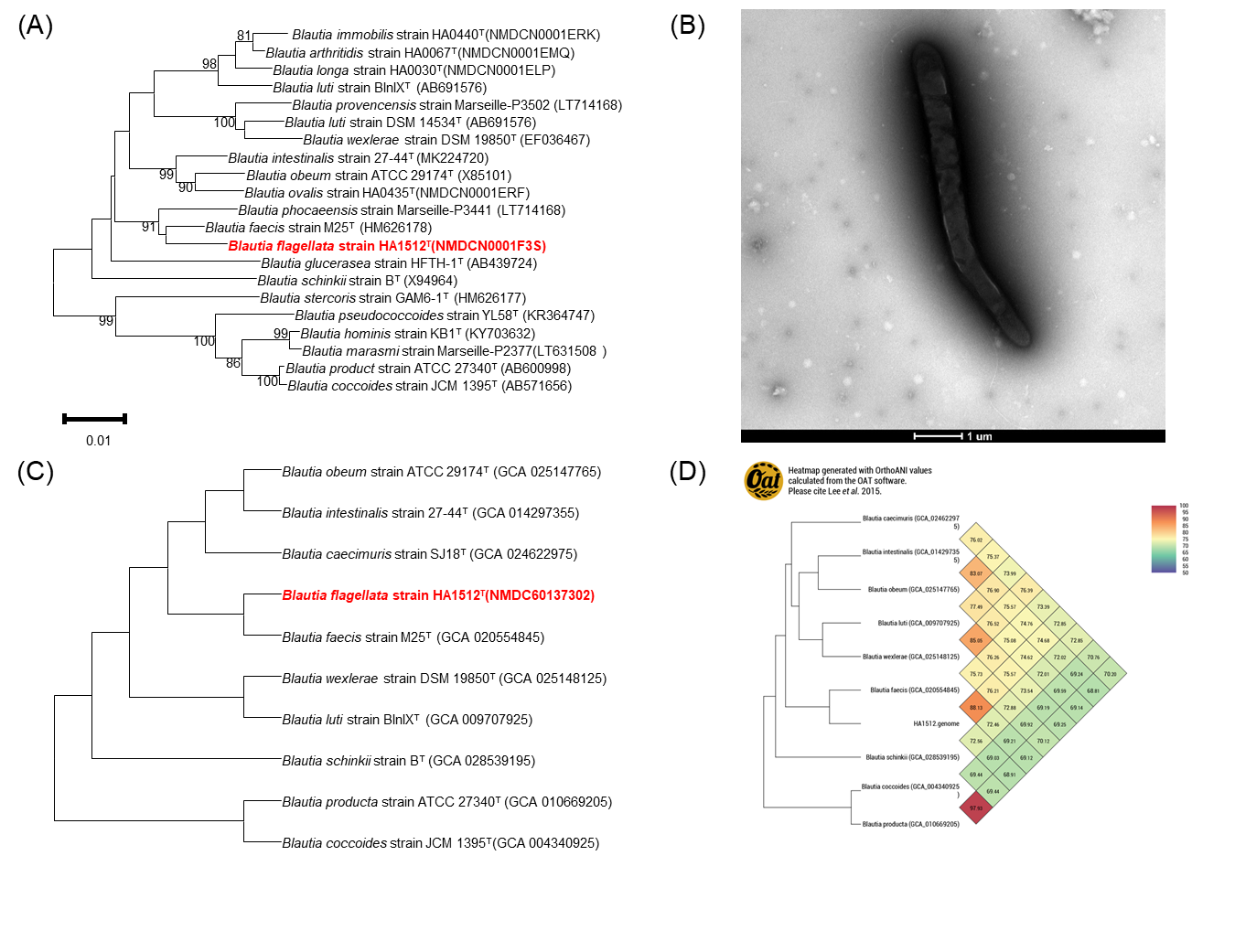


Figure ST-21. The Neighbor-joining phylogenetic tree based on 16S rRNA gene sequences (A) and the cell morphology (B) of strain HA1512^T^. GenBank accession numbers are given in parentheses. Percentages of bootstrap support are shown at branch nodes (values > 70% are shown). Bootstrap value was 1000. Bar: 0.01 substitutions per nucleotide position. The phylogenomic tree based on genome-sequence-alignment (C) and OrthoANI heatmap (D) of strain HA1512^T^ and its neighbor species. Refseq ID of representative genomes are given in parentheses.

**Taxon 22: *Blautia immobilis* sp. nov**.

**Description of *Blautia immobilis* sp. nov.** (im.mo’bi.lis. L. fem. adj. *immobilis*, non-motile, denoting the non-motility of the type strain of the species.)

The taxon 22, represented by strain HA0440^T^ (16S rRNA sequence accession number in NMDC is NMDCN0001ERK), is phylogenetically closest to *Blautia luti* BlnlX^T^, with 16S rRNA gene identity of 97.33%. Phylogenetic tree shows that strain HA0440^T^ clusters with other members of the genus *Blautia*, (Figure ST-22A), suggesting strain HA0440^T^ is a member of the genus *Blautia*. The genome of strain HA0440^T^ was sequenced and the NMDC (National Microbiology Data Center) accession number is NMDC60137277. Genome-based analysis showed that the ANI value of genomes of strain HA0440^T^ and *B. luti* BlnlX^T^ (GCA_009707925) is 77.28% and the dDDH estimation is 29.30%. According to the phylogenomic tree (Figure ST-22C), the ANIs and dDDH values of genomes between strain HA0440^T^ and phylogenomically neighbored genomes were calculated, and the highest ANI and dDDH values are 77.86% and 31.9%, respectively, between the query genome of *Blautia obeum* ATCC 29174^T^ (GCA_025147765); and difference in mol% G+C between genomes of strain HA0440^T^ and *B. obeum* is 2.12; OrthoANI heatmap (Figure ST-22D) shows the phylogenomic status of the corresponding neighbor species based on OrthoANI values. Based on these results, we concluded that the strain HA0440^T^ represents of new species of the genus *Blautia*, and the name *Blautia immobilis* sp. nov. is proposed.

Cells are oval-shaped (1.8 μm long × 1.0 μm wide, Figure ST-22B); cells appear singly without flagella and non-motile. Growth to stable phase occurs after 48 h incubation in mmGAM medium at 37°C, pH = 7.2. The main fermentation product is acetic, propionic acid and small amounts of isobutyric, butyric, isovaleric and valeric acid can also be produced. The G+C content of the type strain HA0440^T^ is around 43.75 mol%. The type strain HA0440^T^ (= CGMCC 1.48213^T^ = KCTC 25742^T^) was isolated from the faeces of rheumatoid arthritis patients.


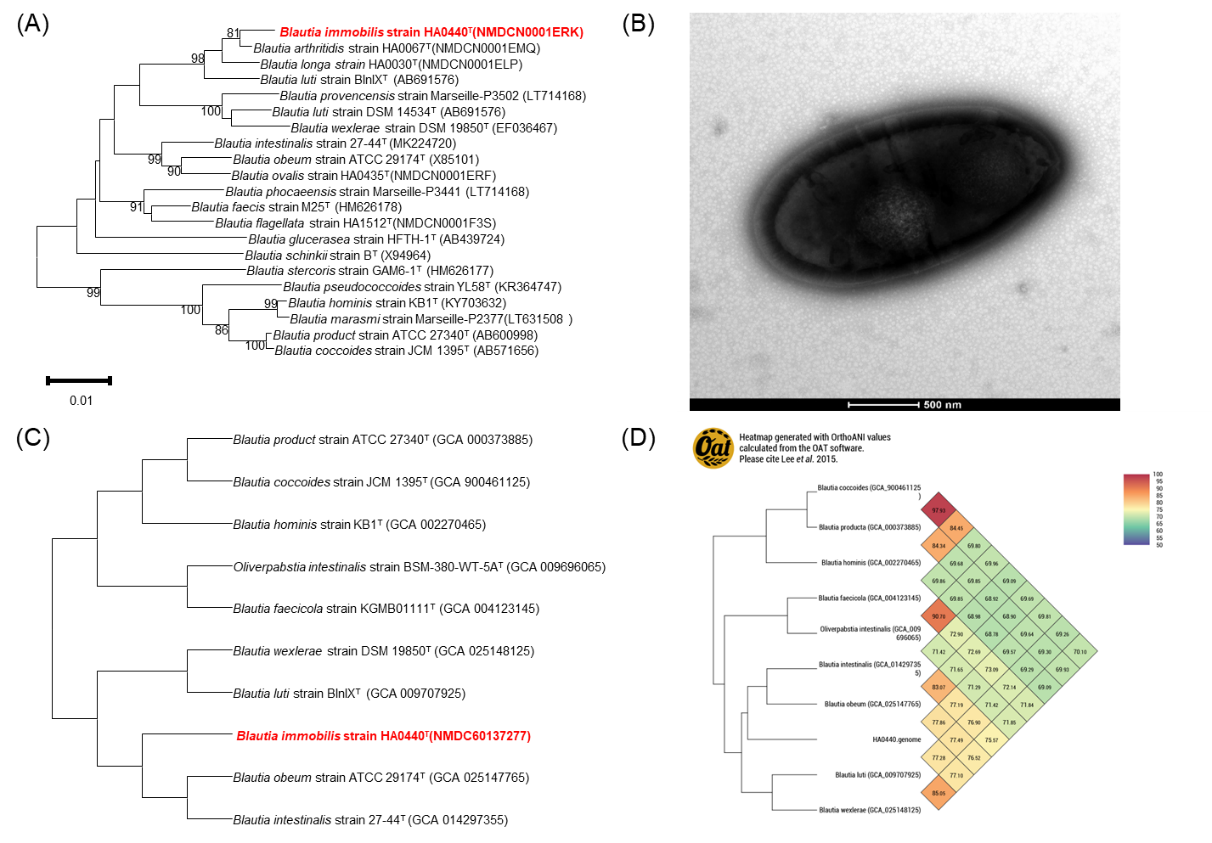


Figure ST-22. The Neighbor-joining phylogenetic tree based on 16S rRNA gene sequences (A) and the cell morphology (B) of strain HA0440^T^. GenBank accession numbers are given in parentheses. Percentages of bootstrap support are shown at branch nodes (values >70% are shown). Bootstrap value was 1000. Bar: 0.01 substitutions per nucleotide position. The phylogenomic tree based on genome-sequence-alignment (C) and OrthoANI heatmap (D) of strain HA0440^T^ and its neighbor species. Refseq ID of representative genomes are given in parentheses. **Taxon 23: *Blautia longa* sp. nov.**

**Description of *Blautia longa* sp. nov.** (lon’ga. L. fem. adj. *longa*, long in shape, referring to the cell shape of the type strain.)

The taxon 23, represented by strain HA0030^T^ (16S rRNA sequence accession number in NMDC is NMDCN0001ELP), is phylogenetically closest to *Blautia luti* BlnlX^T^ (Figure ST-23A), with 16S rRNA gene identity of 98.24%. Phylogenetic tree shows that strain H0030^T^ clusters with other members of the genus *Blautia,* (Figure ST-23A), suggesting strain HA0030^T^ is a member of the genus *Blautia.* The genome of strain HA0030^T^ was sequenced and the NMDC (National Microbiology Data Center) accession number is NMDC60137269. Genome-based analysis showed that the ANI value of genomes of strain HA0030^T^ and *B. luti* BlnlX^T^ (GCA_009707925) is 77.30% and the dDDH estimation is 28.9%. According to the phylogenomic tree (Figure ST-23C), the ANIs and dDDH values of genomes between strain HA0030^T^ and phylogenomically neighbored genomes were calculated, and the highest ANI and dDDH values are 77.3% and 28.90%, respectively, between the query genome of *B. luti* BlnlX^T^; and difference in mol% G+C between genomes of strain HA0030^T^ and *B. luti* BlnlX^T^ is 1.4; OrthoANI heatmap (Figure ST-23D) shows the phylogenomic status of the corresponding neighbor species based on OrthoANI values. Based on these results, we concluded that the strain HA0030^T^ represents of new species of the genus *Blautia*, and the name *Blautia longa* sp. nov. is proposed.

Cells are long rod-shaped with oval ends (1.2 - 6.0 μm long × 0.5 μm wide, Figure ST-23B); cells appear singly without flagella and motile. Growth to stable phase occurs after 48 h incubation in mmGAM medium at 37°C, pH = 7.2. The main fermentation products are acetic and butyric acid. The G+C content of the type strain HA0030^T^ is around 44.26 mol%. The type strain HA0030^T^ (= CGMCC 1.48026^T^ = KCTC 25674^T^) was isolated from the faeces of rheumatoid arthritis patients.


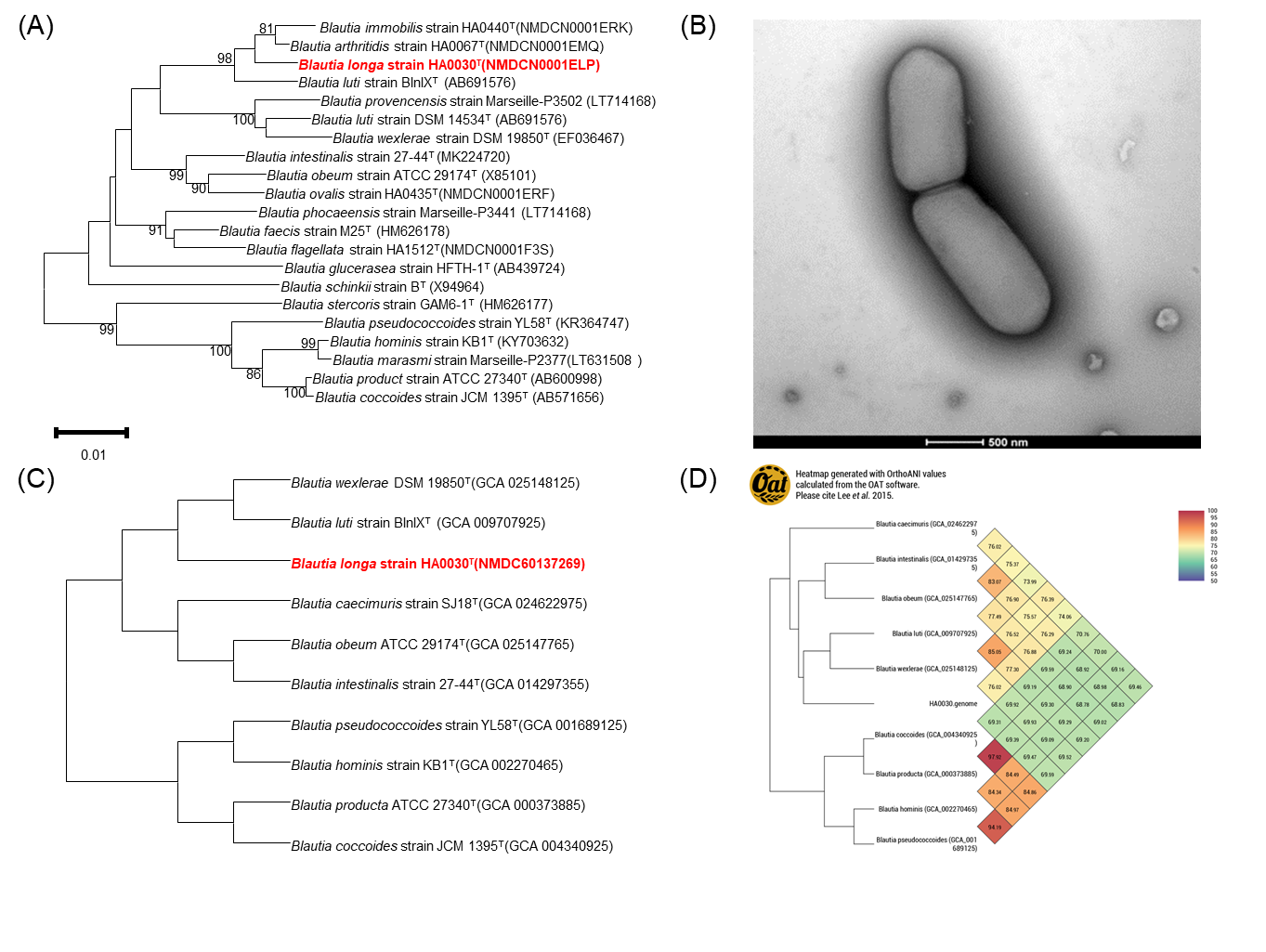


Figure ST-23. The Neighbor-joining phylogenetic tree based on 16S rRNA gene sequences (A) and the cell morphology (B) of strain HA0030^T^. GenBank accession numbers are given in parentheses. Percentages of bootstrap support are shown at branch nodes (values > 70 % are shown). Bootstrap value was 1000. Bar: 0.01 substitutions per nucleotide position. The phylogenomic tree based on genome-sequence-alignment (C) and OrthoANI heatmap (D) of strain HA0030^T^ and its neighbor species. Refseq ID of representative genomes are given in parentheses.

**Taxon 24: *Blautia ovalis* sp. nov**.

**Description of *Blautia ovalis* sp. nov.** (o.va’lis. L. fem. adj. *ovalis*, egg-shaped, denoting shape of the type strain of the species.)

The taxon 24, represented by strain HA0435^T^ (16S rRNA sequence accession number in NMDC is NMDCN0001ERF), is phylogenetically closest to *Blautia obeum* ATCC 29174^T^, with 16S rRNA gene identity of 97.64%. Phylogenetic tree shows that strain HA0435^T^ clusters with other members of the genus *Blautia*, (Figure ST-24A), suggesting strain HA0435^T^ is a member of the genus *Blautia*. The genome of strain HA0435^T^ was sequenced and the NMDC (National Microbiology Data Center) accession number is NMDC60137275. Genome-based analysis showed that the ANI value of genomes of strain HA0435^T^ and *B. obeum* ATCC 29174^T^ (GCA_025147765) is 81.76% and the dDDH estimation is 27.4%. According to the phylogenomic tree (Figure ST-24C), the ANIs and dDDH values of genomes between strain HA0435^T^ and phylogenomically neighbored genomes were calculated, and the highest ANI and dDDH values are 81.76% and 27.4%, respectively, between the query genome of *B. obeum* ATCC 29174^T^; and difference in mol% G+C between genomes of strain HA0435^T^ and *B. obeum* ATCC 29174^T^ is 2.02; OrthoANI heatmap (Figure ST-24D) shows the phylogenomic status of the corresponding neighbor species based on OrthoANI values. Based on these results, we concluded that the strain HA0435^T^ represents of new species of the genus *Blautia*, and the name *Blautia ovalis* sp. nov. is proposed.

Cells are oval-shaped with square ends (2.0 - 3.6 μm long × 1.0 μm wide, Figure ST-24B); cells appear in (dividing pairs) without flagella and non-motile. Growth to stable phase occurs after 48 h incubation in mmGAM medium at 37°C, pH = 7.2. The main fermentation product is acetic acid, and small amounts of propionic and butyric acid can also be produced. The G+C content of the type strain HA0435^T^ is around 43.64 mol%. The type strain HA0435^T^ (= CGMCC 1.48208^T^ = KCTC 25713^T^) was isolated from the faeces of rheumatoid arthritis patients.


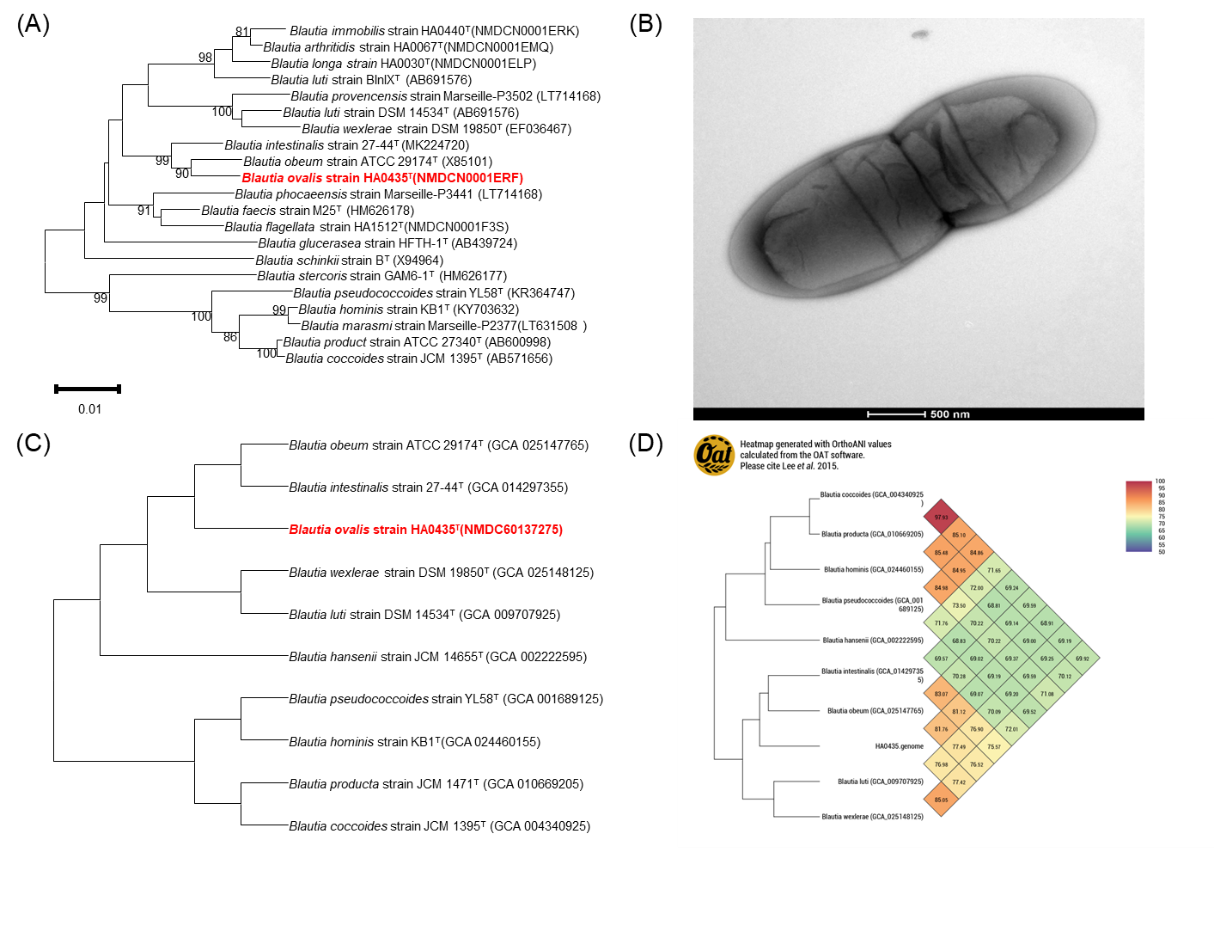


Figure ST-24. The Neighbor-joining phylogenetic tree based on 16S rRNA gene sequences (A) and the cell morphology (B) of strain HA0435^T^. GenBank accession numbers are given in parentheses. Percentages of bootstrap support are shown at branch nodes (values > 70 % are shown). Bootstrap value was 1000. Bar: 0.01 substitutions per nucleotide position. The phylogenomic tree based on genome-sequence-alignment (C) and OrthoANI heatmap (D) of strain HA0435^T^ and its neighbor species. Refseq ID of representative genomes are given in parentheses. **Taxon 25: *Coprococcus immobilis* sp. nov**.

**Description of *Coprococcus immobilis* sp. nov.** (im.mo’bi.lis. L. masc. adj. *immobilis*, non-motile, indicating the non-motility of the type strain)

The taxon 25, represented by strain HA0444^T^ (16S rRNA sequence accession number in NMDC is NMDCN0001ERO), is phylogenetically closest to *Coprococcus catus* VPI-C6-61^T^, with 16S rRNA gene identity of 97.70%. Phylogenetic tree shows that strain HA0444^T^ clusters with other members of the genus *Coprococcus*, (Figure ST-25A), suggesting strain HA0444^T^ is a member of the genus *Coprococcus*. The genome of strain HA0444^T^ was sequenced and the NMDC (National Microbiology Data Center) accession number is NMDC60137280. Genome-based analysis showed that the ANI value of genomes of strain HA0444^T^ and *C. catus* VPI-C6-61^T^ (GCA_019734885) is 89.26% and the dDDH estimation is 40.5%. According to the phylogenomic tree (Figure ST-26C), the ANIs and dDDH values of genomes between strain HA0444^T^ and phylogenomically neighbored genomes were calculated, and the highest ANI and dDDH values are 89.26% and 40.5%, respectively, between the query genome of *C. catus* VPI-C6-61^T^; and difference in mol% G+C between genomes of strain HA0444^T^ and *C. catus* is 0.01; OrthoANI heatmap (Figure ST-25D) shows the phylogenomic status of the corresponding neighbor species based on OrthoANI values. Based on these results, we concluded that the strain HA0444^T^ represents of new species of the genus *Coprococcus*, and the name *Coprococcus immobilis* sp. nov. is proposed.

Cells are oval-shaped (1.0 -1.2 μm long × 0.8 μm wide, Figure ST-25B); cells have no flagella and non-motile. Growth to stable phase occurs after 48 h incubation in mmGAM medium at 37°C, pH = 7.2. The main fermentation product is isovaleric acid. The G+C content of the type strain HA0444^T^ is around 42.94 mol%. The type strain HA0444^T^ (= CGMCC 1.48217^T^ = KCTC 25745^T^) was isolated from the faeces of rheumatoid arthritis patients.


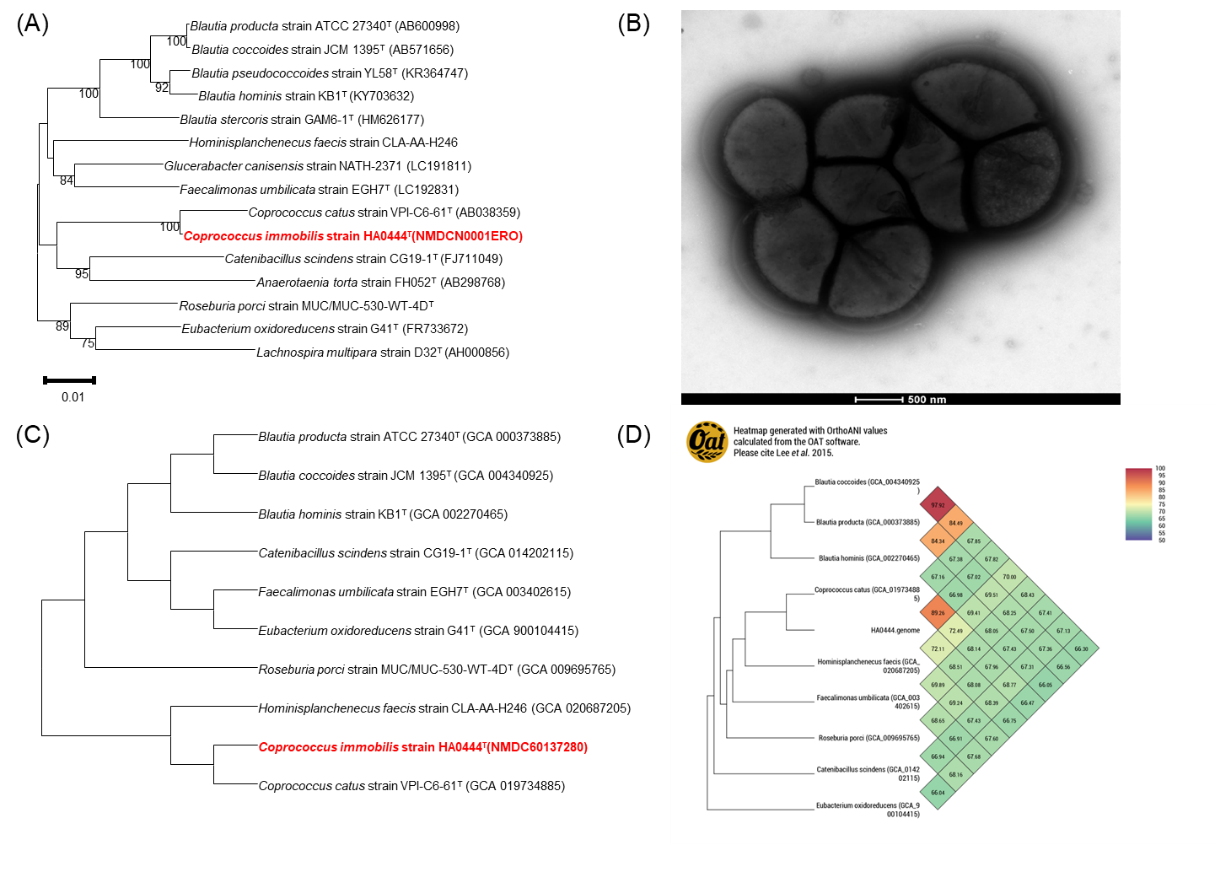


Figure ST-25. The Neighbor-joining phylogenetic tree based on 16S rRNA gene sequences (A) and the cell morphology (B) of strain HA0444^T^. GenBank accession numbers are given in parentheses. Percentages of bootstrap support are shown at branch nodes (values > 70 % are shown). Bootstrap value was 1000. Bar: 0.01 substitutions per nucleotide position. The phylogenomic tree based on genome-sequence-alignment (C) and OrthoANI heatmap (D) of strain HA0444^T^ and its neighbor species. Refseq ID of representative genomes are given in parentheses. **Taxon 26: *Coprococcus mobilis* sp. nov.**

**Description of *Coprococcus* *mobilis* sp. nov.** (mo’bi.lis. L. masc. adj. *mobilis*, mobile.)

The Taxon 26, represented by strain HA0524^T^ (16S rRNA sequence accession number in NMDC is NMDCN0001ESM), is phylogenetically closest to *Coprococcus comes* ATCC 27758^T^*,* with 16S rRNA gene identity of 95.39%. Phylogenetic tree shows that strain HA0524^T^ clusters with other members of the genus *Coprococcus*, (Figure ST-26A), suggesting strain HA0524^T^ is a member of the genus *Coprococcus*. The genome of strain HA0524^T^ was sequenced and the NMDC accession number is NMDC60144116. Genome-based analysis showed that the ANI value of genomes of strain HA0524^T^ and *C. comes* ATCC 27758^T^ (GCA_025149785) is 72.09% and the dDDH estimation is 25.7%. According to the phylogenomic tree (Figure ST-26C), the ANI and dDDH values of genomes between strain HA0524^T^ and phylogenomically neighbored genomes were calculated, and the highest ANI and dDDH values are 72.09% and 25.7%, respectively, between the query genome of *C. comes* ATCC 27758^T^ (GCA_025149785); and difference in mol% G+C between genomes of strain HA0524^T^ and *C. comes* is 2.5%; OrthoANI heatmap (Figure ST-26D) shows the phylogenomic status of the corresponding neighbor species based on OrthoANI values. Based on these results, we concluded that the strain HA0524^T^ represents of new species of the genus *Coprococcus*, and the name *Coprococcus mobilis* sp. nov. is proposed.

Cells are oval-shaped with spiky ends (2.0 -3.2 μm long× 0.8 μm wide, Figure ST-26B); cells appear in dividing pairs with flagella and motile. Growth to stable phase occurs after 48 h incubation in mmGAM medium at 37°C, pH=7.2. The main fermentation products are small amount of acetic, butyric and isovaleric acid. The G+C content of the type strain HA0524^T^ is around 40.00 mol%. The type strain HA0524^T^ (= CGMCC 1.48247^T^) was isolated from the faeces of rheumatoid arthritis patients.


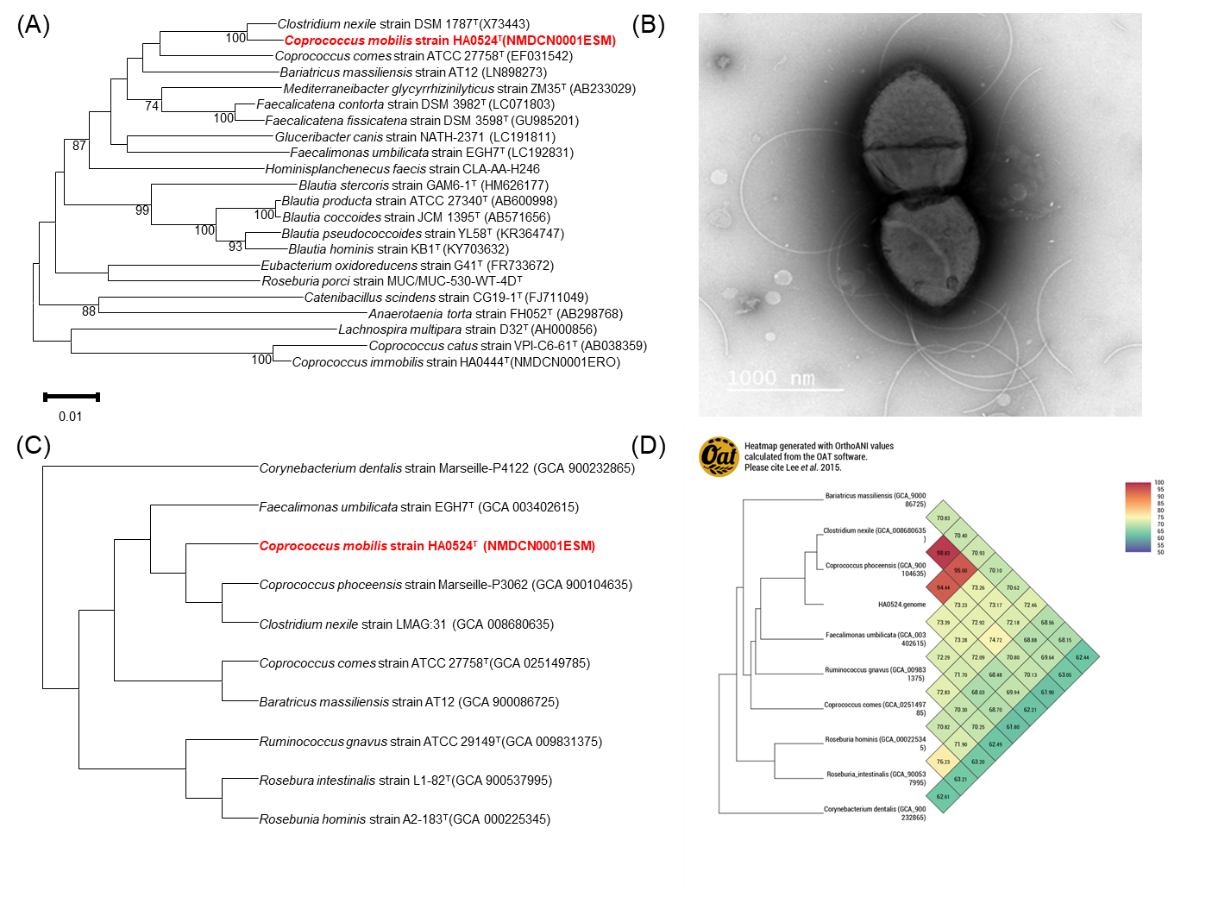


Figure ST-26. The Neighbor-joining phylogenetic tree based on 16S rRNA gene sequences (A) and the cell morphology (B) of strain HA0524^T^. GenBank accession numbers are given in parentheses. Percentages of bootstrap support are shown at branch nodes (values > 70 % are shown). Bootstrap value was 1000. Bar: 0.01 substitutions per nucleotide position. The phylogenomic tree based on genome-sequence-alignment (C) and OrthoANI heatmap (D) of strain HA0524^T^ and its neighbor species. Refseq ID of representative genomes are given in parentheses.

**Taxon 27: *Eisenbergiella longa* sp. nov**.

**Description of *Eisenbergiella longa* sp. nov.** (lon’ga. L. fem. adj. *longa*, long in shape, referring to the cell shape of the type strain.)

The taxon 27, represented by strain HA0447^T^ (16S rRNA sequence accession number in NMDC is NMDCN0001ERR), is phylogenetically closest to *Eisenbergiella tayi* B086562^T^, with 16S rRNA gene identity of 97.52%. Phylogenetic tree shows that strain HA0447^T^ clusters with other members of the genus *Eisenbergiella*, (Figure ST-27A), suggesting strain HA0447^T^ is a member of the genus *Eisenbergiella*. The genome of strain HA0447^T^ was sequenced and the NMDC (National Microbiology Data Center) accession number is NMDC60137283. Genome-based analysis showed that the ANI value of genomes of strain HA0447^T^ and *E. tayi* B086562^T^ (GCA_001881565) is 82.31% and the dDDH estimation is 26.1%. According to the phylogenomic tree (Figure ST-27C), the ANIs and dDDH values of genomes between strain HA0447^T^ and phylogenomically neighbored genomes were calculated, and the highest ANI and dDDH values are 82.31% and 26.1%, respectively, between the query genome of *E. tayi* B086562^T^; and difference in mol% G+C between genomes of strain HA0447^T^ and *E. tayi* B086562^T^ is 1.45; OrthoANI heatmap (Figure ST-27D) shows the phylogenomic status of the corresponding neighbor species based on OrthoANI values. Based on these results, we concluded that the strain HA0447^T^ represents of new species of the genus *Eisenbergiella*, and the name *Eisenbergiella longa* sp. nov. is proposed.

Cells are long rod-shaped (8.0 - 9.2 μm long × 0.6 μm wide, Figure ST-27B); cells appear singly without flagella and non-motile. Growth to stable phase occurs after 48 h incubation in mmGAM medium at 37°C, pH = 7.2. The main fermentation product is acetic and butyric acid, and small amounts of propionic and valeric acid can also be produced. The G+C content of the type strain HA0447^T^ is around 48.23 mol%. The type strain HA0447^T^ (= CGMCC 1.48220^T^ = KCTC 25697^T^) was isolated from the faeces of rheumatoid arthritis patients.


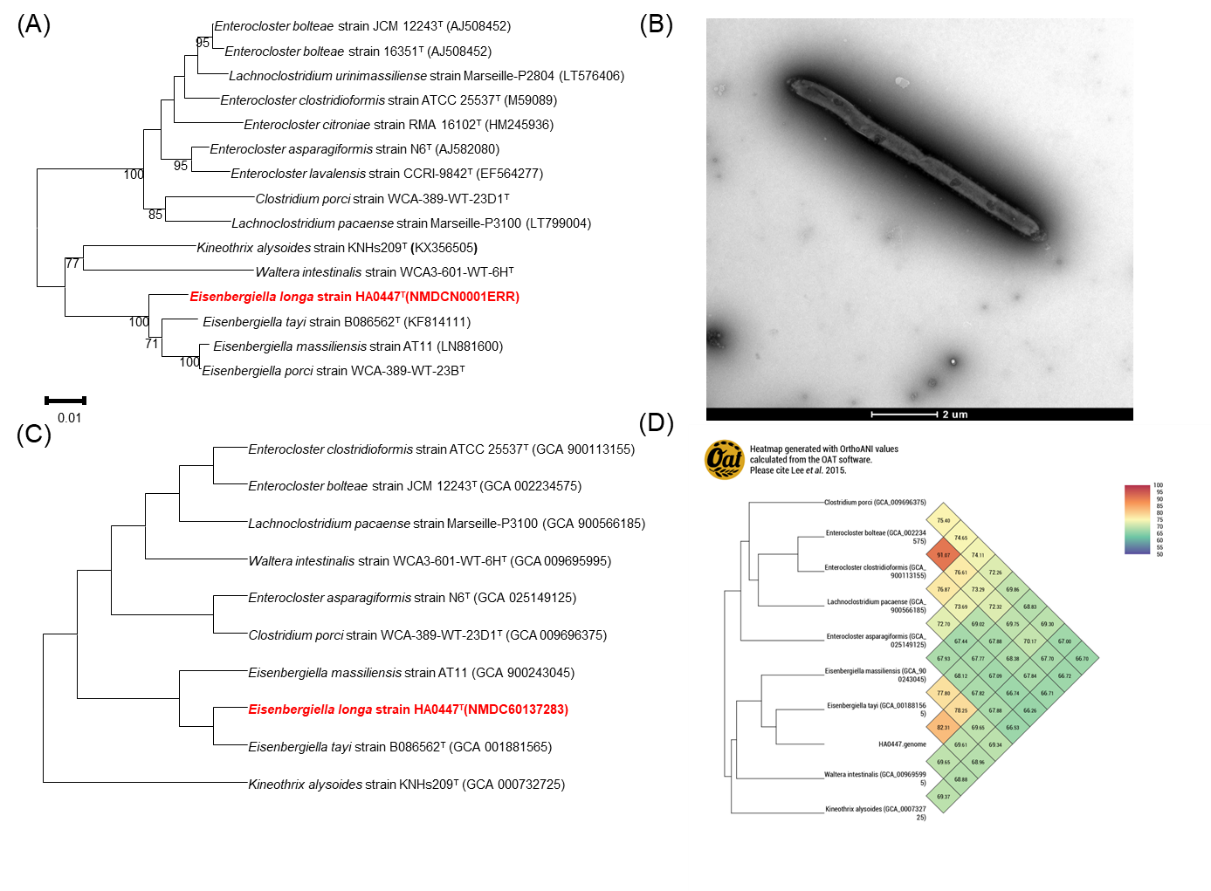


Figure ST-27 The Neighbor-joining phylogenetic tree based on 16S rRNA gene sequences (A) and the cell morphology (B) of strain HA0447^T^. GenBank accession numbers are given in parentheses. Percentages of bootstrap support are shown at branch nodes (values > 70% are shown). Bootstrap value was 1000. Bar: 0.01 substitutions per nucleotide position. The phylogenomic tree based on genome-sequence-alignment (C) and OrthoANI heatmap (D) of strain HA0447^T^ and its neighbor species. Refseq ID of representative genomes are given in parentheses. **Taxon 28: *Jutongia vibrioforma* sp. nov**.

**Description of *Jutongia vibrioforma* sp. nov.** (vi.bri.o.for’ma. L. fem. adj. suff. -*forma*, of the shape of; N.L. fem. adj. *vibrioforma*, shaped like a curved cell, like a vibrio, indicating the shape of the type strain.)

The taxon 28, represented by strain HA0063^T^ (16S rRNA sequence accession number in NMDC is NMDCN0001EMM), is phylogenetically closest to *Jutongia* *huaianensis* NSJ-37^T^, with 16S rRNA gene identity of 95.24%. Phylogenetic tree shows that strain HA0063^T^ is closest to the genus *Jutongia*, (Figure ST-28A), and according to the 16S rRNA gene identity, we consider that strain HA0063^T^ is a member of the genus *Jutongia*. The genome of strain HA0063^T^ was sequenced and the NMDC accession number is NMDC60137270. Genome-based analysis showed that the ANI value of genomes of strain HA0063^T^ and *J. huaianensis* NSJ-37^T^ (GCA_014384985) is 72.38% and the dDDH estimation is 28.8%. According to the phylogenomic tree (Figure ST-28C), the ANIs and dDDH values of genomes between strain HA0063^T^ and phylogenomically neighbored genomes were calculated, and the highest ANI and dDDH values are 72.39% and 28.4%, respectively, between the query genome of *Jutongia hominis* BX3 (GCA_014384965); and difference in mol% G+C between genomes of strain HA0063^T^ and *J. hominis* BX3 is 3.31; OrthoANI heatmap shows the phylogenomic status of the corresponding neighbor species based on OrthoANI values (Figure ST-28D). Based on these results, we concluded that the strain HA0063^T^ represents of new species of the genus *Jutongia*, and the name *Jutongia vibrioforma* sp. nov. is proposed.

Cells are comma-shaped with rounded ends (1.0 - 2.8 μm long × 0.5 μm wide, Figure ST-28B); cells appear singly without flagella and non-motile. Growth to stable phase occurs after 48 h incubation in mmGAM medium at 37°C, pH = 7.2. The main fermentation product is butyric acid. The G+C content of the type strain HA0063^T^ is around 41.89 mol%. The type strain HA0063^T^ (= CGMCC 1.48055^T^) was isolated from the faeces of rheumatoid arthritis patients.


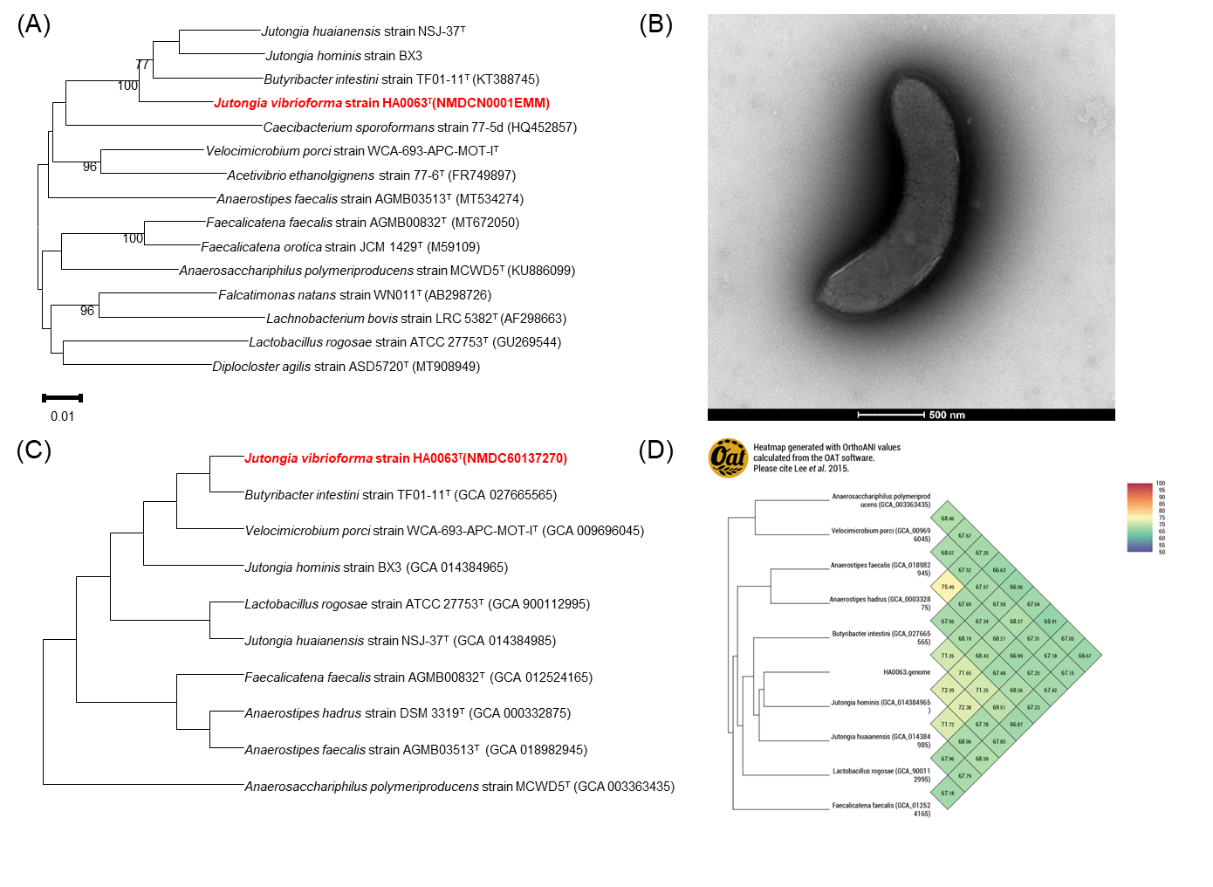


Figure ST-28. The Neighbor-joining phylogenetic tree based on 16S rRNA gene sequences (A) and the cell morphology (B) of strain HA0063^T^. GenBank accession numbers are given in parentheses. Percentages of bootstrap support are shown at branch nodes (values > 70 % are shown). Bootstrap value was 1000. Bar: 0.01 substitutions per nucleotide position. The phylogenomic tree based on genome-sequence-alignment (C) and OrthoANI heatmap (D) of strain HA0063^T^ and its neighbor species. Refseq ID of representative genomes are given in parentheses. **Taxon 29:** ***Lachnospira flagellata* sp. nov**.

**Description of *Lachnospira flagellata* sp. nov.** (fla.gel.la’ta. L. fem. n. *flagellum*, a whip; L. fem. adj. suff. -*ata*, suffix denoting provided with; L. fem. part. adj. *flagellata*, flagellated, denoting the type strain has flagellum.)

The taxon 29, represented by strain HA1242^T^ (16S rRNA sequence accession number in NMDC is NMDCN0001F27), is phylogenetically closest to *Lachnospira pectinoschiza* 150-1^T^, with 16S rRNA gene identity of 93.61%. Phylogenetic tree shows that strain HA1242^T^ clusters with other members of the genus *Lachnospira,* (Figure ST-29A), suggesting strain HA1242^T^ is a member of the genus *Lachnospira.* The genome of strain HA1242^T^ was sequenced and the NMDC (National Microbiology Data Center) accession number is NMDC60137295. Genome-based analysis showed that the ANI value of genomes of strain HA1242^T^ and *L. pectinoschiza* M83 (GCA_900103815) is 66.41% and the dDDH estimation is 21.70%. According to the phylogenomic tree (Figure ST-29C), the ANIs and dDDH values of genomes between strain HA1242^T^ and phylogenomically neighbored genomes were calculated, and the highest ANI and dDDH values are 75.1% and 25.7% , respectively, between the query genome of [*Eubacterium*] *eligens* ATCC 27750^T^ (GCA_000146185); and difference in mol% G+C between genomes of strain HA1242^T^ and *E. eligens* ATCC 27750^T^ is 1.5; OrthoANI heatmap (Figure ST-29D) shows the phylogenomic status of the corresponding neighbor species based on OrthoANI values. Based on these results, we concluded that the strain HA1242^T^ represents of new species of the genus *Lactobacillus*, and the name *Lachnospira flagellata* sp. nov. is proposed.

Cells are rod-shaped with rounded ends (2.0 - 5.4 μm long × 0.8 μm wide, Figure ST-29B); cells have flagella and motile. Growth to stable phase occurs after 24 h incubation in mmGAM medium at 37°C, pH = 7.2. No significant fermentation products detected. The G+C content of the type strain HA1242^T^ is around 36.07 mol%. The type strain HA1242^T^ (= CGMCC 1.48424^T^ = KCTC 25682^T^) was isolated from the faeces of rheumatoid arthritis patients.


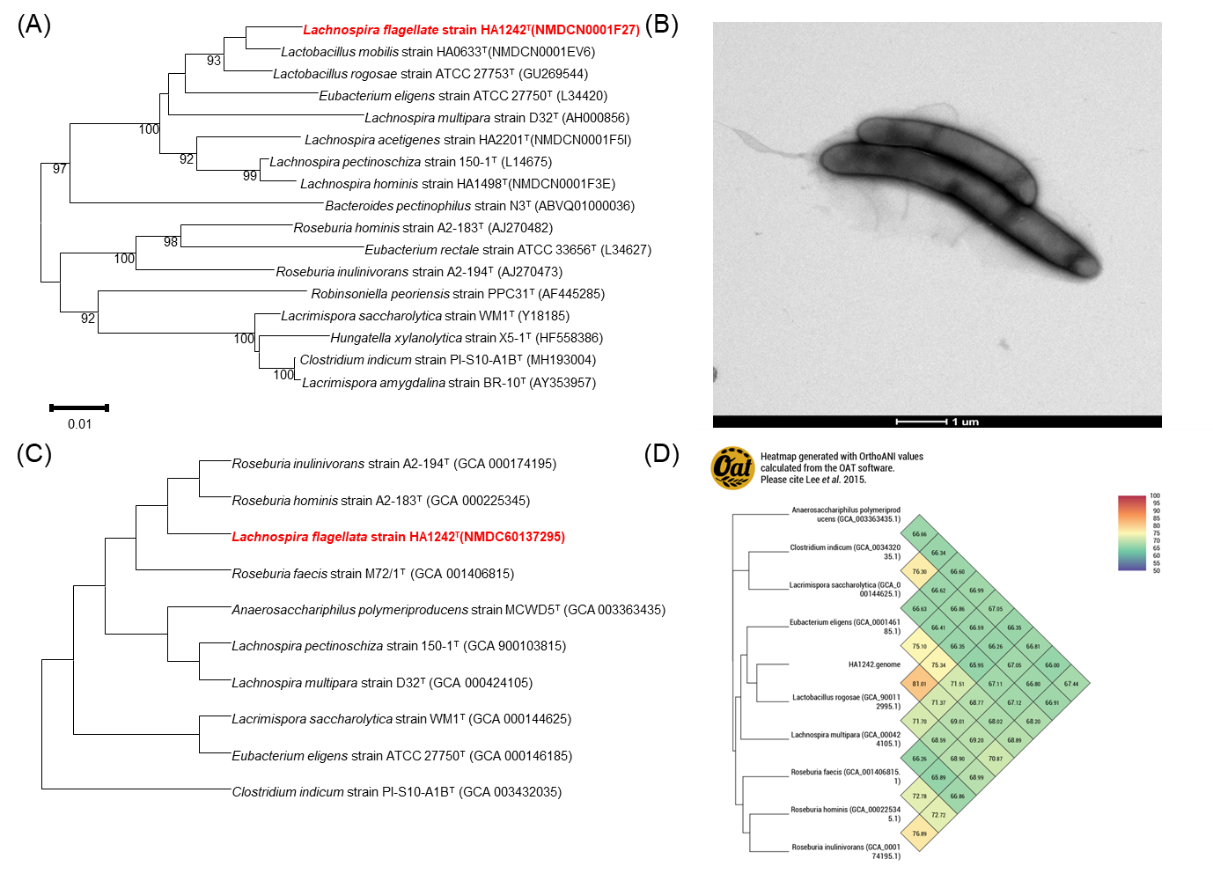


Figure ST-29. The Neighbor-joining phylogenetic tree based on 16S rRNA gene sequences (A) and the cell morphology (B) of strain HA1242^T^. GenBank accession numbers are given in parentheses. Percentages of bootstrap support are shown at branch nodes (values > 70 % are shown). Bootstrap value was 1000. Bar: 0.01 substitutions per nucleotide position. The phylogenomic tree based on genome-sequence-alignment (C) and OrthoANI heatmap (D) of strain HA1242^T^ and its neighbor species. Refseq ID of representative genomes are given in parentheses.

**Taxon 30:** ***Lachnospira mobilis* sp. nov**.

**Description of *Lachnospira mobilis* sp. nov.** (mo’bi.lis. L. fem. adj. *mobilis*, mobile, denoting the type strain is motile.)

The taxon 30, represented by strain HA0633^T^ (16S rRNA sequence accession number in NMDC is NMDCN0001EV6), is phylogenetically closest to *Lachnospira pectinoschiza* 150-1^T^, with 16S rRNA gene identity of 94.24%. Phylogenetic tree shows that strain HA0633^T^ clusters with other members of the genus *Lachnospira,* (Figure ST-30A), suggesting strain HA0633^T^ is a member of the genus *Lachnospira.* The genome of strain HA0633^T^ was sequenced and the NMDC (National Microbiology Data Center) accession number is NMDC60137290. Genome-based analysis showed that the ANI value of genomes of strain HA0633^T^ and *L. pectinoschiza* M83 (GCA_900103815) is 66.39% and the dDDH estimation is 22.8%. According to the phylogenomic tree (Figure ST-30), the ANIs and dDDH values of genomes between strain HA0633^T^ and phylogenomically neighbored genomes were calculated, and the highest ANI and dDDH values are 74.98% and 25.50%, respectively, between the query genome of [*Eubacterium*] *eligens* ATCC 27750^T^ (GCA_000146185); and difference in mol% G+C between genomes of strain HA0633^T^ and *E. eligens* ATCC 27750^T^ is 0.84; OrthoANI heatmap (Figure ST-30D) shows the phylogenomic status of the corresponding neighbor species based on OrthoANI values. Based on these results, we concluded that the strain HA0633^T^ represents of new species of the genus *Lactobacillus*, and the name *Lachnospira mobilis* sp. nov. is proposed.

Cells are rod-shaped with square ends (1.4 - 3.8 μm long × 0.5 μm wide, Figure ST-30B); cells appear singly with flagella and motile. Growth to stable phase occurs after 24 h incubation in mmGAM medium at 37°C, pH = 7.2. No significant fermentation products detected. The G+C content of the type strain HA0633^T^ is around 36.74 mol%. The type strain HA0633^T^ (= CGMCC 1.48327^T^ = KCTC 25680^T^) was isolated from the faeces of rheumatoid arthritis patients.


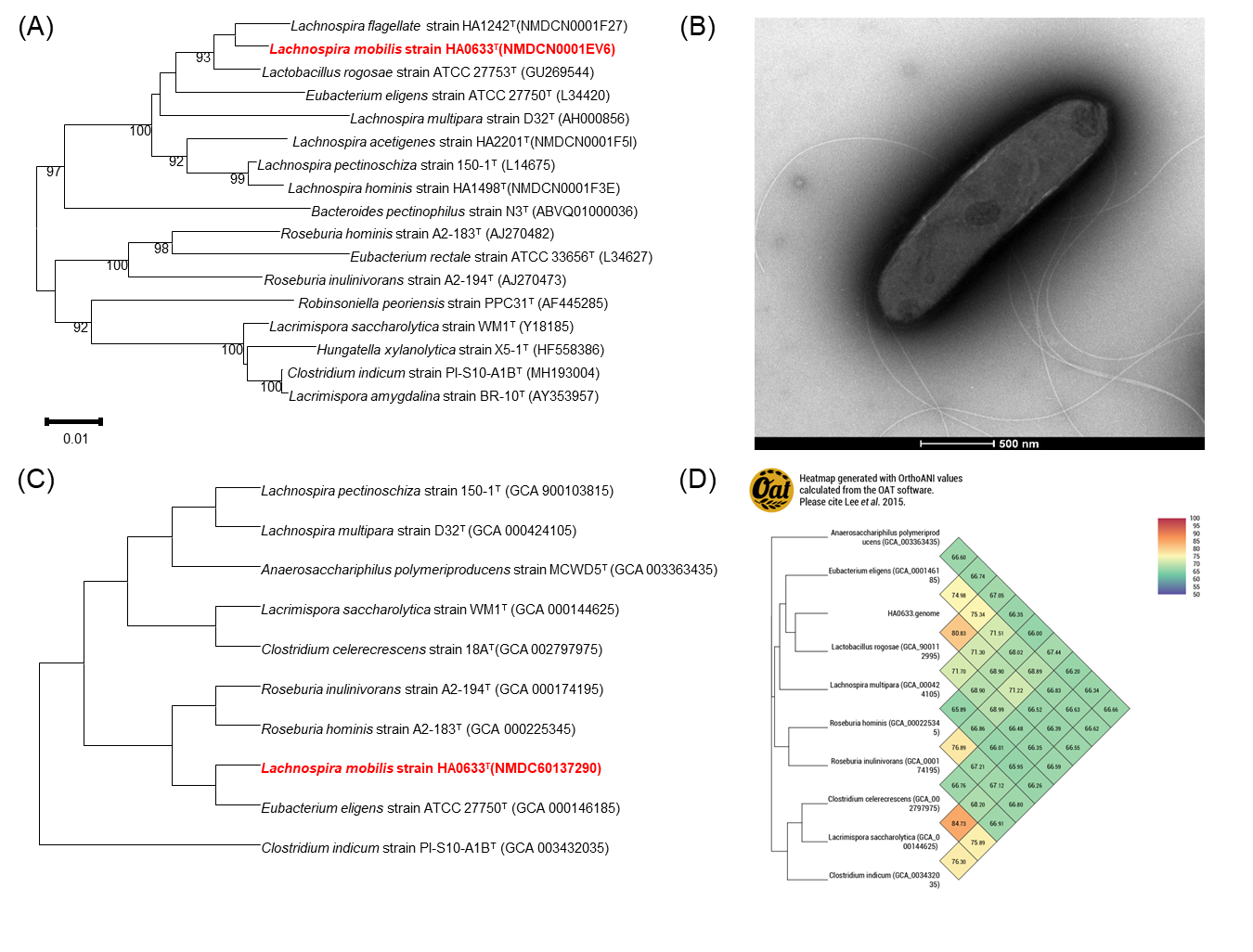


Figure ST-30. The Neighbor-joining phylogenetic tree based on 16S rRNA gene sequences (A) and the cell morphology (B) of strain HA0633^T^. GenBank accession numbers are given in parentheses. Percentages of bootstrap support are shown at branch nodes (values > 70 % are shown). Bootstrap value was 1000. Bar: 0.01 substitutions per nucleotide position. The phylogenomic tree based on genome-sequence-alignment (C) and OrthoANI heatmap (D) of strain HA0633^T^ and its neighbor species. Refseq ID of representative genomes are given in parentheses. **Taxon 31: *Lachnospira* *hominis* sp. nov.**

**Description of *Lachnospira* *hominis* sp. nov.** (ho’mi.nis. L. gen. fem. n. *hominis*, of a human being, referring to the human gut habitat.)

The taxon 31, represented by strain HA1498^T^ (16S rRNA sequence accession number in NMDC is NMDCN0001F3E), is phylogenetically closest to *Lachnospira pectinoschiza* 150-1^T^*,* with 16S rRNA gene identity of 96.84%. Phylogenetic tree shows that strain HA1498^T^ clusters with other members of the genus *Lachnospira*, (Figure ST-31A), suggesting strain HA1498^T^ is a member of the genus *Lachnospira*. The genome of strain HA1498^T^ was sequenced and the NMDC accession number is NMDC60144117. Genome-based analysis showed that the ANI value of genomes of strain HA1498^T^ and *Lachnospira pectinoschiza* M83 (GCA_900103815) is 69.68% and the dDDH estimation is 20.4%. According to the phylogenomic tree (Figure ST-31C), the ANI and dDDH values of genomes between strain HA1498^T^ and phylogenomically neighbored genomes were calculated, and the highest ANI and dDDH values are 71.78% and 26.4% , respectively, between the query genome of *Eubacterium eligens* 27750^T^ (GCA_000146185); and difference in mol% G+C between genomes of strain HA1498^T^ and *E. eligens* 27750^T^ is 3.95; OrthoANI heatmap (Figure ST-31D) shows the phylogenomic status of the corresponding neighbor species based on OrthoANI values. Based on these results, we concluded that the strain HA1498^T^ represents of new species of the genus *Lachnospira*, and the name *Lachnospira* *hominis* sp. nov. is proposed.

Cells are rod-shaped with rounded ends (3.2-5.0 μm long ×0.5 μm wide, Figure ST-31B); cells appear singly with flagella and motile. Growth to stable phase occurs after 48 h incubation in mmGAM medium at 37°C, pH=7. Growth to stable phase occurs after 24 h incubation in mmGAM medium at 37°C, pH=7.2. The main fermentation products are small amount of acetic, butyric, isovaleric and valeric acid. The G+C content of the type strain HA1498^T^ is around 41.53 mol%. The type strain HA1498^T^ (= CGMCC 1.48463^T^ = KCTC 25786^T^) was isolated from the faeces of rheumatoid arthritis patients.


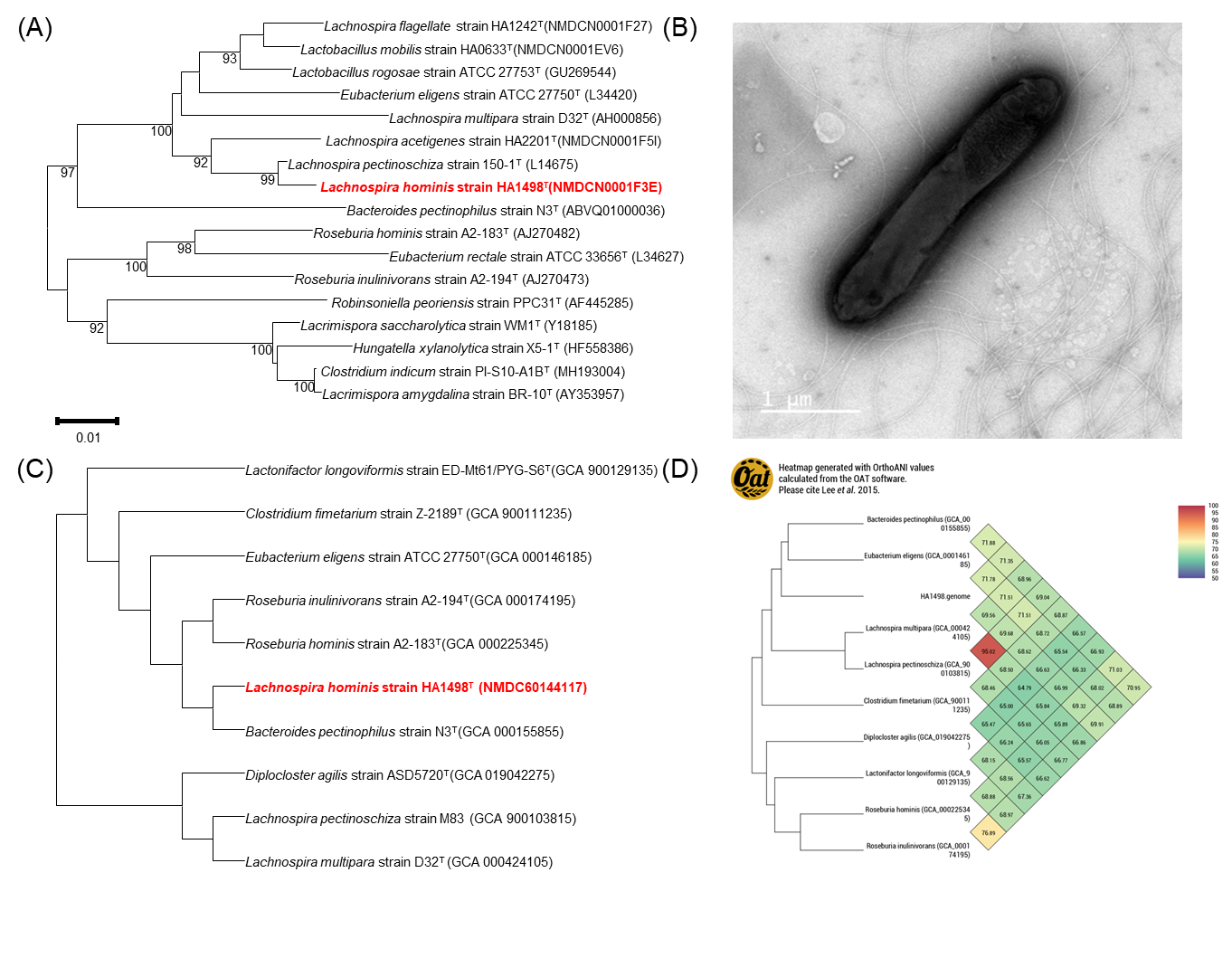


Figure ST-31. The Neighbor-joining phylogenetic tree based on 16S rRNA gene sequences (A) and the cell morphology (B) of strain HA1498^T^. GenBank accession numbers are given in parentheses. Percentages of bootstrap support are shown at branch nodes (values > 70 % are shown). Bootstrap value was 1000. Bar: 0.01 substitutions per nucleotide position. The phylogenomic tree based on genome-sequence-alignment (C) and OrthoANI heatmap (D) of strain HA1498^T^ and its neighbor species. Refseq ID of representative genomes are given in parentheses.

**Taxon 32: *Lachnospira* *acetigenes* sp. nov.**

**Description of *Lachnospira* *acetigenes* sp. nov.** (a.ce.ti’ge.nes. L. fem. n. *acetum*, vinegar; Gr. ind. v. *gennaô*, to produce; N.L. part. adj. *acetigenes*, acetate-producing.)

The Taxon 32, represented by strain HA2201^T^ (16S rRNA sequence accession number in NMDC is NMDCN0001F5I), is phylogenetically closest to *Lachnospira pectinoschiza* 150-1^T^*,* with 16S rRNA gene identity of 94.52%. Phylogenetic tree shows that strain HA2201^T^ clusters with other members of the genus *Lachnospira*, (Figure ST-32A), suggesting strain HA2201^T^ is a member of the genus *Lachnospira*. The genome of strain HA2201^T^ was sequenced and the NMDC accession number is NMDC60144118. Genome-based analysis showed that the ANI value of genomes of strain HA2201^T^ and *Lachnospira pectinoschiza* M83 (GCA_900103815) is 70.19% and the dDDH estimation is 20.3%. According to the phylogenomic tree (Figure ST-32C), the ANI and dDDH values of genomes between strain HA2201^T^ and phylogenomically neighbored genomes were calculated, and the highest ANI and dDDH values are 73.93% and 24.4%, respectively, between the query genome of [*Eubacterium*] *eligens* ATCC 27750^T^ (GCA_000146185); and difference in mol% G+C between genomes of strain HA2201^T^ and *E. eligens* ATCC 27750^T^ is 0.72; OrthoANI heatmap (Figure ST-32D) shows the phylogenomic status of the corresponding neighbor species based on OrthoANI values. Based on these results, we concluded that the strain HA2201^T^ represents of new species of the genus *Lachnospira*, and the name *Lachnospira* *acetigenes* sp. nov. is proposed.

Cells are rod-shaped with rounded ends (2.2 – 4.9 μm long × 0.5 μm wide, Figure ST-32B); cells appear singly with flagella and motile. Growth to stable phase occurs after 24 h incubation in mGAM medium at 37°C, pH=7.2. The main fermentation product is small amount of acetic acid. The G+C content of the type strain HA2201^T^ is around 36.85 mol%. The type strain HA2201^T^ (= CGMCC 1.48531^T^) was isolated from the faeces of rheumatoid arthritis patients.


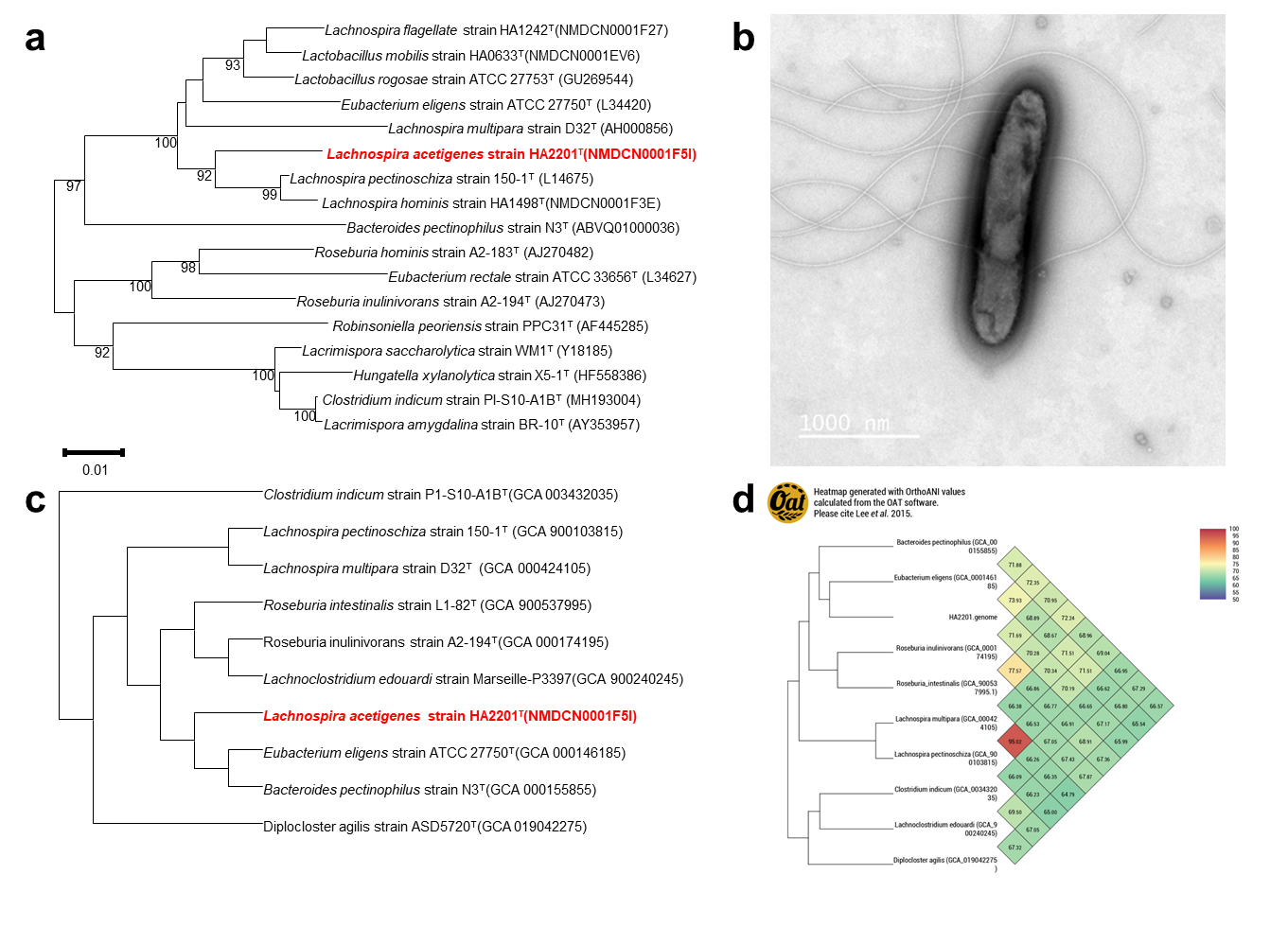


Figure ST-32. The Neighbor-joining phylogenetic tree based on 16S rRNA gene sequences (A) and the cell morphology (B) of strain HA2201^T^. GenBank accession numbers are given in parentheses. Percentages of bootstrap support are shown at branch nodes (values > 70 % are shown). Bootstrap value was 1000. Bar: 0.01 substitutions per nucleotide position. The phylogenomic tree based on genome-sequence-alignment (C) and OrthoANI heatmap (D) of strain HA2201^T^ and its neighbor species. Refseq ID of representative genomes are given in parentheses.

**Taxon 33: *Lactonifactor hominis* sp. nov**.

**Description of *Lactonifactor hominis* sp. nov.** (ho’mi.nis. L. gen. masc. n. *hominis*, of a human being, indicating that the type strain was isolated from a human.)

The taxon 33, represented by strain HA0443^T^ (16S rRNA sequence accession number in NMDC is NMDCN0001ERN), is phylogenetically closest to *Lactonifactor longoviformis* ED-Mt61/PYG-S6^T^, with 16S rRNA gene identity of 97.62%. Phylogenetic tree shows that strain HA0443^T^ clusters with other members of the genus *Lactonifactor*, (Figure ST-33A), suggesting strain HA0443^T^ is a member of the genus *Lactonifactor*. The genome of strain HA0443^T^ was sequenced and the NMDC (National Microbiology Data Center) accession number is NMDC60137279. Genome-based analysis showed that the ANI value of genomes of strain HA0443^T^ and *L. longoviformis* ED-Mt61/PYG-S6^T^ (GCA_900129135) is 79.29 % and the dDDH estimation is 23.00%. According to the phylogenomic tree (Figure ST-33C), the ANIs and dDDH values of genomes between strain HA0443^T^ and phylogenomically neighbored genomes were calculated, and the highest ANI and dDDH values are 79.29% and 23.00%, respectively, between the query genome of *L. longoviformis* ED-Mt61/PYG-S6^T^; and difference in mol% G+C between genomes of strain HA0443^T^ and *L. longoviformis* ED-Mt61/PYG-S6^T^ is 0.79; OrthoANI heatmap (Figure ST-33D) shows the phylogenomic status of the corresponding neighbor species based on OrthoANI values. Based on these results, we concluded that the strain HA0443^T^ represents of new species of the genus *Lactonifactor*, and the name *Lactonifactor hominis* sp. nov. is proposed.

Cells are oval-shaped (1.4 - 2.0 μm long × 1.0 μm wide, Figure ST-33B); cells appear singly without flagella and non-motile. Growth to stable phase occurs after 48 h incubation in mmGAM medium at 37°C, pH = 7.2. No significant fermentation products detected. The G+C content of the type strain HA0443^T^ is around 45.6 mol%. The type strain HA0443^T^ (= CGMCC 1.48216^T^ = KCTC 25744^T^) was isolated from the faeces of rheumatoid arthritis patients.


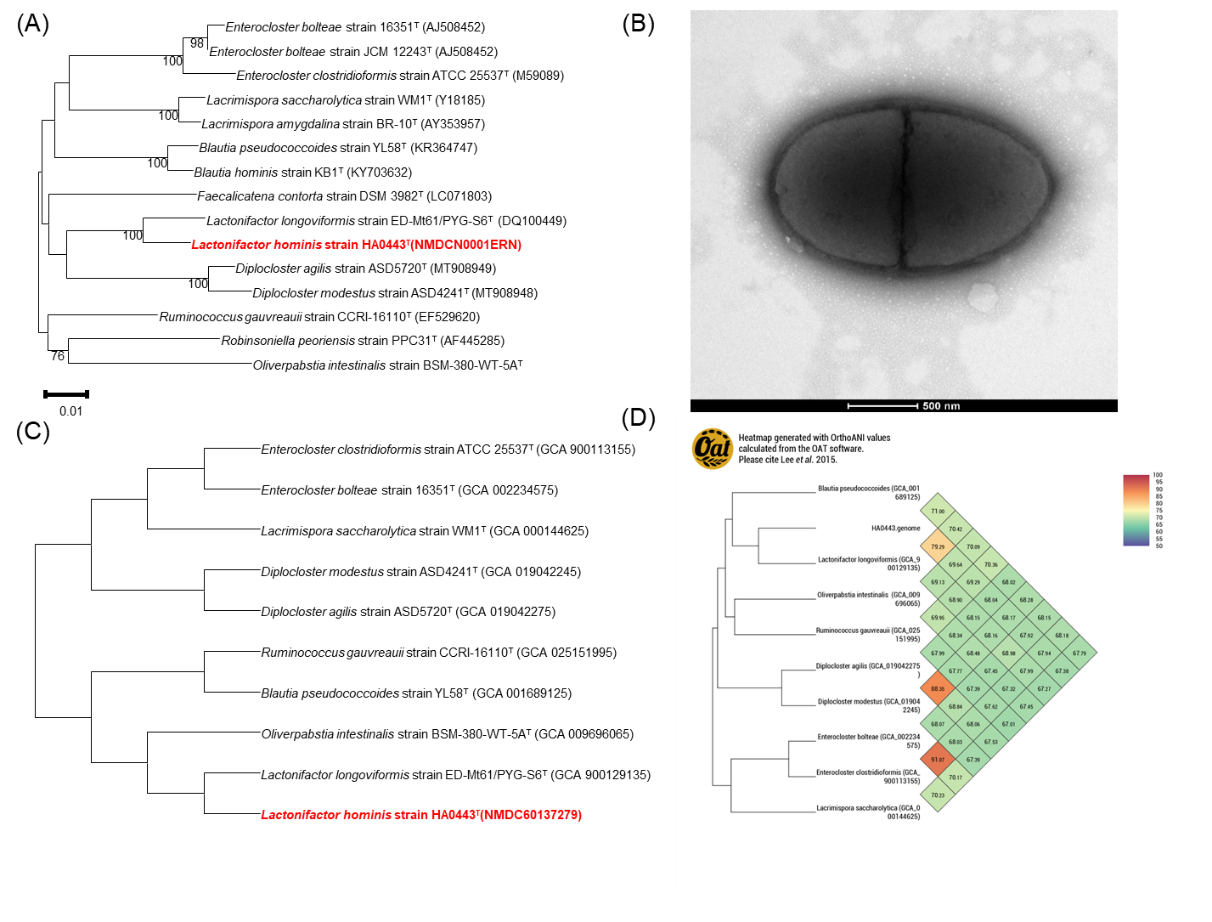


Figure ST-33. The Neighbor-joining phylogenetic tree based on 16S rRNA gene sequences (A) and the cell morphology (B) of strain HA0443^T^. GenBank accession numbers are given in parentheses. Percentages of bootstrap support are shown at branch nodes (values > 70 % are shown). Bootstrap value was 1000. Bar: 0.01 substitutions per nucleotide position. The phylogenomic tree based on genome-sequence-alignment (C) and OrthoANI heatmap (D) of strain HA0443^T^ and its neighbor species. Refseq ID of representative genomes are given in parentheses.

**Taxon** **34: *Mediterraneibacter tenuis* sp. nov.**

**Description of *Mediterraneibacter tenuis* sp. nov. (**te’nu.is. L. masc. adj. *tenuis*, slender, indicating the shape of the type strain.**)**

The taxon 34, represented by strain HA0437^T^ (16S rRNA sequence accession number in NMDC is NMDCN0001ERH), is phylogenetically closest to *Mediterraneibacter gnavus* ATCC 29149^T^ (Figure ST-34A), with 16S rRNA gene identity of 93.35%. The genome of strain HA0437^T^ was sequenced and the NMDC (National Microbiology Data Center) accession number is NMDC60137276. Genome-based analysis on *M. gnavus* ATCC 29149^T^ and type strain HA0437^T^ revealed that the dDDH, ANI and POCP values between *M. gnavus* ATCC 29149^T^ (GCA_009831375) and strain HA0437^T^ are 30.4%, 68.90% and 57.36%, suggesting *M. gnavus* ATCC 29149^T^ and strain HA0437^T^ are from same genera. According to the phylogenomic tree (Figure ST-34C), the ANIs and dDDH values of genomes between strain HA0437^T^ and phylogenomically neighbored genomes were calculated, and the highest ANI and dDDH values are 74.89% and 36.2%, respectively, between the query genome of *Clostridium fessum* SNUG30386^T^ (GCA_003024715); and difference in mol% G+C between genomes of strain HA0437^T^ and *C. fessum* SNUG30386^T^ is 0.37; OrthoANI heatmap (Figure ST-34D) shows the phylogenomic status of the corresponding neighbor species based on OrthoANI values. Based on these results, we concluded that the strain HA0437^T^ represents of new species of the genus *Mediterraneibacter*, and the name *Mediterraneibacter tenuis* sp. nov. is proposed.

Cells are rods with tapered ends (2.0 - 6.0 μm long × 0.5 μm wide, Figure ST-34B); cells appear singly without flagella and non-motile. Growth to stable phase occurs after 48 h incubation in mmGAM medium at 37°C, pH = 7.2. The main fermentation product is butyric acid. The G+C content of the type strain HA0437^T^ is around 48.66 mol%. The type strain HA0437^T^ (=CGMCC 1.48201^T^) was isolated from the faeces of rheumatoid arthritis patients.


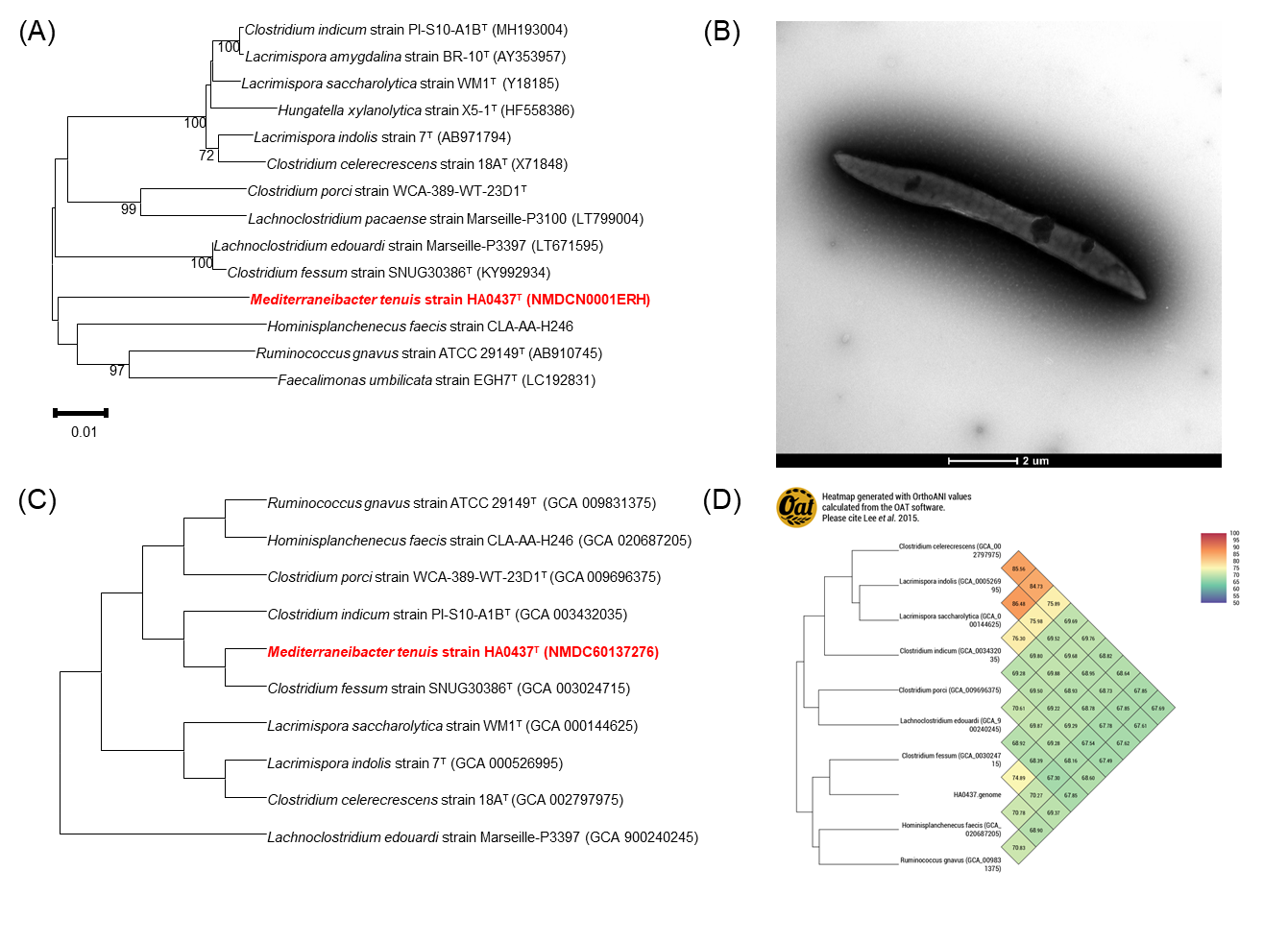


Figure ST-34. The Neighbor-joining phylogenetic tree based on 16S rRNA gene sequences (A) and the cell morphology (B) of strain HA0437^T^. GenBank accession numbers are given in parentheses. Percentages of bootstrap support are shown at branch nodes (values > 70 % are shown). Bootstrap value was 1000. Bar: 0.01 substitutions per nucleotide position. The phylogenomic tree based on genome-sequence-alignment (C) and OrthoANI heatmap (D) of strain HA0437^T^ and its neighbor species. Refseq ID of representative genomes are given in parentheses. **Taxon 35: *Waltera fermentans* sp. nov**.

**Description of *Waltera fermentans* sp. nov.** (fer.men’tans. L. fem. adj. *fermentans*, fermenting, indicating that the type strain is a fermentative bacterium.)

The taxon 35, represented by strain HA1509^T^ (16S rRNA sequence accession number in NMDC is NMDCN0001F3P), is phylogenetically closest to *Waltera intestinalis* WCA3-601-WT-6H^T^, with 16S rRNA gene identity of 95.04%. Phylogenetic tree shows that strain HA1509^T^ clusters with other members of the genus *Waltera,* (Figure ST-35A), suggesting strain HA1509^T^ is a member of the genus *Waltera.* The genome of strain HA1509^T^ was sequenced and the NMDC (National Microbiology Data Center) accession number is NMDC60137300. Genome-based analysis showed that the ANI value of genomes of strain HA1509^T^ and *W. intestinalis* WCA3-601-WT-6H^T^ (GCA_009695995) is 70.85% and the dDDH estimation is 20.90%. According to the phylogenomic tree (Figure ST-35C), the ANIs and dDDH values of genomes between strain HA1509^T^ and phylogenomically neighbored genomes were calculated, and the highest ANI and dDDH values are 71.36% and 48.0%, respectively, between the query genome of *Clostridium fessum* SNUG30386^T^ (GCA_003024715); and difference in mol% G+C between genomes of strain HA1509^T^ and *C. fessum* SNUG30386^T^ is 4.26; OrthoANI heatmap (Figure ST-35D) shows the phylogenomic status of the corresponding neighbor species based on OrthoANI values. Based on these results, we concluded that the strain HA1509^T^ represents of new species of the genus *Waltera*, and the name *Waltera fermentans* sp. nov. is proposed.

Cells are rod-shaped with rounded ends (4.2 - 8.4 μm long × 0.5 - 1.6 μm wide, Figure ST-35B); cells appear singly with flagella and motile. After grows to the end of the logarithmic phase, the middle of the cells expands to produce a spherical shape (about 1.5 μm wide in diameter). Growth to stable phase occurs after 48 h incubation in mmGAM medium at 37°C, pH = 7.2. The main fermentation products are acetic and butyric acid, and small amounts of isovaleric and valeric acid can also be produced. The G+C content of the type strain HA1509^T^ is around 44.03 mol%. The type strain HA1509^T^ (= CGMCC 1.17997^T^ = KCTC 25717^T^) was isolated from the faeces of rheumatoid arthritis patients.


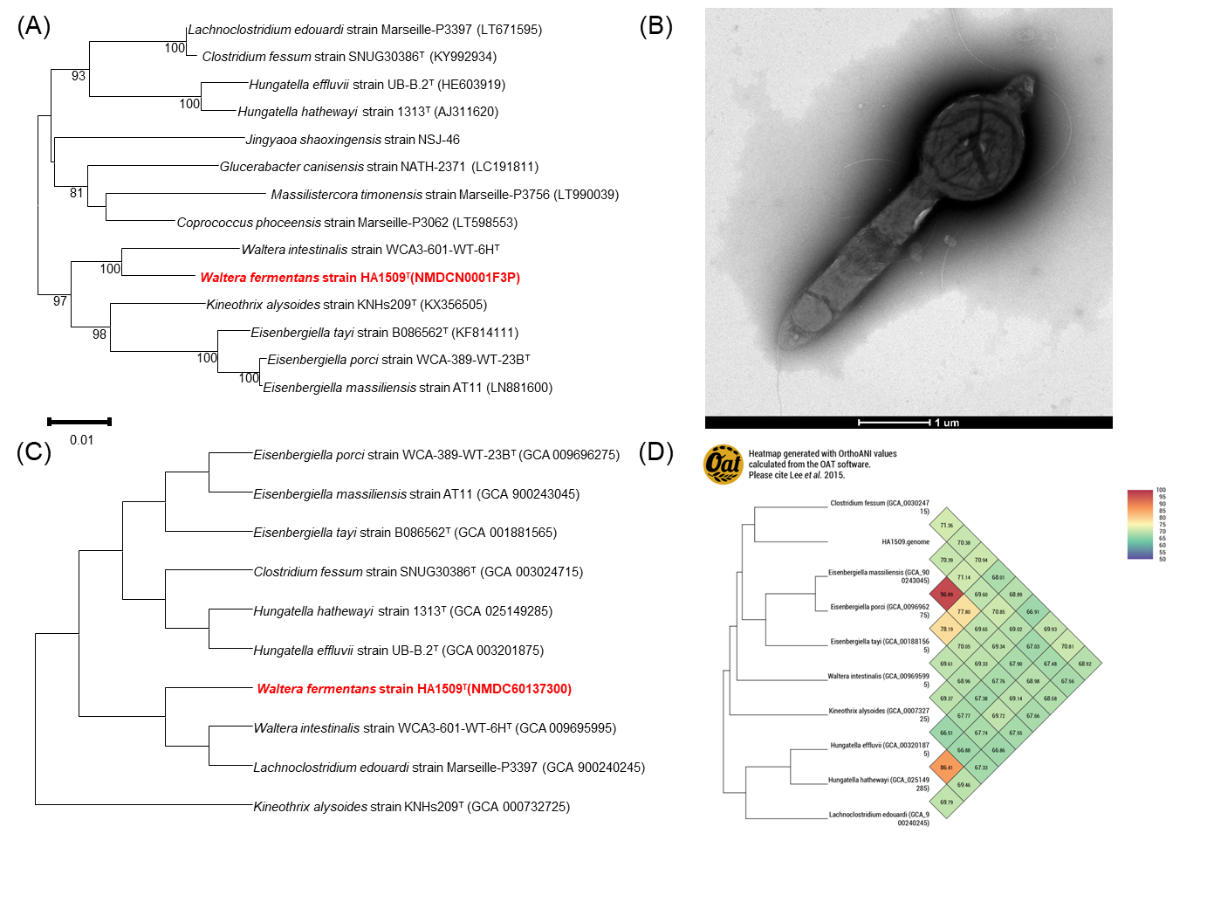


Figure ST-35. The Neighbor-joining phylogenetic tree based on 16S rRNA gene sequences (A) and the cell morphology (B) of strain HA1509^T^. GenBank accession numbers are given in parentheses. Percentages of bootstrap support are shown at branch nodes (values > 70 % are shown). Bootstrap value was 1000. Bar: 0.01 substitutions per nucleotide position. The phylogenomic tree based on genome-sequence-alignment (C) and OrthoANI heatmap (D) of strain HA1509^T^ and its neighbor species. Refseq ID of representative genomes are given in parentheses. **Taxon 36: *Negativibacillus intestinalis*** **sp. nov.**

**Description of *Negativibacillus intestinalis* sp. nov.** (in.tes.ti.na’lis. N.L. masc. adj. *intestinalis*, of the gut, indicating that the type strain was isolated from the gut.)

The taxon 36, represented by strain HA0568^T^ (16S rRNA sequence accession number in NMDC is NMDCN0001EU0), is phylogenetically closest to *Negativibacillus massiliensis* Maraeille-P3213^T^, with 16S rRNA gene identity of 97.60%. Phylogenetic tree shows that strain HA0568^T^ clusters with other members of the genus *Negativibacillus,* (Figure ST-36A), suggesting strain HA0568^T^ is a member of the genus *Negativibacillus.* The genome of strain HA0568^T^ was sequenced and the NMDC (National Microbiology Data Center) accession number is NMDC60137287. Genome-based analysis showed that the ANI value of genomes of strain HA0568^T^ and *N. massiliensis* Maraeille-P3213^T^ (GCA_900148495) is 75.69% and the dDDH estimation is 21.70%. According to the phylogenomic tree (Figure ST-36C), the ANIs and dDDH values of genomes between strain HA0568^T^ and phylogenomically neighbored genomes were calculated, and the highest ANI and dDDH values are 75.69% and 21.70%, respectively; between the query genome of *N. massiliensis* Maraeille-P3213^T^, and difference in mol% G+C between genomes of strain HA0568^T^ and *N. massiliensis* Maraeille-P3213^T^ is 6.31; OrthoANI heatmap (Figure ST-36D) shows the phylogenomic status of the corresponding neighbor species based on OrthoANI values. Based on these results, we concluded that the strain HA0568^T^ represents of new species of the genus *Negativibacillus*, and the name *Negativibacillus intestinalis* sp. nov. is proposed.

Cells are rod-shaped with rounded ends (1.5 - 6.6 μm long × 0.6 μm wide, Figure ST-36B); cells appear in (dividing) pairs without flagella and non-motile. Growth to stable phase occurs after 48 h incubation in mmGAM medium at 37°C, pH = 7.2. No significant fermentation products detected. The G+C content of the type strain HA0568^T^ is around 51.72 mol%. The type strain HA0568^T^ (= CGMCC 1.48289^T^ = KCTC 25700^T^) was isolated from the faeces of rheumatoid arthritis patients.


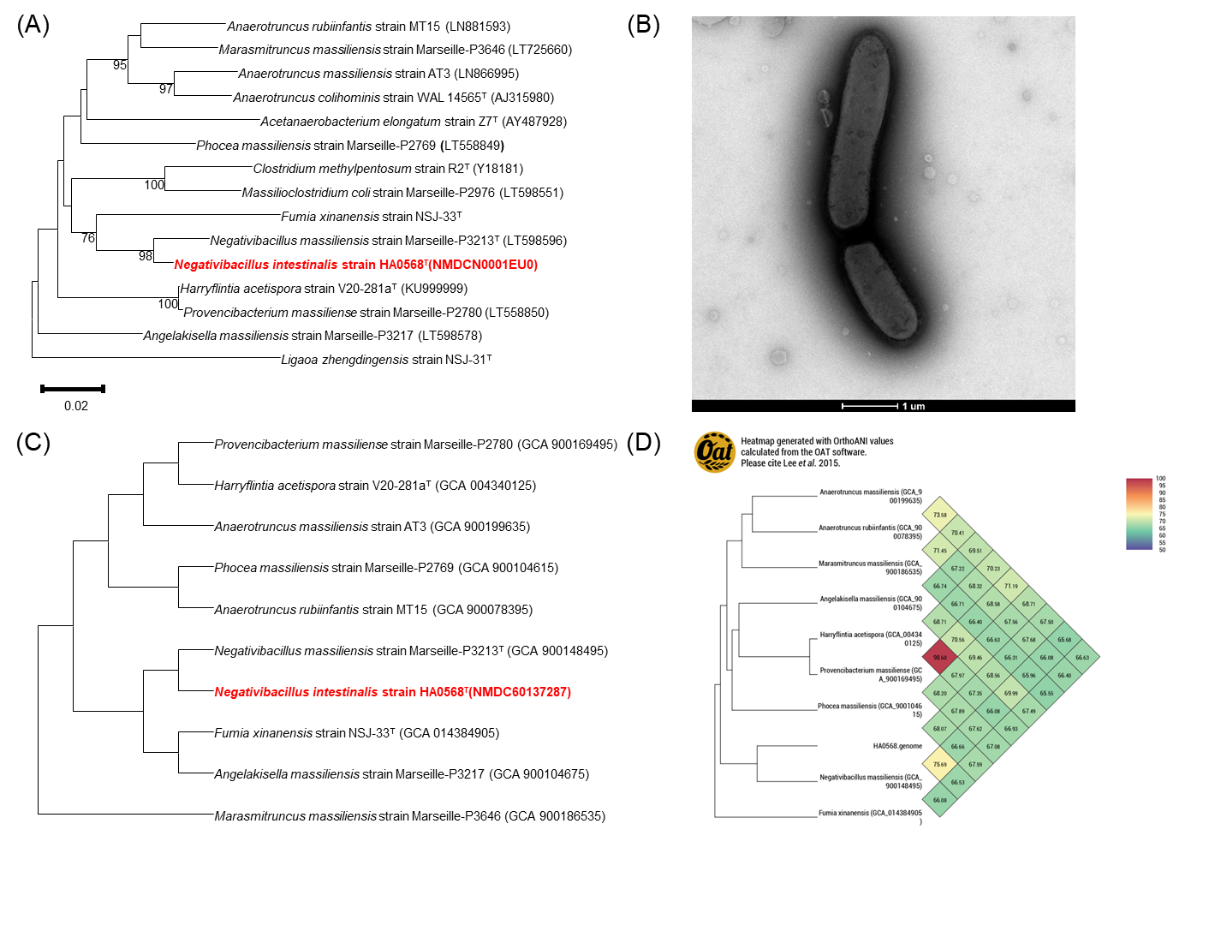


Figure ST-36. The Neighbor-joining phylogenetic tree based on 16S rRNA gene sequences (A) and the cell morphology (B) of strain HA0568^T^. GenBank accession numbers are given in parentheses. Percentages of bootstrap support are shown at branch nodes (values > 70 % are shown) . Bootstrap value was 1000. Bar: 0.02 substitutions per nucleotide position. The phylogenomic tree based on genome-sequence-alignment (C) and OrthoANI heatmap (D) of strain HA0568^T^ and its neighbor species. Refseq ID of representative genomes are given in parentheses.

**Taxon 37: *Pseudoflavonifractor intestinalis* sp. nov**.

**Description of *Pseudoflavonifractor intestinalis* sp. nov. (**in.tes.ti.na’lis. N.L. masc. adj. *intestinalis*, pertaining to the intestine, denoting the type strain was isolated from the intestine.**)**

The taxon 37, represented by strain HA1510^T^ (16S rRNA sequence accession number in NMDC is NMDCN0001F3Q), is phylogenetically closest to *Pseudoflavonifractor gallinarum* Cla-CZ-98^T^, with a 16S rRNA gene identity of 95.53%. Phylogenetic tree shows that strain HA1510^T^ clusters with other members of the genus *Pseudoflavonifractor* (Figure ST-37A), suggesting that strain HA1510^T^ is a member of the genus *Pseudoflavonifractor*. The genome of strain HA1510^T^ was sequenced and the NMDC accession number is NMDC60137301. Genome-based analysis showed that the ANI value of the genomes of strain HA1510^T^ and *P. gallinarum* Cla-CZ-98^T^ (GCA_014982855) was 72.69% and the dDDH estimation was 22.5%. According to the phylogenomic tree (Figure ST-37C), the ANIs and dDDH values of genomes between strain HA1510^T^ and phylogenomically neighbored genomes were calculated, and the highest ANI and dDDH values are 74.05% and 25.6%, respectively, between the query genome of *Intestinimonas massiliensis* GD2^T^ (GCA_001244995); and difference in mol% G+C between genomes of strain HA1510^T^ and *I*. *massiliensis* GD2^T^ is 4.38; OrthoANI heatmap (Figure ST-37D) shows the phylogenomic status of the corresponding neighbor species based on OrthoANI values. Based on these results, we concluded that the strain HA1510^T^ represents of new species of the genus *Pseudoflavonifractor*, and the name *Pseudoflavonifractor intestinalis* sp. nov. is proposed.

Cells are long rod-shaped with spiky ends (6.2 - 11.2 μm long × 0.5 μm wide, Figure ST-37B); cells appear singly without flagella and non-motile. Growth to stable phase occurs after 48 h incubation in mmGAM medium at 37°C, pH = 7.2. The main fermentation product is butyric acid. The G+C content of the type strain HA1510^T^ is around 56.3 mol%. The type strain HA1510^T^ (= CGMCC 1.17998^T^ = KCTC 25754^T^) was isolated from the faeces of rheumatoid arthritis patients.


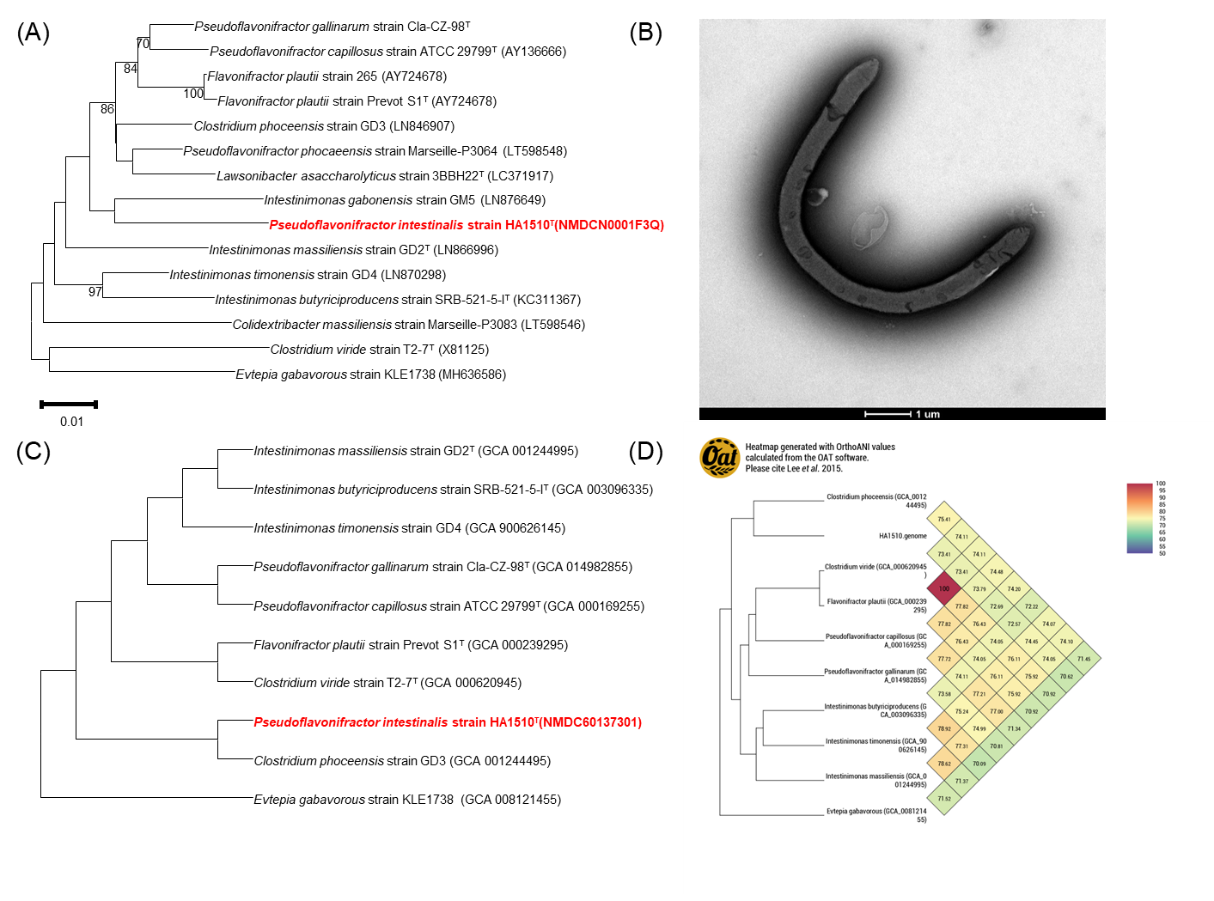


Figure ST-37. The Neighbor-joining phylogenetic tree based on 16S rRNA gene sequences (A) and the cell morphology (B) of strain HA1510^T^. GenBank accession numbers are given in parentheses. Percentages of bootstrap support are shown at branch nodes (values > 70 % are shown). Bootstrap value was 1000. Bar: 0.01 substitutions per nucleotide position. The phylogenomic tree based on genome-sequence-alignment (C) and OrthoANI heatmap (D) of strain HA1510^T^ and its neighbor species. Refseq ID of representative genomes are given in parentheses.

**Taxon 38: *Vescimonas butyriciproducens* sp. nov.**

**Description of *Vescimonas butyriciproducens* sp. nov.** (bu.ty.ri.ci.pro.du’cens. N.L. fem. n. *acidum butyricum*, butyric acid; L. pres. part. producens, producing; N.L. part. adj. *butyriciproducens*, producing butyric acid, denoting the type strain produces butyric acid.)

The taxon 38, represented by strain HA1201^T^ (16S rRNA sequence accession number in NMDC is NMDCN0001F0U), is phylogenetically closest to *Vescimonas fastidiosa* MM35^T^, with 16S rRNA gene identity of 95.32%. Phylogenetic tree shows that strain HA1201^T^ clusters with other members of the genus *Vescimonas,* (Figure ST-38A), suggesting strain HA1201^T^ is a member of the genus *Vescimonas.* The genome of strain HA1201^T^ was sequenced and the NMDC (National Microbiology Data Center) accession number is NMDC60137294. Genome-based analysis showed that the ANI value of genomes of strain HA1201^T^ and *V. fastidiosa* MM35^T^ (GCA_018326305) is 74.41% and the dDDH estimation is 33.30%. According to the phylogenomic tree (Figure ST-38C), the ANIs and dDDH values of genomes between strain HA1201^T^ and phylogenomically neighbored genomes were calculated, and the highest ANI and dDDH values are 75.17% and 33.10%, respectively, between the query genome of *Vescimonas coprocola* MM50^T^ (GCA_018408575); and difference in mol% G+C between genomes of strain HA1201^T^ and *V. coprocola* is 1.76; OrthoANI heatmap (Figure ST-38D) shows the phylogenomic status of the corresponding neighbor species based on OrthoANI values. Based on these results, we concluded that the strain HA1201^T^ represents of new species of the genus *Vescimonas*, and the name *Vescimonas butyriciproducens* sp. nov. is proposed.

Cells are rod-shaped with rounded ends (2.0 - 4.2 μm long × 0.75 μm wide, Figure ST-38B); cells appear singly without flagella and non-motile. Growth to stable phase occurs after 72 h incubation in mmGAM medium at 37°C, pH = 7.2. The main fermentation product is small amount of butyric acid. The G+C content of the type strain HA1201^T^ is around 56.45 mol%. The type strain HA1201^T^ (= CGMCC 1.48383^T^ = KCTC 25704^T^) was isolated from the faeces of rheumatoid arthritis patients.


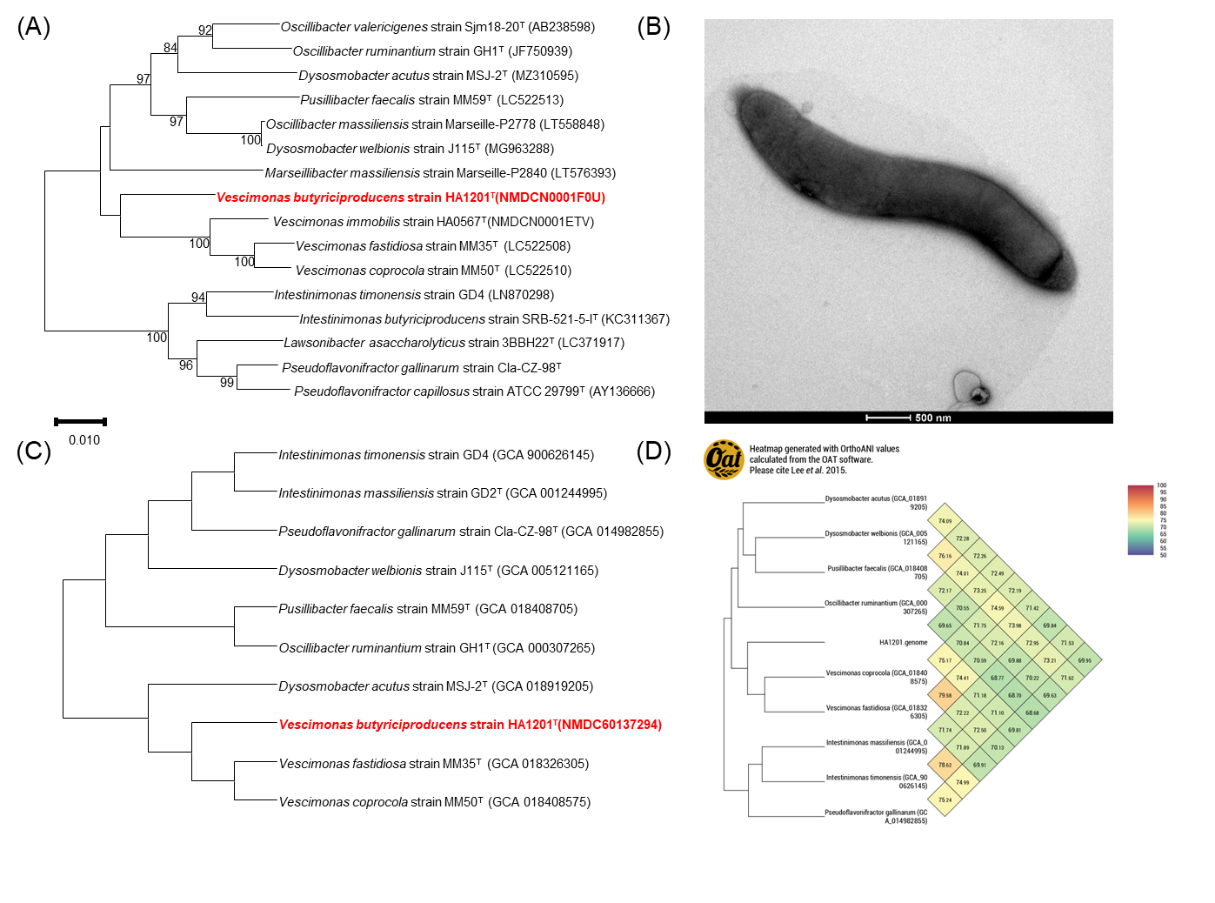


Figure ST-38. The Neighbor-joining phylogenetic tree based on 16S rRNA gene sequences (A) and the cell morphology (B) of strain HA1201^T^. GenBank accession numbers are given in parentheses. Percentages of bootstrap support are shown at branch nodes (values > 70 % are shown). Bootstrap value was 1000. Bar: 0.01 substitutions per nucleotide position. The phylogenomic tree based on genome-sequence-alignment (C) and OrthoANI heatmap (D) of strain HA1201^T^ and its neighbor species. Refseq ID of representative genomes are given in parentheses.

**Taxon 39: *Vescimonas immobilis* sp. nov**.

**Description of *Vescimonas immobilis* sp. nov.** (im.mo’bi.lis. L. fem. adj. *immobilis*, motionless, denoting the type strain in non-motile.)

The taxon 39, represented by strain HA0567^T^ (16S rRNA sequence accession number in NMDC is NMDCN0001ETV), is phylogenetically closest to *Vescimonas fastidiosa* MM35^T^, with 16S rRNA gene identity of 97.46%. Phylogenetic tree shows that strain HA0567^T^ clusters with other members of the genus *Vescimonas*, (Figure ST-39A), suggesting strain HA0567^T^ is a member of the genus *Vescimonas.* The genome of strain HA0567^T^ was sequenced and the NMDC (National Microbiology Data Center) accession number is NMDC60137286. Genome-based analysis showed that the ANI value of genomes of strain HA0567^T^ and *V. fastidiosa* MM35^T^ (GCA_018326305) is 79.85% and the dDDH estimation is 28.70%. According to the phylogenomic tree (Figure ST-39C), the ANIs and dDDH values of genomes between strain HA0567^T^ and phylogenomically neighbored genomes were calculated, and the highest ANI and dDDH values are 81.81% and 28.0%, respectively, between the query genome of *Vescimonas coprocola* MM50^T^ (GCA_018408575); and difference in mol% G+C between genomes of strain HA0567^T^ and *V. coprocola* MM50^T^ is 0.06; OrthoANI heatmap (Figure ST-39D) shows the phylogenomic status of the corresponding neighbor species based on OrthoANI values. Based on these results, we concluded that the strain HA0567^T^ represents of new species of the genus *Vescimonas*, and the name *Vescimonas immobilis* sp. nov. is proposed.

Cells are long rod-shaped (5.2 - 14.0 μm long × 0.5 μm wide, Figure ST-39B); cells have no flagella and non-motile. Growth to stable phase occurs after 48 h incubation in mmGAM medium at 37°C, pH = 7.2. No significant fermentation products detected. The G+C content of the type strain HA0567^T^ is 58.27 mol%. The type strain HA0567^T^ (=CGMCC 1.48288^T^) was isolated from the faeces of rheumatoid arthritis patients.


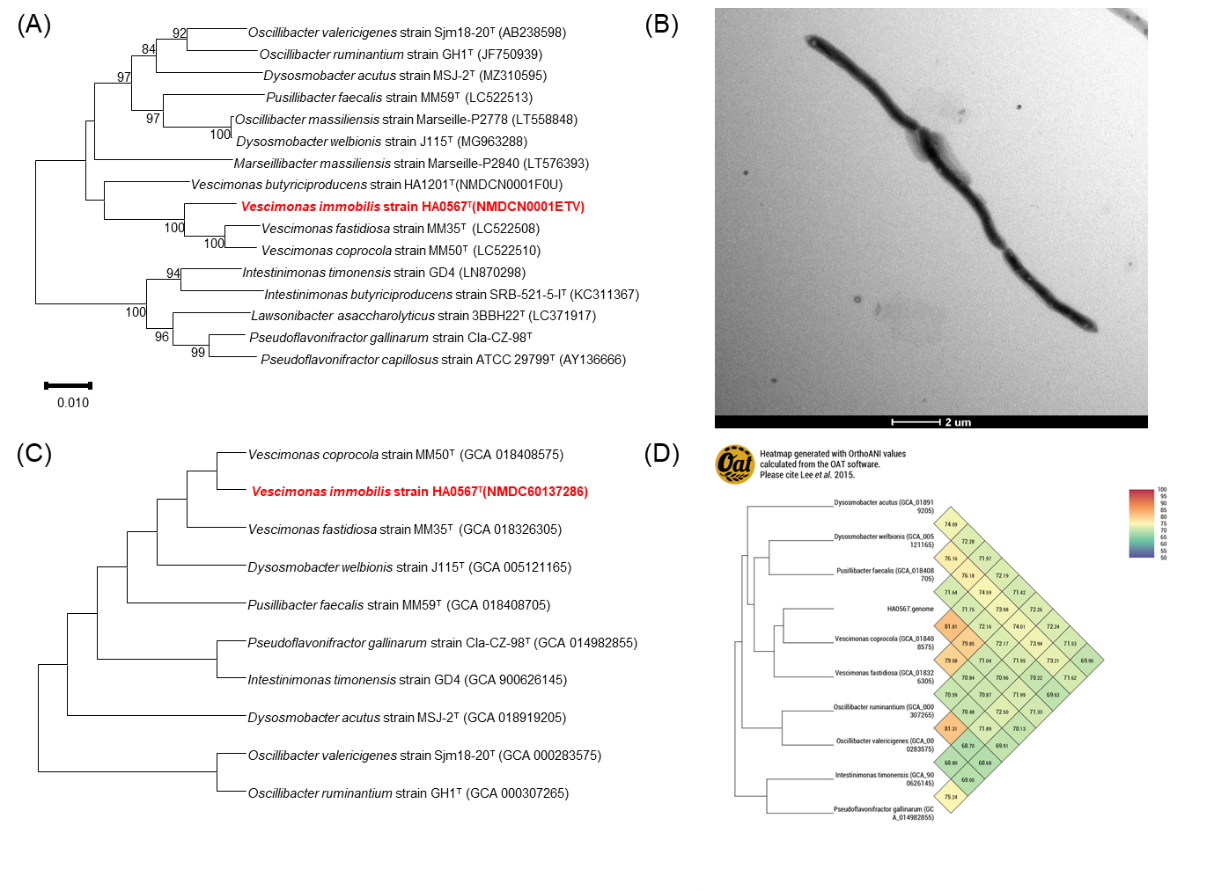


Figure ST-39. The Neighbor-joining phylogenetic tree based on 16S rRNA gene sequences (A) and the cell morphology (B) of strain HA0567^T^. GenBank accession numbers are given in parentheses. Percentages of bootstrap support are shown at branch nodes (values > 70 % are shown). Bootstrap value was 1000. Bar: 0.01 substitutions per nucleotide position. The phylogenomic tree based on genome-sequence-alignment (C) and OrthoANI heatmap (D) of strain HA0567^T^ and its neighbor species. Refseq ID of representative genomes are given in parentheses. **Taxon 40: *Peptoniphilus hominis* sp. nov**.

**Description of *Peptoniphilus hominis* sp. nov.** (ho’mi.nis. L. gen. masc. n. *hominis*, of a human being, indicating that the type strain was isolated from a human.)

The taxon 40, represented by strain HA1503^T^ (16S rRNA sequence accession number in NMDC is NMDCN0001F3J), is phylogenetically closest to *Peptoniphilus coxii* RMA 16157^T^, with 16S rRNA gene identity of 95.62%. Phylogenetic tree shows that strain HA1503^T^ clusters with other members of the genus *Peptoniphilus*, (Figure ST-40A), suggesting strain HA1503^T^ is a member of the genus *Peptoniphilus*. The genome of strain HA1503^T^ was sequenced and the NMDC (National Microbiology Data Center) accession number is NMDC60137299. Genome-based analysis showed that the ANI value of genomes of strain HA1503^T^ and *P. coxii* RMA 16157^T^ (GCA_001553115) is 75.25% and the dDDH estimation is 20.10%. According to the phylogenomic tree (Figure ST-40C), the ANIs and dDDH values of genomes between strain HA1503^T^ and phylogenomically neighbored genomes were calculated, and the highest ANI and dDDH values are 73.76% and 20.10% , respectively, between the query genome of *Peptoniphilus coxii* RMA 16157^T^ (GCA_001553115); And difference in mol% G+C between genomes of strain HA1503^T^ and *P. coxii* RMA 16157^T^ is 5.09; OrthoANI heatmap (Figure ST-40D) shows the phylogenomic status of the corresponding neighbor species based on OrthoANI values. Based on these results, we concluded that the strain HA1503^T^ represents of new species of the genus *Peptoniphilus*, and the name *Peptoniphilus qingdaonensis* sp. nov. is proposed.

Cells are oval-shaped (0.5 - 0.8 μm long × 0.5 μm wide) without flagella and non-motile (Figure ST-40B). Growth to stable phase occurs after 48 h incubation in mmGAM medium at 37°C, pH = 7.2. The main fermentation product is isovaleric acid, and small amounts of isobutyric acid and butyric acid can also be produced. The G+C content of the type strain HA1503^T^ is around 49.72 mol%. The type strain HA1503^T^(= CGMCC 1.48468^T^ = KCTC 25716^T^) was isolated from the faeces of rheumatoid arthritis patients.


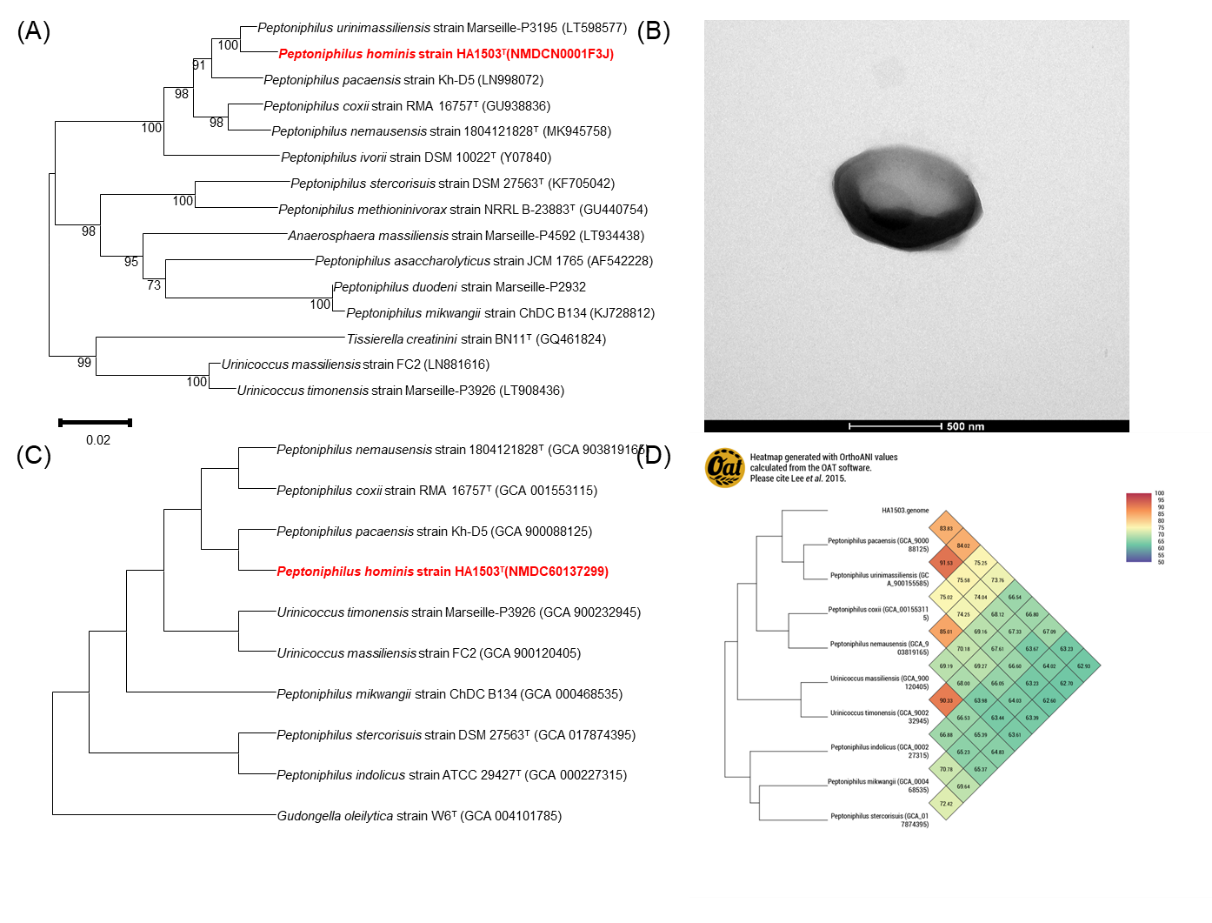


Figure ST-40. The Neighbor-joining phylogenetic tree based on 16S rRNA gene sequences (A) and the cell morphology (B) of strain HA1503^T^. GenBank accession numbers are given in parentheses. Percentages of bootstrap support are shown at branch nodes (values > 70 % are shown). Bootstrap value was 1000. Bar: 0.02 substitutions per nucleotide position. The phylogenomic tree based on genome-sequence-alignment (C) and OrthoANI heatmap (D) of strain HA1503^T^ and its neighbor species. Refseq ID of representative genomes are given in parentheses.

**Taxon 41: *Butyricimonas recta* sp. nov**.

**Description of *Butyricimonas recta* sp. nov.** (rec’ta. L. fem. part. adj. *recta*, straight, indicating that the type strain is straight rod-shaped.)

The taxon 41, represented by strain HA1200^T^ (16S rRNA sequence accession number in NMDC is NMDCN0001F0T), is phylogenetically closest to *Butyricimonas paravirosa* 214-4^T^, with 16S rRNA gene identity of 97.99%. Phylogenetic tree shows that strain HA1200^T^ clusters with other members of the genus *Butyricimonas,* (Figure ST-41A), suggesting strain HA1200^T^ is a member of the genus *Butyricimonas.* The genome of strain HA1200^T^ was sequenced and the NMDC (National Microbiology Data Center) accession number is NMDC60137293. Genome-based analysis showed that the ANI value of genomes of strain HA1200^T^ and *B. paravirosa* 214-4^T^ (GCA_014647355) is 93.35% and the dDDH estimation is 55.90%. According to the phylogenomic tree (Figure ST-41C), the ANIs and dDDH values of genomes between strain HA1200^T^ and phylogenomically neighbored genomes were calculated, and the highest ANI and dDDH values are 96.96% and 34.0%, respectively, between the query genome of *Butyricimonas faecalis* H184^T^ (GCA_003991565); and difference in mol% G+C between genomes of strain HA1200^T^ and *B. faecalis* 214-4^T^ is 0.64; OrthoANI heatmap (Figure ST-41D) shows the phylogenomic status of the corresponding neighbor species based on OrthoANI values. Based on these results, we concluded that the strain HA1200^T^ represents of new species of the genus *Butyricimonas*, and the name *Butyricimonas recta* sp. nov. is proposed.

Cells are rod-shaped with square ends (1.0 - 3.0 μm long × 0.6 μm wide, Figure ST-41B); cells appear singly without flagella and non-motile. Growth to stable phase occurs after 48 h incubation in mmGAM medium at 37°C, pH = 7.2. The main fermentation products are small amount of propionic, isobutyric, butyric and isovaleric acid. The G+C content of the type strain HA1200^T^ is around 62.05 mol%. The type strain HA1200^T^(= CGMCC 1.48382^T^ = KCTC 25703^T^) was isolated from the faeces of rheumatoid arthritis patients.


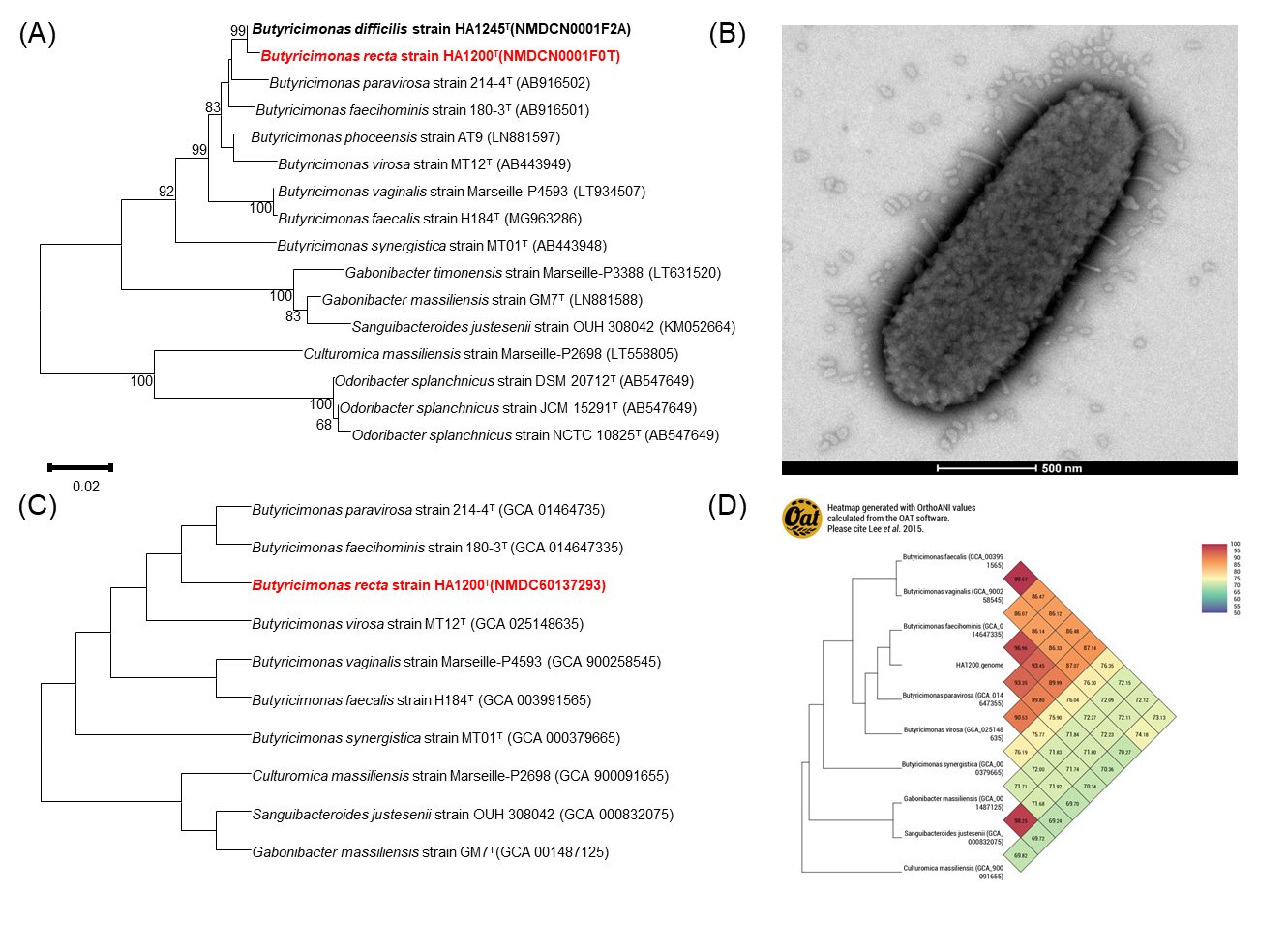


Figure ST-41. The Neighbor-joining phylogenetic tree based on 16S rRNA gene sequences (A) and the cell morphology (B) of strain HA1200^T^. GenBank accession numbers are given in parentheses. Percentages of bootstrap support are shown at branch nodes (values > 70 % are shown). Bootstrap value was 1000. Bar: 0.02 substitutions per nucleotide position. The phylogenomic tree based on genome-sequence-alignment (C) and OrthoANI heatmap (D) of strain HA1200^T^ and its neighbor species. Refseq ID of representative genomes are given in parentheses. **Taxon 42: *Parabacteroides propionicigenes* sp. nov.**

**Description of *Parabacteroides propionicigenes* sp. nov.** (pro.pi.o.ni.ci’ge.nes. N.L. neut. n. *acidum propionicum*, propionic acid; Gr. suff. -*genes*, producing; from Gr. ind. v. *gennaô*, to produce; N.L. part. adj. *propionicigenes*, propionic acid producing)

The taxon 42, represented by strain HA3406^T^ (16S rRNA sequence accession number in NMDC is NMDCN0001F7I), is phylogenetically closest to *Parabacteroides gordonii* MS-1^T^*,* with 16S rRNA gene identity of 96.79%. Phylogenetic tree shows that strain HA3406^T^ clusters with other members of the genus *Parabacteroides*, (Figure ST-42A), suggesting strain HA3406^T^ is a member of the genus *Parabacteroides*. The genome of strain HA3406^T^ was sequenced and the NMDC accession number is NMDC60144119. Genome-based analysis showed that the ANI value of genomes of strain HA3406^T^ and *Parabacteroides gordonii* MS-1^T^ (GCA_000969825) is 84.44% and the dDDH estimation is 29.2%. According to the phylogenomic tree (Figure ST-42C), the ANI and dDDH values of genomes between strain HA3406^T^ and phylogenomically neighbored genomes were calculated, and the highest ANI and dDDH values are 84.44% and 29.2% , respectively, between the query genome of *Parabacteroides gordonii* MS-1^T^ (GCA_000969825); and difference in mol% G+C between genomes of strain HA3406^T^ and *P. gordonii* MS-1^T^ is 1.33; OrthoANI heatmap (Figure ST-42D) shows the phylogenomic status of the corresponding neighbor species based on OrthoANI values. Based on these results, we concluded that the strain HA3406^T^ represents of new species of the genus *Parabacteroides*, and the name *Parabacteroides propionicigenes* sp. nov. is proposed.

Cells are rod-shaped with oval ends (1.8 -4.4 μm long × 1.0 μm wide, Figure ST-42B); cells appear singly without flagella and no motile. Growth to stable phase occurs after 24 h incubation in mGAM medium at 37°C, pH=7.2. The main fermentation products are acetic, propionic, isobutyric, and isovaleric acid, and small amounts of butyric, and valeric acid can also be produced. The G+C content of the type strain HA3406^T^ is around 43.11 mol%. The type strain HA3406^T^ (= CGMCC 1.48595^T^ = KCTC 25789^T^) was isolated from the faeces of rheumatoid arthritis patients.


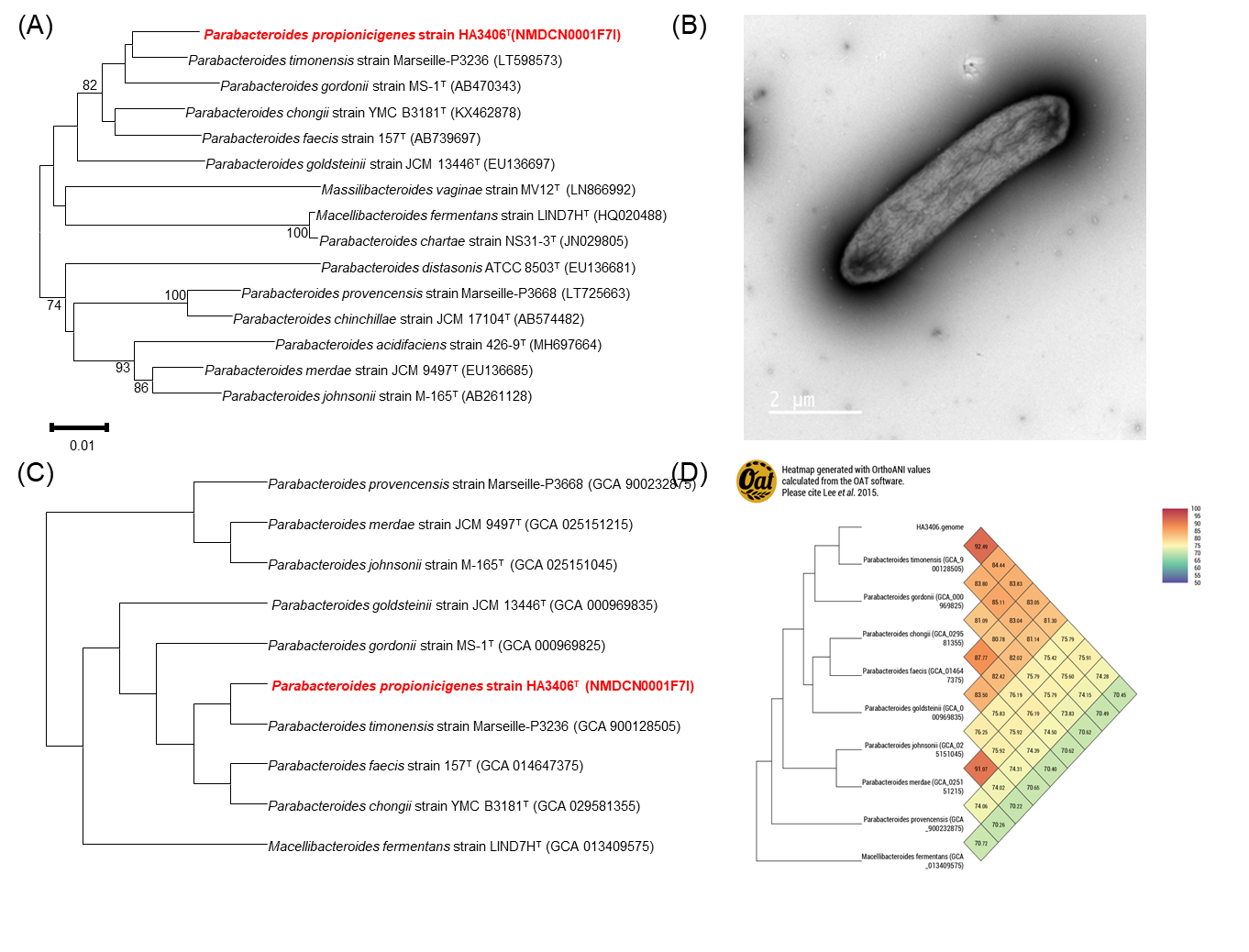


Figure ST-42. The Neighbor-joining phylogenetic tree based on 16S rRNA gene sequences (A) and the cell morphology (B) of strain HA3406^T^. GenBank accession numbers are given in parentheses. Percentages of bootstrap support are shown at branch nodes (values > 70 % are shown). Bootstrap value was 1000. Bar: 0.01 substitutions per nucleotide position. The phylogenomic tree based on genome-sequence-alignment (C) and OrthoANI heatmap (D) of strain HA3406^T^ and its neighbor species. Refseq ID of representative genomes are given in parentheses.

**Taxon 43: *Pyramidobacter arthritidis* sp. nov**.

**Description of *Pyramidobacter arthritidis* sp. nov.** (ar.thri’ti.dis. Gr. fem. n. *arthron*, joint; N.L. fem. n. suff. -*itis*, inflammation; N.L. gen. fem. n. *arthritidis*, of arthritis, denoting the type strain was isolated from the faeces of a rheumatoid arthritis patient.)

The Taxon 43, represented by strain HA0566^T^ (16S rRNA sequence accession number in NMDC is NMDCN0001ETU), is phylogenetically closest to *Pyramidobacter porci* SM-530-WT-4B^T^, with 16S rRNA gene identity of 98.50%. Phylogenetic tree shows that strain HA0566^T^ clusters with other members of the genus *Pyramidobacter*, (Figure ST-43A), suggesting strain HA0566^T^ is a member of the genus *Pyramidobacter*. The genome of strain HA0566^T^ was sequenced and the NMDC (National Microbiology Data Center) accession number is NMDC60137285. Genome-based analysis showed that the ANI value of genomes of strain HA0566^T^ and *P. porci* SM-530-WT-4B^T^ (GCA_009695745) is 96.17% and the dDDH estimation is 67.10 %. According to the phylogenomic tree (Figure ST-43C), the ANIs and dDDH values of genomes between strain HA0566^T^ and phylogenomically neighbored genomes were calculated, and the highest ANI and dDDH values are 96.17% and 67.10%, respectively, between the query genome of *P. porci* SM-530-WT-4B^T^; and difference in mol% G+C between genomes of strain HA0566^T^ and *P. porci* SM-530-WT-4B^T^ is 0.19; OrthoANI heatmap (Figure ST-43D) shows the phylogenomic status of the corresponding neighbor species based on OrthoANI values. Based on these results, we concluded that the strain HA0566^T^ represents of new species of the genus *Pyramidobacter*, and the name *Pyramidobacter arthritidis* sp. nov. is proposed.

Cells are fusiform or short rod-shaped with rounded ends (1.2 - 2.4 μm long × 0.8 μm wide, Figure ST-43B); cells appear singly without flagella and non-motile. Growth to stable phase occurs after 48 h incubation in mmGAM medium at 37°C, pH = 7.2. The main fermentation products are small amount of isobutyric and isovaleric acid. The G+C content of the type strain HA0566^T^ is around 59.84 mol%. The type strain HA0566^T^ (= CGMCC 1.48287^T^ = KCTC 25698^T^) was isolated from the faeces of rheumatoid arthritis patients.


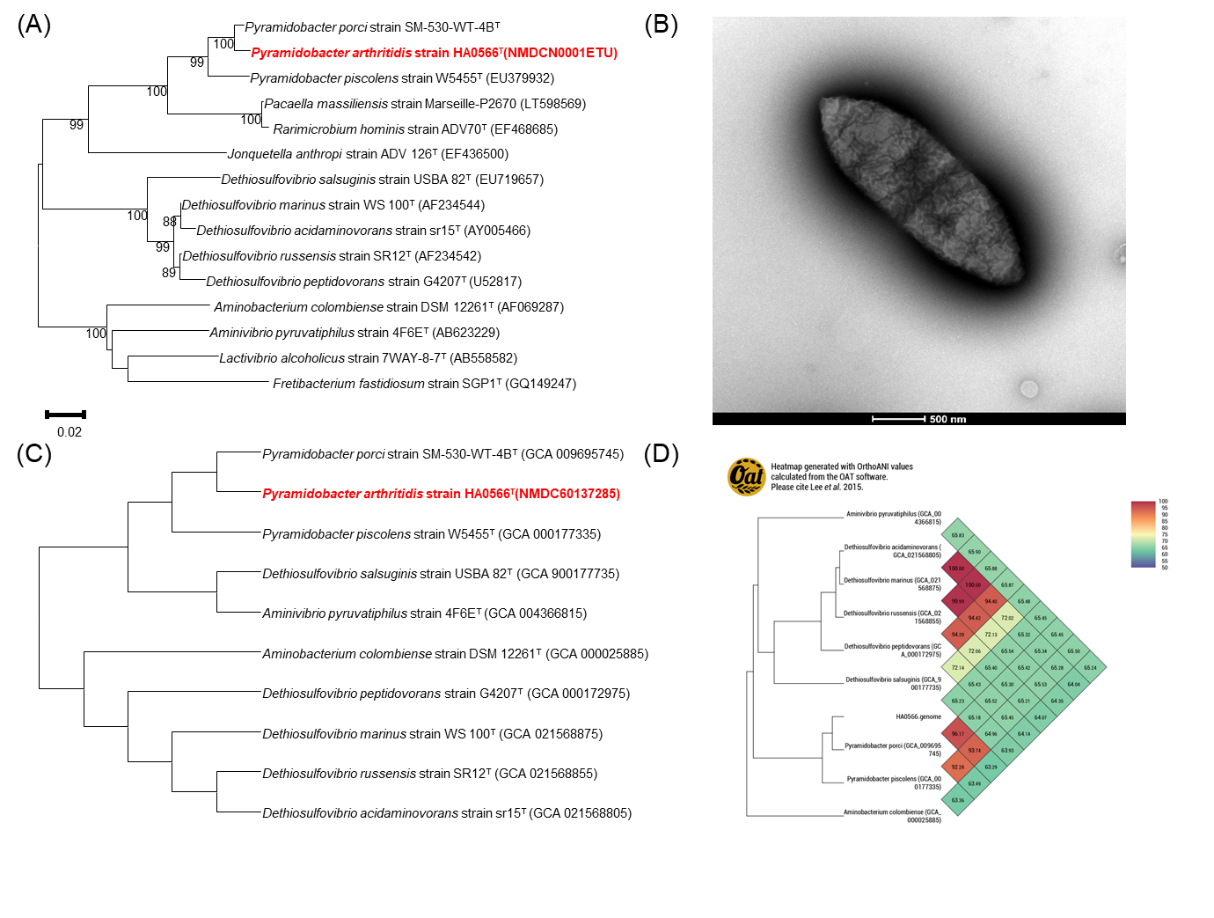


Figure ST-43. The Neighbor-joining phylogenetic tree based on 16S rRNA gene sequences (A) and the cell morphology (B) of strain HA0566^T^. GenBank accession numbers are given in parentheses. Percentages of bootstrap support are shown at branch nodes (values > 70 % are shown). Bootstrap value was 1000. Bar: 0.02 substitutions per nucleotide position. The phylogenomic tree based on genome-sequence-alignment (C) and OrthoANI heatmap (D) of strain HA0566^T^ and its neighbor species. Refseq ID of representative genomes are given in parentheses.
